# Synthesis and Structure of Neopentyl Sodium: A Hydrocarbon‐Soluble Reagent for Controlled Sodiation of Non‐Activated Substrates

**DOI:** 10.1002/anie.202511492

**Published:** 2025-07-29

**Authors:** David E. Anderson, Lorraine A. Malaspina, Simon Grabowsky, Eva Hevia

**Affiliations:** ^1^ Departement für Chemie Biochemie und Pharmazie Universität Bern Freiestrasse 3 Bern 3012 Switzerland

**Keywords:** Metalation, Organometallic reagents, Organosodium, Structure elucidation, Structure–activity relationships

## Abstract

While recent studies have shown that organosodium reagents can offer excellent promise in organic synthesis, their widespread applications can be hindered by their limited solubility in organic solvents, lack of stability, and uncontrolled reactivity. Improving this immature area, here we report the structure of unsolvated neopentyl sodium, which exhibits a unique discrete tetrameric motif and good solubility in hexane. Combining spectroscopic and crystallographic studies with computational bonding analysis, key insights into its constitution and stability have been gained. Expanding its synthetic potential, supporting neopentyl sodium with Lewis donor PMDETA (*N,N,N′,N″,N″*‐pentamethyldiethylenetriamine) allows for the direct metalation of challenging non‐activated arenes and alkenes, including the regioselective *meta*‐functionalisation of selected 1,3‐disubstituted alkyl arenes. Shedding light on sodium‐mediated metalation protocols, key organometallic intermediates have been isolated and structurally defined, which can subsequently be intercepted with CO_2_ or a boron electrophile to be directly used in Pd‐catalysed Suzuki cross coupling reactions.

## Graphical abstract

Displaying strong controlled metalating power, hydrocarbon‐soluble sodium neopentyl can deprotonate challenging non‐activated arenes and alkenes, with sodiated intermediates successfully undergoing onward functionalisation reactions. By characterising key reaction intermediates, and running computational studies on models, insight has been gained on the structure, bonding, and special reactivity of this promising new reagent.

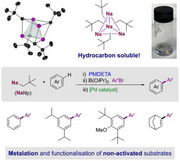

## Introduction

Polar organolithium reagents constitute one of the most widely used class of organometallic reagents employed for functionalisation of organic molecules.^[^ ^1^, ^2^, ^3^, ^4^ ^]^ Much of their success has come from having an in depth understanding of the constitutions of key organometallic intermediates leading to their rational utilisation.^[^ ^5^, ^6^, ^7^, ^8^, ^9^ ^]^ However, despite the common usage of organolithiums, organosodium reagents have started to appear in synthetic endeavours, driven by attempts to replace scarcer and more expensive lithium with the more abundant and sustainable sodium metal.^[^ ^10^, ^11^, ^12^, ^13^, ^14^, ^15^ ^]^ Promisingly, these studies have revealed that some sodium organometallics can also exhibit superior but controllable reactivities.^[^ ^14^, ^15^, ^16^ ^]^

Interestingly, organosodium compounds have been known for over 150 years since Wanklyn's 1858 report of ethyl sodium via reaction of ethyl iodide with sodium metal.^[^ ^17^ ^]^ While organosodium chemistry was neglected for many years, seminal work from Morton and Finnegan in the 1950–60s hinted at their potential in metalation chemistry using *n‐*pentyl sodium for the sodiation of non‐activated substrates (p*K*a values > 40) such as benzene and assorted alkenes.^[^ ^18^, ^19^, ^20^, ^21^, ^22^, ^23^, ^24^, ^25^ ^]^ Here, the alkyl sodium was accessed via *n‐*pentyl chloride and sodium metal, affording a reportedly insoluble mixture of sodium chloride and the organosodium reagent. In general, the sensitive nature, poor solubility, and limited solvent tolerance of in situ sodiated species (intermediates) meant they could not be isolated in pure form or characterised, with these reactions only monitored via CO_2_ electrophilic interception, producing moderate to good yields of the relevant carboxylic acids (15%–69% yields) with reaction times ranging from 1 h to several days.

Considering the operational challenges of the preparation of alkyl sodium reagents, their use in organic synthesis has continued to be very limited until recently, where new methods have been developed using alternative sodium sources. Knochel has shown that the soluble but thermally unstable 2‐ethylhexyl sodium can be made by passing 2‐ethylhexyl chloride through a sodium packed column under continuous flow conditions.^[^ ^26^ ^]^ This in situ formed alkyl sodium was found to be a powerful base capable of sodiating a variety of toluene derivatives for further functionalisation, as well as being a competent reagent for sodium halogen exchange applications.^[^ ^27^ ^]^

**Scheme 1:**
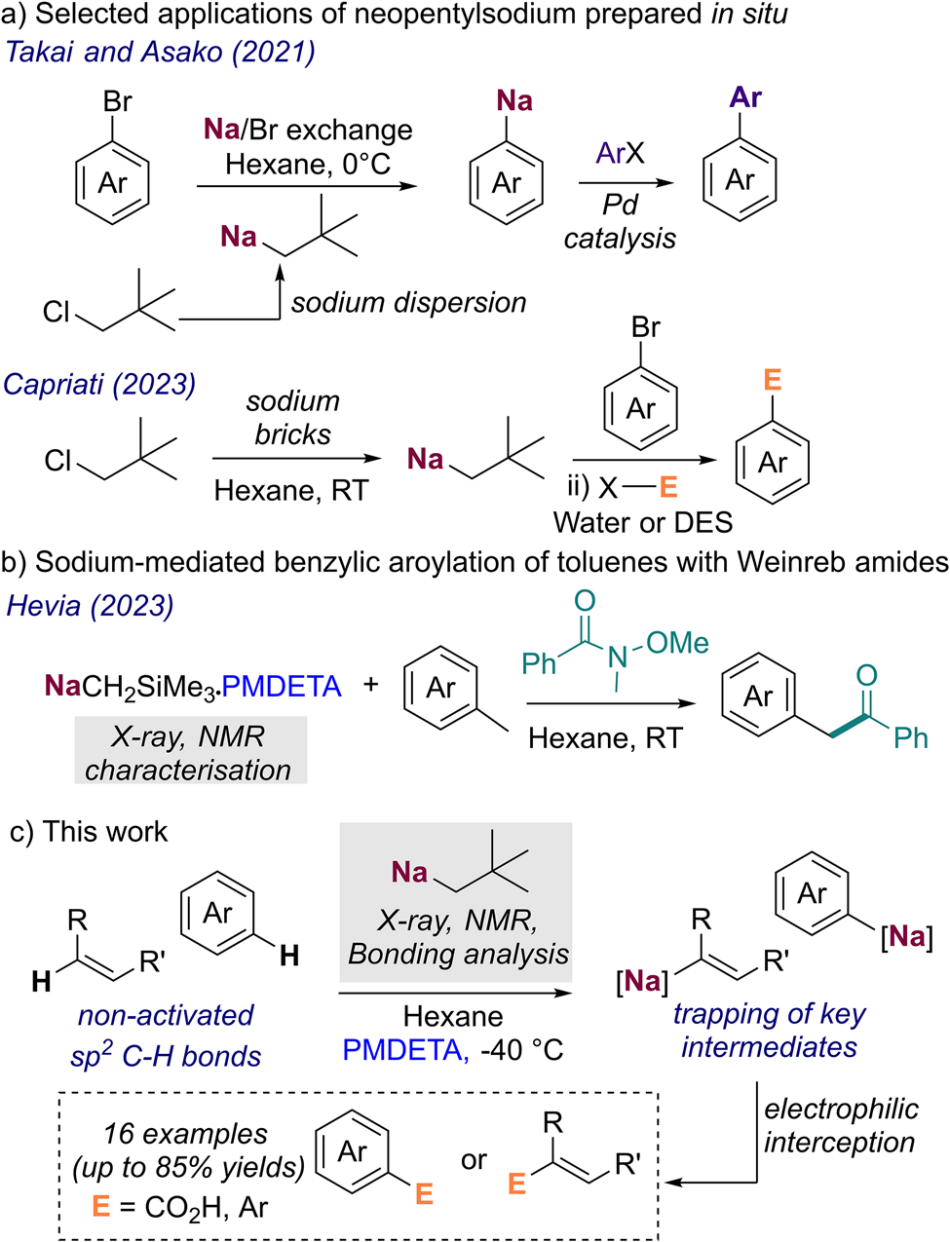
Selected recent uses of alkyl sodium reagents in synthesis. a) Sodium‐bromine exchanges and subsequent functionalisation using in situ generated NaNp; b) Sodium‐mediated benzylic aroylation of toluenes using Weinreb amides; and c) This work: synthesis and characterisation of NaNp and its surprising suitability for controlled C─H metalation of non‐activated substrates.

**Scheme 2:**
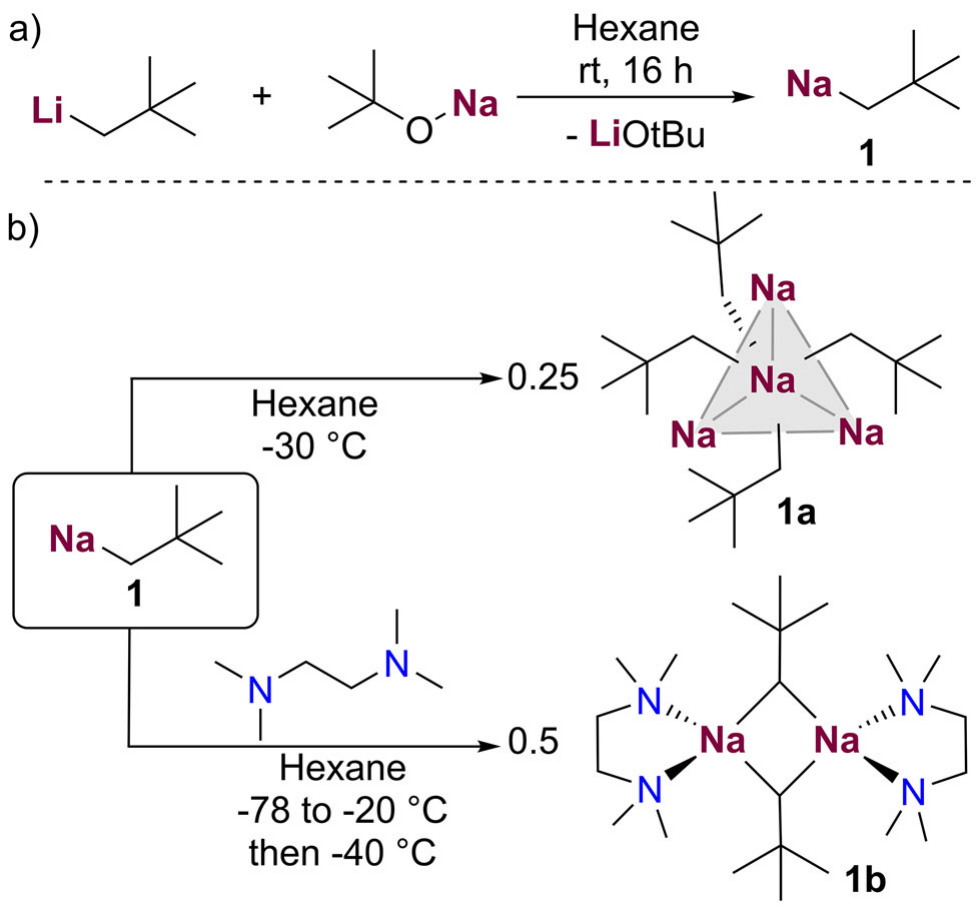
a) Synthesis of neopentyl sodium (**1**) from neopentyl lithium; b) conditions for crystallisation of unsolvated NaNp [{NaCH_2_ *t*Bu}_4_] (**1a**) and its TMEDA adduct [{(TMEDA)NaCH_2_ *t*Bu}_2_] (**1b**).

It has been reported that combining finely divided sodium in dispersion or brick form with neopentyl chloride produces a potent but surprisingly soluble alkyl sodium base which can be used in sodium/bromine exchange reactions with a wide range of bromoarenes. This has been demonstrated by Takai and Asako, where the generated NaAr intermediates can be used in Pd‐ catalysed cross couplings (Scheme 1a);^[^ ^28^, ^29^, ^30^ ^]^ and by Capriati who reported such NaAr species react with a range of electrophiles using water or deep eutectic solvents (DESs) under air.^[^ ^31^ ^]^ Remarkably, while neopentyl sodium (NaNp) seems to be key in these studies, its constitution or aggregation has not been pursued (Scheme 2).

Using a different method to access alkyl sodium reagents, our group has recently reported the use of NaCH_2_SiMe_3_, isolated as a pure reagent from the reaction of LiCH_2_SiMe_3_ and NaO*t*Bu for the benzylic aroylation of toluenes using Weinreb amides (Scheme 1b).^[^ ^16^, ^32^ ^]^ Through the successful isolation of key organometallic intermediates, this study elucidated the key role of Lewis donor PMDETA to promote these transformations, enhancing the kinetic reactivity of these intermediates towards both metalation and nucleophilic addition. Related to this work, Lu has also shown the ability of NaCH_2_SiMe_3_ to promote methylenations of ketones and aldehydes.^[^ ^14^ ^]^ Furthermore, early this year, Thomas has reported that pyridine can be regioselectively C4‐functionalised by direct metalation with *n‐*butylsodium under carefully controlled reaction conditions.^[^ ^33^ ^]^

Putting alkylsodium chemistry on a more practicable basis, here we isolate and fully characterise solvent‐free NaNp prepared by simple salt metathesis of LiNp with NaO*t*Bu. Combining X‐ray crystallographic and spectroscopic analysis with bonding analysis calculations^[^ ^34^ ^]^ (QTAIM,^[^ ^35^ ^]^ NCI^[^ ^36^ ^]^ and ELI‐D^[^ ^37^ ^]^), we shed light on the constitution, solubility, and aggregation of this reagent. Its ability to mediate direct Na‐H exchange reactions is also demonstrated by probing its reactivity towards a wide range of non‐activated arenes and alkenes, disclosing a superior metalating power to those observed by conventional organolithium reagents or even by Lochmann–Schlosser super basic combinations.

## Results and Discussion

Building on our previous studies,^[^ ^38^ ^]^ NaNp (**1**) was prepared by salt‐metathesis by treating neopentyl lithium with sodium *tert*‐butoxide in hexane which led to the isolation **1** as a white solid in a 53% yield (see Supporting Information for details). Note that this moderate isolated yield is due to the high hexane solubility of **1**, which is remarkable since it contrasts markedly with the poor solubility of other alkyl sodium compounds such as Na*n*Bu or NaCH_2_SiMe_3_ also prepared using this method.^[^ ^38^, ^39^ ^]^ In fact, we found that **1** can be stored as a 0.5 M solution in hexane for at least 3 days at 20 °C without observing decomposition. This unique solubility also allowed us to fully characterise **1** in deuterated cyclohexane solutions by NMR spectroscopy. Its ^1^H NMR spectrum showed a distinct signal at −0.71 ppm assignable to the Na‐C*H*_2_ group, which is slightly more shielded than the analogous signal in congeneric LiNp (−0.61 ppm).

**Figure 1:**
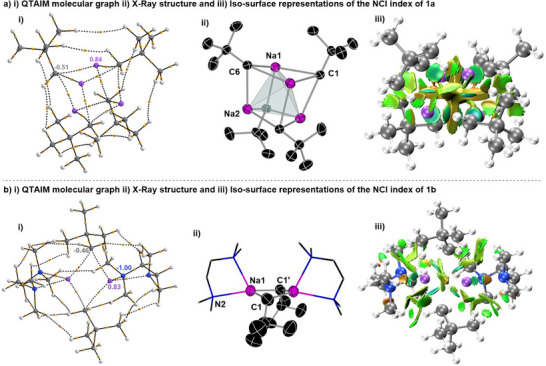
i) QTAIM molecular graph of **1a** a) and **1b **b) with bond paths, bond critical points (orange balls) and the most relevant atomic charges (sodium–purple, carbon–grey, nitrogen–blue, hydrogen–white). ii) Molecular structure of **1a** a) and **1b **b) with 50% probability, displacement ellipsoids. All H atoms have been omitted for clarity, and TMEDA ligands shown as frames; selected atomic distances from crystal structure determination (isolated‐molecule optimisation in parenthesis) **1a**) Na1─C1 = 2.6733(20) Å (2.575 Å), Na1─C6 = 2.666(2) Å (2.546 Å), Na2─C1 = 2.6446(20) Å (2.546 Å), Na2─C6 = 2.6324(21) Å (2.575 Å). **1b**) Na1─C1 2.359(9) Å (2.538 Å), Na1─C1′ 2.823(9) Å (2.567 Å). iii) Iso‐surface representations of the NCI index **1a** a) and **1b** b). *sign*(λ_2_) · ρ is mapped onto the reduced density gradient iso‐surface (*s*(r) = 0.5). Turquoise surface parts refer to attractive forces, orange to repulsive forces. Green indicates non‐bonding van der Waals interactions (sodium–purple, carbon–grey, nitrogen–blue, hydrogen–white).

When forming a solution of a mixture of **1** in the presence of LiO*t*Bu, a crop of colourless needle‐like crystals of [{NaCH_2_ *t*Bu}_4_] (**1a**) were formed. In addition, we found that upon cooling a solution of pure neopentyl sodium in hexane to −30 °C, the same crystalline product was attained. X‐ray crystallographic studies of **1a** revealed a discrete tetrameric structure (Figure 1a ii) which can be described as a distorted tetrahedron of four sodium atoms, with each triangular Na_3_ face capped by a methanide carbon atom to complete a distorted Na_4_C_4_ heterocubane. While this tetrameric motif bears a resemblance to that exhibited by the classical organometallic reagent *t*BuLi,^[^ ^40^ ^]^ its discrete motif is unusual when compared with the structures of other unsolvated alkyl sodium complexes.^[^ ^41^, ^42^, ^43^ ^]^

A close inspection of the geometrical parameters in **1a** show that the Na─C bond distances (range 2.6324(21) to 2.6733(20) Å)] are within the same ballpark as those previously reported for related alkyl sodium complexes.^[^ ^32^, ^41^, ^44^ ^]^ The non‐bonding Na…Na distances in **1a** (spanning from 3.0500(11) to 3.2244(10) Å) compare well with those found for unsolvated, silyl‐substituted [{NaCH_2_SiMe_3_}_4_]_∞_,^[^ ^41^ ^]^ which also comprises {Na_4_C_4_} tetramers, although, in this case, these units interact intermolecularly with each other in a supramolecular arrangement.^[^ ^43^ ^]^ Thus, in **1a** the shortest Na─C atomic distance between two different oligomeric units is 3.5516(20) Å; whereas in [{NaCH_2_SiMe_3_}_4_]_∞_ it contracts to 2.7872(17) Å. This discrete tetrameric structure of **1a** is retained in deuterated cyclohexane solutions, as evidenced by ^1^H DOSY NMR studies (estimated FW = 358 g mol^−1^, 4% error, see Supporting Information for details),^[^ ^45^, ^46^ ^]^ which explains its remarkable hydrocarbon solubility.

Solvated adducts of **1a** could be formed by adding equimolar amounts of TMEDA (*N,N,N′,N′*‐tetramethylethylenediamine) or PMDETA to solutions of NaNp in hexane. These proved highly reactive decomposing above −40 °C (see Supporting Information for details). While the PMDETA adduct decomposed on attempts to isolate it, the TMEDA adduct was more successful, which could be isolated at −40 °C as colourless crystals, that were amenable to X‐ray crystallographic characterisation. Thus, [{TMEDA)NaCH_2_ *t*Bu}_2_] (**1b**) (Figure 1b ii) adopts a centrosymmetric dimeric motif, containing a nearly planar four‐membered (NaC)_2_ ring (sum of internal angles, 358°) where the neopentyl groups bridge the Na atoms. The Na1─C1 bond is noticeably shorter to those found in **1a** [2.359(9) versus a mean value of 2.6528 Å] which can be rationalised in terms of the different coordination number of the CH_2_ unit in both compounds. Interestingly the remaining Na─C bond distance in **1b** is significantly elongated [Na1─C1’ 2.823(9) Å] and both neopentyl groups lie on the same side of the ring, although there is not an obvious explanation to account for this preferred cisoid conformation.^[^ ^47^ ^]^ This dimeric structure contrasts with that reported for the related TMEDA adduct of NaCH_2_SiMe_3_ which forms a polymeric chain arrangement,^[^ ^41^ ^]^ indicating that the steric demands of the neopentyl group seem to favour formation of smaller aggregates. The dimeric aggregation **1b** appears to be retained in deuterated cyclohexane solutions as indicated by ^1^H DOSY NMR experiments (estimated weight of 377 g mol^−1^, 11% error, see Supporting Information for details.).^[^ ^45^, ^46^ ^]^

**Figure 2:**
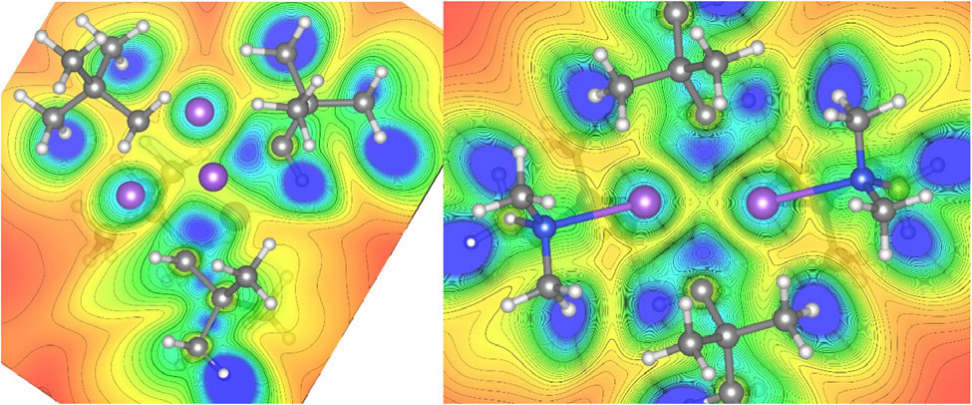
Contour map of ELI‐D on a cut‐plane through the central Na cluster for **1a** (left) and **1b** (right). Colour range from ELI‐D = 0 (red, no localisation) to 2 (blue, high localisation), contour interval 0.1. Atom colours: purple = Na, grey = C, blue = N, white = H.

Intrigued by the structural features of **1a** and **1b** we next sought to further understand the nature of the bonding of the complexes with the aid of computational techniques, using a combination of QTAIM and NCI [Figure 1i and iii] and electron localisability indicator (ELI‐D) analyses (Figure 2). Remarkably, for both compounds it was deduced that inclusion of the empirical dispersion correction in the isolated‐molecule geometry optimisation (B3LYP/def2‐TZVP) leads to a significant total stabilisation of the molecular aggregates by 443.8 and 401.3 kJ mol^−1^ for **1a** and **1b,** respectively. This is caused by conformational rearrangements in the methyl groups of both the neopentyl and TMEDA groups (for **1b**) upon activating dispersion correction (see also Figure S66), so that the ligands form a kind of organic skin around the Na atoms stabilised by London dispersion.

The Me–Me attractive dispersion interactions can be visualised as bond paths between hydrogen atoms in the QTAIM molecular graphs (Figure 1 i), which are channels of interaction between atoms in the electron density.^[^ ^48^ ^]^ While for **1a** two H─H bond paths between each pair of adjacent Np groups has been found, it should be noted that these H─H bond paths are absent in its electron‐density distribution when empirical dispersion correction is not included (Figure S66 in the Supporting Information). NCI analysis provides an alternative visualisation of these van‐der‐Waals‐type interactions (Figure 1 iii), displaying attractive dispersion forces between the methyl groups of the Np ligands in both complexes around the central sodium core (green colour patches in Figure 1 iii). Collectively, these findings emphasise the profound effect of dispersion to rearrange the conformation of the alkyl groups, providing significant stabilisation by their mutual interactions to favour the formation of these unusually low‐aggregated organosodium complexes.^[^ ^49^, ^50^, ^51^ ^]^

In contrast to the geometrical parameters obtained by the X‐ray crystallographic studies, the isolated‐molecule optimised geometries are much more symmetrical concerning the central Na cluster (optimised Na─C bond distances in the caption of Figure 1) as expected when the crystal field of discrete neighbours is lost. The Na─C distances also become more similar between both compounds (2.54–2.58 Å) upon symmetrisation. Consistent with the discussion of the experimental geometries, for **1a** each of the CH_2_ groups of the Np ligands is located centrally above one of the Na_3_ triangles from the faces of the Na_4_ tetrahedron, bridging those three Na atoms with three separate bond paths, to form a distorted cubic structure (Figure 1a,i). Similarly, for **1b**, each CH_2_ lies close to the Na…Na axis, to form a NaCNa bridge indicated by two bond paths to the two Na atoms (Figure 1b,i).

The delocalisation index (DI) that measures the exchange of electron pairs between two atomic basins in QTAIM; can be considered as a bond order.^[^ ^52^ ^]^ It is independent of the existence of bond paths, so it can measure the interaction of the Na atoms with each other. However, none of the Na–Na DI's in **1a** and **1b** exceeds a value of 0.005, indicating that their mutual interaction can be considered negligible. In contrast, the DI values for the C–Na interactions are significant although still low. Thus, in **1b**, the DI sum for each bifurcated C–Na_2_ interaction is 0.24, whereas in **1a**, a value of 0.30 was found for the C–Na_3_ units. These findings indicate that the covalent contributions to these bonding interactions are low, which can be rationalised by the fact that the carbon atoms cannot become hypervalent although they are penta‐ or hexa‐coordinated. Furthermore, the NCI analysis of the complexes shows that there are significant areas of repulsion in the central spaces between the Na atoms (Figure 1 iii).

Atomic charges for **1a** and **1b** were calculated by QTAIM (Figure 1,i), finding that despite their different aggregation, almost identical values were found for the Na atoms (0.84 and 0.83 e, respectively). For the CH_2_ units of the Np ligands values close to half a negative charge were found (−0.51 and −0.46 e for **1a** and **1b** respectively), leaving an overall positive charge which is counterbalanced in **1a** by the methyl groups which are slightly negatively charged. In **1b**, the TMEDA nitrogen atoms overcompensate this residual charge of the Na/C core with −1.00 e per N atom, which in turn means that the methyl groups are each slightly positively charged.

ELI‐D distributions analysis was also performed (Figure 2 and see Supporting Information for details) which shows high localisation of the electron pairs on the CH_2_ units of the Np groups and also how these pairs are directed within **1a** and **1b**. For **1a** the basins of these electron pairs share boundaries in topological analysis with three Na atoms and their parent C (Figure 2, left); whereas for **1b** they are shared with one C and only two sodium atoms (Figure 2, right). These findings are consistent with the description of the bonding present in **1a** and **1b** as [4c, 2e‐] and [3c,2e‐] respectively.

**Scheme 3:**
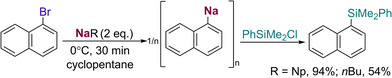
Comparative reactivity of NaNp and Na*n*Bu towards Na/Br exchange of 2‐bromonapthalene.

Turning back to our experimental studies, we pondered if the exceptional hydrocarbon solubility of **1** could be leveraged to access reactivity that would be otherwise unattainable for an insoluble alkyl sodium such as Na*n*Bu.^[^ ^38^ ^]^ Thus we first tested the reactivity of isolated donor free NaNp towards Na/Br exchange using 1‐bromonaphthalene as a model substrate. Using cyclopentane as a solvent, under mild reaction conditions (0 °C, 30 min), rapid formation of 1‐napthylsodium occurs which can be intercepted with PhSiMe_2_Cl to form dimethyl(naphthalen‐1‐yl)(phenyl)silane in a 94% yield (see Scheme 3 and Supporting Information for details). This result aligns well with previous studies by Asako and Takai on the use of NaNp, prepared in situ using Na dispersions and NpCl (Scheme 1a).^[^ ^29^ ^]^ Contrastingly using Na*n*Bu, under exactly the same reaction conditions, furnished the relevant silane product in a diminished 54% yield (Scheme 3).

We next investigated the reactivity of NaNp towards C─H metalation, an area in which other organosodium reagents have also shown promise. We focused on non‐activated arenes and alkenes (in terms of p*K*a values) which are challenging to metalate with conventional s‐block metal bases, using benzene (calculated p*K*a about 44)^[^ ^53^ ^]^ as a model substrate. Disappointingly, addition of 1.5 equivalents of benzene to **1a** in hexane at room temperature, followed by interception with CO_2_ furnished only traces of carboxylic acid **2a** (Table 1, entry 1). However, addition of TMEDA to generate **1b** in situ led to the formation of **2a** in a 62% yield (Table 1, entry 2), establishing the dramatic effect a donor has on this reaction, favouring the formation of a less aggregated and consequentially more kinetically active organosodium base. Importantly, the reaction needed to be carried out at −40 °C to avoid competing metalation of TMEDA. Increasing the denticity of the Lewis donor by using PMDETA furnished **2a** in a high yield (83%) (Table 1, entry 3), whereas the use of ethereal solvents as donors was detrimental to the overall reactivity.

**Table 1:** Optimisation of benzene carboxylation with various alkali metal bases.

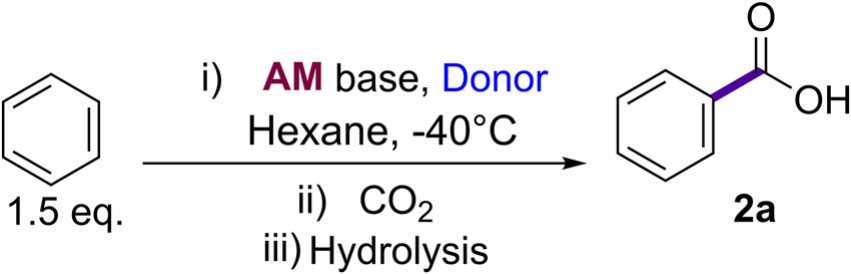			
Entry	AM Base	Donor	Yield (%)
1	NaNp^a)^	None	traces
2	NaNp	TMEDA	62
**3**	**NaNp**	**PMDETA**	**83**
4	LiNp	PMDETA	14
5	NaCH_2_SiMe_3_	PMDETA	21
6	NaTMP	PMDETA	traces
7	LiCKOR	PMDETA	56^b)^
8	LiNp + NaO*t*Bu	PMDETA	49
9	KNp^c)^	PMDETA	79(49)^d)^

Conditions: i) alkali metal base (1 mmol), Lewis base donor (1 mmol), benzene (1.5 mmol), hexane (5 mL), −40 °C, 4 h; ii) CO_2_ (1 atm); iii) hydrolysis with 1 M HCl. Yields determined by ^1^H NMR using internal standard of hexamethyl benzene (10 mol%).

^a)^ reaction carried out at room temperature;

^b)^ several products observed by ^1^H NMR.

^c)^ See Supporting Information for details.

^d)^ Reaction performed in the absence of PMDETA.

Interestingly, when comparing these optimised reaction conditions to other organometallic bases we found that in the presence of PMDETA the NaNp was remarkably superior to the congeneric lithium reagent LiNp which yielded **2a** in a poor 14% yield (Table 1, entry 4), this lowered Brønsted basicity of the alkyl lithium presumably arising from the less polar nature of the Li─C bond compared to the Na─C bond of alkyl sodiums. The identity of the alkyl group was also found to be important, as when the powerful alkyl sodium NaCH_2_SiMe_3_^[^ ^34^ ^]^ was used **2a** was obtained in a modest 21% yield (Table 1, entry 5). Sodium amide NaTMP (TMP = 2,2′,6,6′‐tetramethylpiperidide) also failed to metalate benzene under the conditions of the study, (Table 1, entry 6).^[^ ^54^ ^]^ Moreover, the classical Lochmann–Schlosser LICKOR superbase in the presence of PMDETA proved better but the yield was again diminished (56%; Table 1, entry 7) compared to our new base combination and no product was observed in hexane with the absence of PMDETA. Incomplete superbase metalations have previously been attributed to some side reactions such as multiple metalations on the benzene ring.^[^ ^55^ ^]^ Combining LiNp with NaO*t*Bu in situ also resulted in a lower yield of **2a** (49%) (Table 1, entry 8), thus highlighting the superior behaviour of the pure isolated alkyl sodium reagent for a more effective performance of the Na─H exchange process. Interestingly it should be noted that addition of LiO*t*Bu to a solution of NaNp/TMEDA in hexane did not have an inhibitory effect on the metalation of benzene. Finally, the potassium analogue (KNp) was tested (Table 1, entry 9) which in the presence of PMDETA formed **2a** in comparable yields (79%) to those observed for NaNp. Notably, in the absence of PMDETA the yield of **2a** decreased to 49%.

**Scheme 4:**
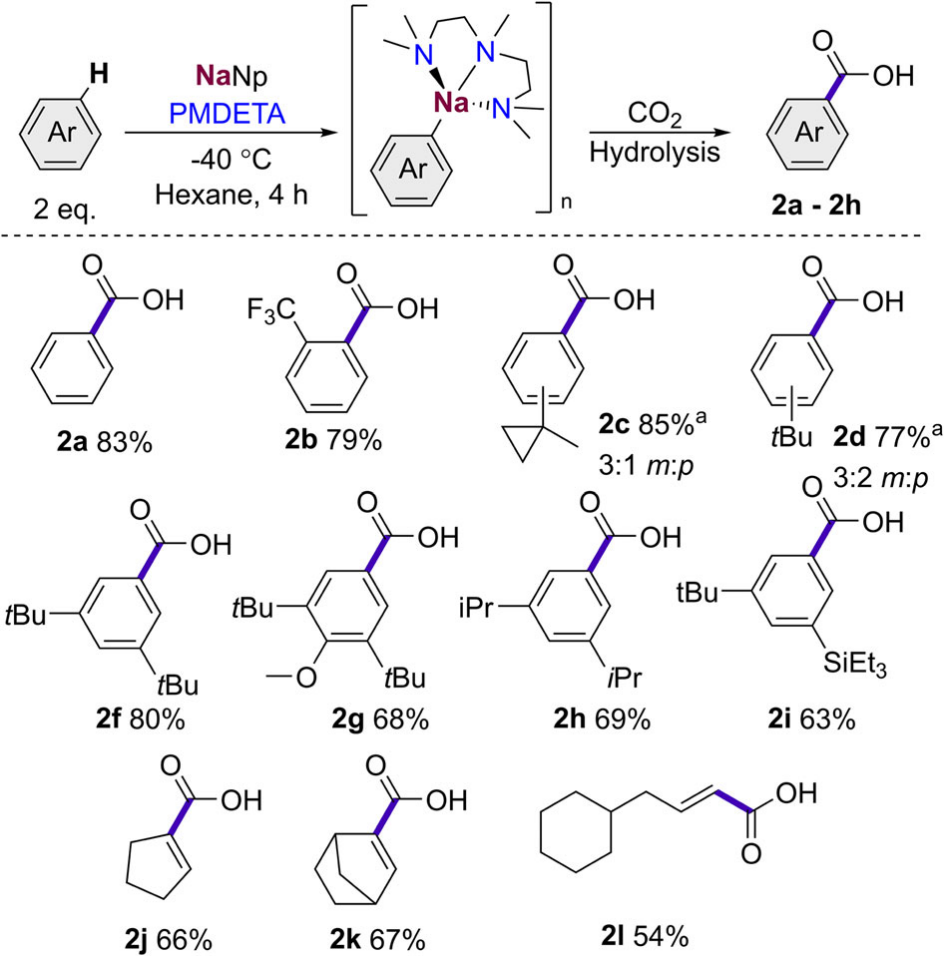
Metalation and subsequent CO_2_ quench of non‐activated substrates. Isolated yields. Conditions: i) NaNp (0.5 mmol), PMDETA (0.5 mmol), substrate (1.0 mmol), −40 °C, 4 h, hexane; ii) CO_2_ (1 atm), rt, 1 h; iii) hydrolysis by 1 M HCl. [a] Inseparable mixture of *meta* and *para* isomers.

Building on these findings, we next investigated the scope of our metalations to other non‐activated and alkyl‐substituted arenes (Scheme 4).

The presence of an acidifying trifluoromethyl group led to the formation of *ortho*‐substituted **2b** in a good yield of 79%, this result is in agreement with our previous studies where the metalation of this substrate at −78 °C with *n*‐butyl sodium was investigated.^[^ ^56^ ^]^ When introducing a bulky alkyl group onto the aryl ring without an available benzylic proton, such as in 1‐methylcyclopropyl or *tert*‐butyl, we found facile metalation under our conditions to form the corresponding carboxylic acids **2c** and **2d** after a CO_2_ quench and subsequent hydrolysis in excellent yields (85% and 77%). Interestingly, these bulky groups show a remote directing effect favouring the formation of a mixture of the *meta* and *para* metalation (in a 3:1 *m*:*p* ratio or 3:2 *m*:*p* ratio for **2c** and **2d** respectively). The good yields for **2c** and **2d** contrast with those reported by Schlosser using the LiCKOR superbase, showing these substrates could be carboxylated only in modest yields of 53% and 52%, respectively,^[^ ^55^ ^]^ whereas a low yield of 15% was achieved when using pentyl sodium for the metalation of *tert*‐butyl benzene at room temperature for 4 h.^[^ ^57^ ^]^ On the basis of NMR studies, here the authors tentatively propose that the electron rich alkyl groups significantly slow the metalation on the aryl rings due to a *σ‐* coupled deformation of the *π*‐ electrons in the rings.^[^ ^58^ ^]^ Thus, the high yields observed for these substrates when using neopentyl sodium as a base solvated by PMDETA further exemplifies the power of this highly basic combination.

**Scheme 5:**
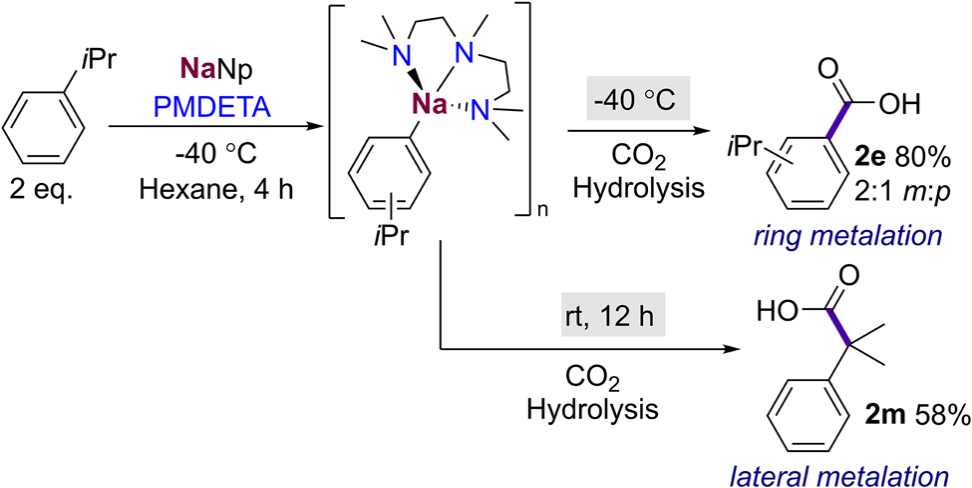
Metalation and subsequent CO_2_ quench of isopropylbenzene, showing divergent selectivity over time.

We found that introducing a substrate with a poorly accessible benzylic proton such as in isopropylbenzene gave solely aryl metalation, affording **2e** as a mixture of *meta* and *para* isomers in a good yield of 80% after 4 h at −40 °C (Scheme 5). However, when the reaction was allowed to warm to room temperature and left to stir overnight, there was full conversion of the aryl sodium to the benzylic species, which can be then intercepted with CO_2_ to give lateral metalation product **2m** in a 58% yield, showing the ability of the anion to migrate over time to the more stabilised position. These findings concur with previous studies by Crimmins, where addition of TMEDA and isopropylbenzene to a pentylsodium suspension in hexane resulted in the selective lateral metalation over a period of 24 h.^[^ ^59^ ^]^

Next, we sought to introduce multiple bulky functional groups onto our substrates to achieve more selective functionalisation. In this regard Schlosser has shown that LiTMP can direct the remote *meta*‐metalation of (2,6‐dichloro‐phenyl)triethylsilane.^[^ ^60^ ^]^ More recently Knochel has elegantly demonstrated the ability of 1,3‐bis(triethylsilyl)‐substituted arenes to undergo selective lithiation at their C5 position.^[^ ^61^ ^]^ Furthermore, Antonov has also shown, with the aid of computational studies, the effective utilisation of non‐covalent interactions for switching the regioselectivity of the lithiation of pyridine derivatives.^[^ ^62^ ^]^ We hypothesised that the incorporation of two bulky substituents *meta* to each should direct the selectivity on the metalation process due to the increase of steric congestion. We were pleased to find that 1,3‐ditertbutylbenzene is metalated in the 5‐ position to afford **2f** in an excellent yield of 80%. This approach was then extended to 2,6‐di‐*tert*‐butyl‐anisole, 1,3‐diisopropylbenzene and 3‐*tert*butylphenyltriethylsilane giving the relevant *meta‐*substituted carboxylic acids **2** **g**–**2i** in yields ranging from 63% to 69% (Scheme 4).

Early studies in the 1960′s have hinted at the potential of alkyl sodium bases to deprotonate cyclic olefins, although harsh conditions with long reaction times are required.^[^ ^63^ ^]^ With our optimised metalating conditions, we hoped to extend our scope of sodiated species from benzene derivatives to cyclic and more strained alkenes. Starting from the more acidic cyclopentene, we were pleased to observe excellent metalation and subsequent carboxylation under our standard conditions to give **2j** in a reasonable yield of 66%, with no sign of competing allylic metalation. However, adding one more carbon to the ring in the form of cyclohexene, led solely to allylic metalation. Unfortunately, we found a mixture of products formed in the CO_2_ quench, so a phenyl Weinreb amide quench was used, giving the corresponding ketone product **4ba** in a reasonable yield (58%) (see Supporting Information). Finally, we found that adding more strain to the cyclohexene ring in the form of norbornene helped to direct the metalation to the alkene, which could be quenched with CO_2_ to form **2k** in a good yield of 67%. Turning our attention to the less activated linear alkenes, metalation and subsequent functionalisation of allylcyclohexane can be accomplished furnishing 4‐cyclohexylbut‐2‐enoic acid (**2l**) in a reasonable yield of 54%.

**Figure 3:**
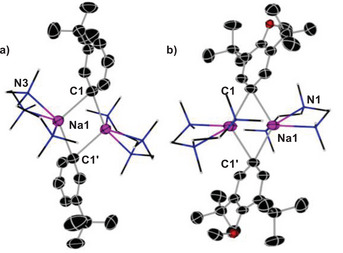
Molecular structure of **3a** a) and **3b** b) with 50% probability displacement ellipsoids. All H atoms have been omitted for clarity.

We next looked at the challenge of isolating and characterising possible sodiated intermediates from these reactions. Treating *tert‐*butylbenzene and 2,6‐di‐*tert*‐butyl‐anisole in hexane with equimolar amounts of NaNp and PMDETA at low temperature facilitated isolation of [{(PMDETA)Na(3‐*t*BuPh)}_2_] (**3a**) and [{(PMDETA)Na(1‐OMe‐2,3‐*t*Bu_2_‐C_6_H_2_)}_2_] (**3b**) as crystalline solids (see Supporting Information for details). X‐ray crystallographic studies established their molecular structures. Both adopt similar dimeric motifs, having a central four‐membered (NaC)_2_ ring, with aryl bridges between the Na atoms, which are further stabilised by binding to PMDETA (Figure 3). It should be noted that while made in situ in solution the metalation of *tert*‐butyl benzene formed a mixture of *meta* and *para* metalation, both aryl groups present in solid **3a** are *meta*‐metalated. They share the same structural motif as [{(PMDETA)NaPh}_2_] which was reported by Weiss and made via salt‐metathesis of PhLi with NaO*t*Bu.^[^ ^64^ ^]^

The Na─C bond distances in **3a** and **3b** [ranging from 2.6255(19) to 2.6659(13) Å] lie within the typical range of those previously reported for other aryl sodium complexes.^[^ ^46^, ^56^, ^65^ ^]^ Compounds **3a** and **3b** are not stable for long enough in deuterated benzene solutions to acquire meaningful DOSY NMR spectra, though for **3b** a diagnostic downfield signal at 196.5 ppm is observable in the ^13^C NMR spectrum for the NaC_Ar_.

**Scheme 6:**
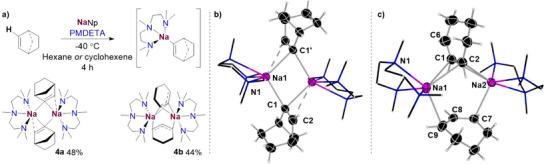
a) Metalation of non‐activated cyclic alkenes. b) Molecular structure of **4a** with 50% probability, displacement ellipsoids. H atoms shown as ball and stick model on norbornyl rings. c) Molecular structure of **4b** with 50% probability, displacement ellipsoids. H atoms shown as ball and stick model on cyclohexenyl rings.

Using a similar synthetic approach to that described for **3a** and **3b**, [{(PMDETA)Na(2‐norbornenyl)}_2_] (**4a**) and [{(PMDETA)Na(Cyclohexenyl)}_2_] (**4b**) were isolated as crystalline organometallic intermediates (prior to any onward bond‐forming reaction) resulting from sodiation of norbornene and cyclohexene in 48% and 44% yields, respectively (Scheme 6a). X‐ray crystallographic studies established **4a** exhibits a similar dimeric motif to those described for **3a** and **3b**, with the norbornenyl group bridging between the Na atoms via the C of the C─H bond that has been metalated [Na1─C1 = 2.6029(12) Å, Na1─C1’ = 2.6487(11) Å] (Scheme 6b). The Na atoms also engage in an additional, secondary electrostatic *π*‐interaction with the remaining vinylic CH unit [Na1─C2 = 2.9752(19) Å]. The vinylic character of **4a** is also apparent by the length of its C1─C2 bond [1.3054(23) Å] which is consistent with a localised C═C bond. As far as we can ascertain from a search of the Cambridge Structural Database, **4a** is the first example of a C2‐metalated norbornene complex to be structurally defined. Furthermore, it should also be noted its rare sodium vinylic constitution, for which there are hardly any precedents in the literature.^[^ ^66^ ^]^

In contrast, the **4b** dimer contains two cyclohexenyl bridges, both deprotonated at their allylic position, that coordinate in a non‐symmetric fashion to both {Na(PMDETA)} cations and adopt a perpendicular disposition with respect to each other (Scheme 6c).Thus, one allyl group binds in a η^2^‐fashion to both Na atoms [Na1–C1/C2 average distance, 2.7416 Å; Na2–C1/C2 average distance, 2.8793 Å]. The second cyclohexenyl binds to each sodium atom using only one of its terminal allylic carbons [Na1–C9, 2.6823(18) Å; Na2–C7, 2.6509(14) Å]. These values are in accordance with previously reported Na─C_allyl_ bond lengths.^[^ ^67^, ^68^, ^69^ ^]^ The allylic nature of the cyclohexenyl fragments was observed by examining the shortened average C─C bond lengths [C2–C1/C1–C6, 1.3851 Å], [C7–C8/C8–C9, 1.3821 Å]. The coordination of the cyclohexenyl groups in **4c** is significantly different to those recently reported by us for other PMDETA‐solvated sodium allyl complexes containing non‐cyclic allyl ligands which form monomeric motifs with the allyl group adopting η^3^‐coordination.^[^ ^67^ ^]^ A possible rationale for this unusual coordination preference can be the large steric constraints imposed by the cyclic structure of the cyclohexenyl groups (see Figure S68 in Supporting Information for a space filling model representation). As a consequence of this preferred structural arrangement the two Na atoms are significantly further apart from each other than those in **4a** [4.4930(6) versus 3.2117(7) Å].

Attempts to assess aggregation of **4a** and **4b** in deuterated cyclohexane were also unfruitful due to their limited stability in solution which also preluded the acquisition of their ^13^C NMR spectra (see Supporting Information for details).

**Scheme 7:**
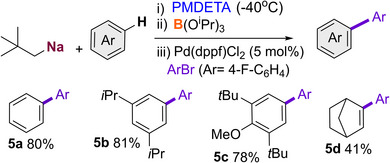
Pd mediated cross‐coupling of sodium borates formed via neopentyl sodiation. Conditions i) NaNp (1 mmol), PMDETA (1 mmol) substrate (1.5 mmol) −40 °C, 4 h, hexane; ii) B(O^i^Pr)_3_ (1 mmol) rt 16 h; iii) Pd(dppf)Cl_2_ (5 mol%), 1‐bromo‐4‐fluorobenzene (0.8 mmol), water (0.2 mL), 100 °C, 6 h, toluene.

Our previous studies have shown that aryl sodium boronates can be excellent precursors in Suzuki–Miyaura couplings.^[^ ^54^ ^]^ Using benzene, 2,6‐di‐tert‐butyl‐anisole, 1,3‐diisopropylbenzene and norbornene as model substates we were delighted to find that the metalation of these substrates with NaNp/PMDETA can be coupled in a sequential one‐pot approach with a transborylation step using B(O*i*Pr)_3_ and a palladium catalysed Suzuki–Miyaura coupling using 1‐bromo‐4‐fluorobenzene and 5 mol% of Pd(dppf)Cl_2_, to furnish **5a**‐**5d** (Scheme 7). Expanding further the synthetic potential of these sodium‐mediated transformations, bis(aryls) **5a‐c** were obtained in excellent yields, ranging from 78% to 81%; though in the case of cyclic alkene norbornene, **5d**, the yield dropped to 41%.

## Conclusions

Using a salt‐metathesis approach we have isolated and characterised solvent‐free hydrocarbon soluble neopentyl sodium, a reagent that has shown hints of promise in polar organometallic chemistry but whose constitution has remained elusive for decades. Combining spectroscopic and crystallographic studies with computational studies, valuable insight into the nature of bonding present in this compound and its stability have been gained. Unlike related alkylsodium complexes, NaNp exhibits a rare discrete tetrameric structure that displays high solubility in hydrocarbon solvents, a benefit likely to open up its utilisation in synthetic organic chemistry.

As an example towards such utilisation, here when we combined NaNp with PMDETA remote metalation of non‐activated C(*sp* ^2^) bonds in aryl and olefin substrates was achieved, whereas these substrates cannot be metalated by conventional s‐block metal bases such as lithium amides or alkyllithiums. This work could also stimulate development of more organometallic reagents of this environmentally benign, earth abundant metal.

## Conflict of Interests

The authors declare no conflict of interest.

## Supporting information



Supporting Information

Supporting Information

## Data Availability

The data that support the findings of this study are available in the Supporting Information of this article.
