# Supplementary material for: Synthesis and Structure of Neopentyl Sodium: A Hydrocarbon‐Soluble Reagent for Controlled Sodiation of Non‐Activated Substrates
Source: Angew Chem Int Ed Engl. 2025 Jul 29;64(37):e202511492. doi: 10.1002/anie.202511492 (PMC12416459; doi:10.1002/anie.202511492)
Supplement: Supplementary file 1 — Supporting Information [file ANIE-64-e202511492-s001.pdf]

# Synthesis and Structure of Neopentyl Sodium: A Hydrocarbon-Soluble Reagent for Controlled Sodiation of Non-activated Substrates

David E. Anderson, Lorraine A. Malaspina, Simon Grabowsky and Eva Hevia*

# Contents

**Synthesis and Structure of Neopentyl Sodium: A Hydrocarbon-Soluble Reagent for Controlled Sodiation of Non-activated Substrates** ........ 1

**General Methods** ........ 3

**Synthesis of Organometallic Intermediates** ........ 4

**Organometallic Starting Materials** ........ 4

**Synthesis of Neopentyl Lithium** ........ 4

**Synthesis of Neopentyl Sodium** ........ 5

**Synthesis of Neopentyl Potassium** ........ 5

**Synthesis of *n*BuNa and $NaCH_2SiMe_3$** ........ 6

**Synthesis of Crystallised Organometallic Complexes** ........ 6

**Synthesis of [$\{NaCH_2$*t*Bu$\}_4$] (1a)** ........ 6

**Alternative synthesis of 1a** ........ 8

**Synthesis of [{(PMDETA)Na(3-*t*BuPh)$\}_2$] (1b)** ........ 9

**Synthesis of [{(PMDETA)Na(3-*t*BuPh)$\}_2$] (3a)** ........ 10

**Synthesis of [{(PMDETA)Na(1-OMe-2,3-*t*$Bu_2$-$C_6H_2$)$\}_2$] (3b)** ........ 12

**Synthesis of [{(PMDETA)Na(2-norbonenyl)$\}_2$] (4a)** ........ 14

**Synthesis [{(PMDETA)Na(Cyclohexenyl)$\}_2$] (4b)** ........ 16

**$^1$H-DOSY NMR Spectroscopic Analysis** ........ 24

**NMR Monitoring of Addition of PMDETA to 1a at Room Temperature** ........ 26

**Synthesis of Organic Products** ........ 27

**Sodium Halogen Exchange of 1-Bromonaphthalene** ........ 27

**General Procedure for Carboxylations (General procedure A)** ........ 28

**General Procedure for Cross couplings (General procedure B)** ........ 34

**NMR spectra of isolated products** ........ 37

**Sodium neo-pentenyl, theoretical analysis** ........ 58

**X-ray Crystallographic Details** ........ 65

**Space filling model of 4b** ........ 72

**References** ........ 73

# General Methods

All procedures were conducted using standard Schlenk line and glove box techniques under an inert atmosphere of argon. Hexane was degassed, purified and collected via an MBraun SPS 5 and stored over 4 Å molecular sieves for at least 24 hours prior to use. THF was dried by heating to reflux over sodium-wire/benzophenone ketyl radical and stored over 4 Å molecular sieves for 24 hours prior to use. Deuterated solvents ($C_6D_6$ and $C_6D_{12}$) were purchased from VWR, dried over NaK alloy for 16 hours and then cycled through three rounds of degassing by employing a freeze-pump-thaw method. The deuterated solvents were then collected via vacuum transfer and stored under argon atmosphere over 4 Å molecular sieves. All remaining substrates employed in this study are commercially available and were used as received (solids) or degassed by freeze-pump-thaw and stored over molecular sieves (liquids). The polydentate amines used in this study were dried over calcium hydride, distilled under reduced pressure and store over molecular sieves prior their use.

NMR spectra were recorded on Bruker spectrometers operating at either 300 or 400 MHz. 1H NMR spectra: 300.1 MHz, 400.1 MHz 13C NMR spectra: 75.5 MHz, 101 MHz. Spectra were analysed using MestReNova software and referenced internally to the corresponding residual protium solvent peaks. 1H DOSY NMR spectra were analysed using TopSpin software.

Purification of the final organic products were performed by column chromatography on silica gel using a CombiFlash®Rf system (Teledyne ISCO), with RediSep® Silver Normal-phase Silica Flash Columns as stationary phase and mixtures of hexane and ethyl acetate as mobile phase.

Elemental analyses (C, H and N) were conducted with a Flash 2000 Organic Elemental Analyser (Thermo Scientific). Samples were prepared in the glovebox under argon atmosphere and sealed in an air-tight container prior to analyses. All results were obtained by the Analytical Research and Services Schürch Group of the University of Bern. Samples were weighed on a Mettler Toledo balance with ±2 µg resolution and sample weights from 1-3 mg were used. For calibration, cysteine was used as a reference material. The presented values are the average of determinations in triplicate to ensure consistency.

HRMS was analysed by DIP-EI-MS (direct insertion probe) and all results were obtained by the Analytical Research and Services Schürch Group of the University of Bern.

# Synthesis of Organometallic Intermediates

## Organometallic Starting Materials

### Synthesis of Neopentyl Lithium

In an argon-filled J-Youngs ampoule, finely divided lithium metal (2.80 g, 400 mmol) was suspended in 75.0 mL of dry hexane. Neopentyl chloride (12.3 mL, 100 mmol) was slowly added, and the solution was heated at 65 °C for 1 week. The dark purple suspension was cooled to ambient temperature and filtered over celite using a glass-tapped filter frit and washed with 3 x 15 mL aliquots of fresh hexane. The filtrate was stored overnight at -40 °C, affording neopentyl lithium as a white crystalline solid which was then isolated by cannula filtration and subsequently washed with 3 x 15 mL of cold pentane. The solid was dried under vacuum and stored in a glovebox for further use. Typical yield = 4.29 g, 55 mmol, 55 %.

**$^1$H NMR** (300 MHz, $C_6H_{12}$) δ 1.05 (s, 11H), -0.61 (s, 2H).

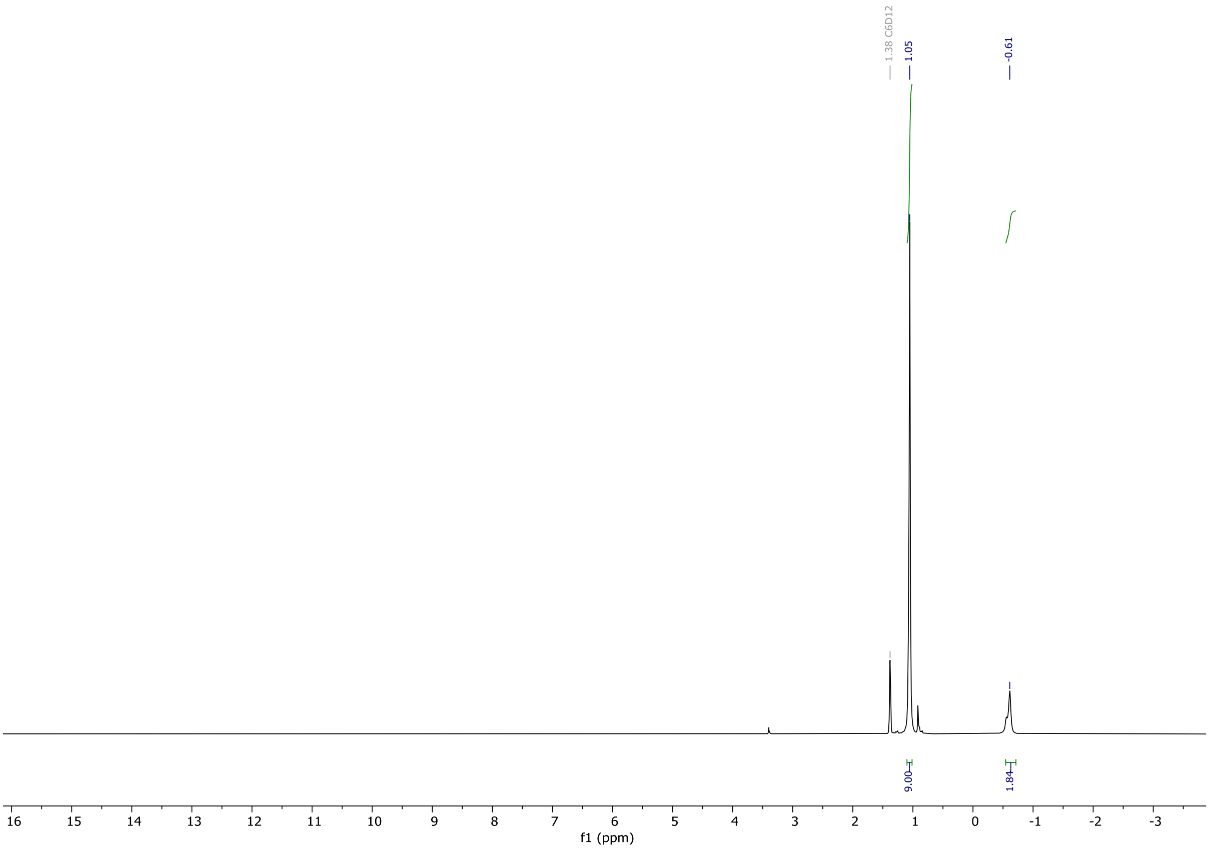


*Figure S1 $^1$H NMR of **LiNp** in $C_6D_{12}$.*

## Synthesis of Neopentyl Sodium

In an argon-filled Schlenk flask, 50.0 mL of dry hexane was added to 3.84 g (40 mmol) of NaO*t*Bu and 3.12 g (40 mmol) of $LiCH_2$*t*Bu at 0 °C affording a fine, white suspension which was stirred for 1 h. The suspension was warmed to ambient temperature and stirred overnight. The resulting suspension was cooled to -30 °C and isolation of the white precipitate ($NaCH_2CMe_3$) was achieved by gravity filtration using a glass-tapped filter frit. The filter cake was washed with 3 x 15 mL aliquots of fresh hexane until the liquors ran completely clear. The solid was dried under vacuum and stored in the glovebox for further use. Typical yield = 1.94 g, 21 mmol, 53 %.

## Synthesis of Neopentyl Potassium

Synthesis of $KCH_2CMe_3$ was performed in an analogous manner to $NaCH_2CMe_3$ (see above) on a 10 mmol scale, using $LiCH_2$*t*Bu and KOtBu. Yield = 240 mg, 2.18 mmol, 22%.

**$^1$H NMR** (300 MHz, $C_6H_{12}$) δ 1.01 (s, 9H), -0.88 (s, 2H). NMR consistent with literature reports.[1]

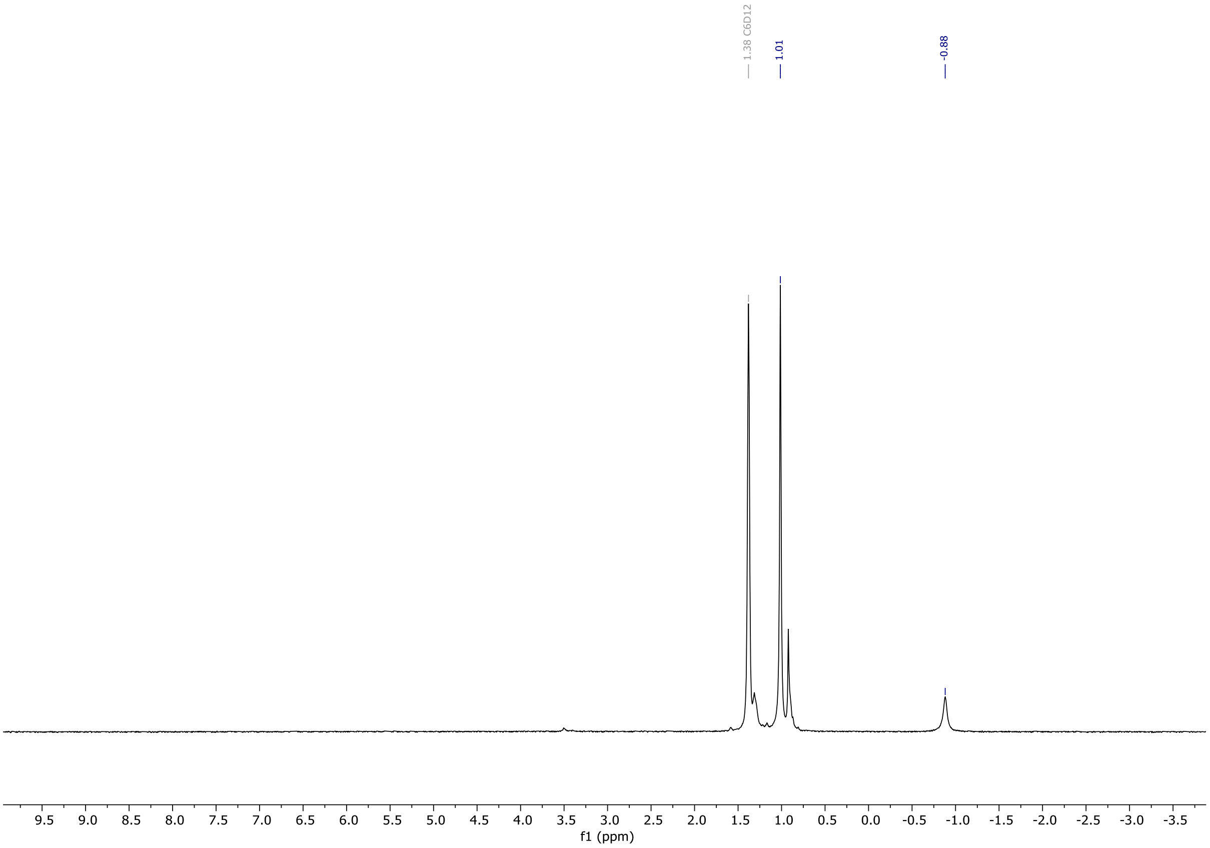


*Figure S2 $^1$H NMR of **KNp** in $C_6D_{12}$.*

## Synthesis of *n*BuNa and $NaCH_2SiMe_3$

*n*BuNa and $NaCH_2SiMe_3$ prepared according to literature procedure.[2]

# Synthesis of Crystallised Organometallic Complexes

## Synthesis of [{$NaCH_2$*t*Bu}$_4$] (1a)

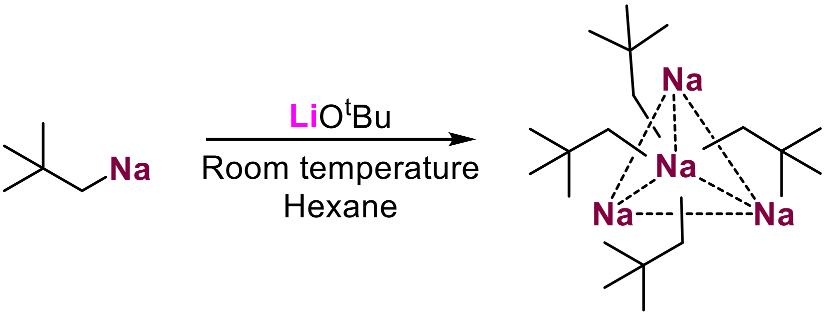


In a glovebox, $NaCH_2$*t*Bu (9.4 mg, 0.1 mmol) LiO*t*Bu (8.0 mg, 0.1 mmol) were dissolved in hexane (3 mL) in a 2-dram vial. Slow evaporation at room temperature led to the formation of air sensitive X-ray quality colourless crystals. (7.6 mg, 0.08 mmol, 81%)

**$^1$H NMR** (300 MHz, $C_6D_{12}$) δ 1.02 (s, 9H), -0.71 (s, 2H).

**$^{13}$C NMR** (75 MHz, $C_6D_{12}$) δ 37.8 (C-($\underline{C}H_3)_3$), 36.1 ($\underline{C}H_2$-Na), 34.1 ($\underline{C}$-($CH_3)_3$).

**Elemental analysis:** Calculated for $C_5H_{11}Na$: C, 63.66; H, 11.96; N, 0.00, found: C, 63.80; H, 11.78; N, 0.00.

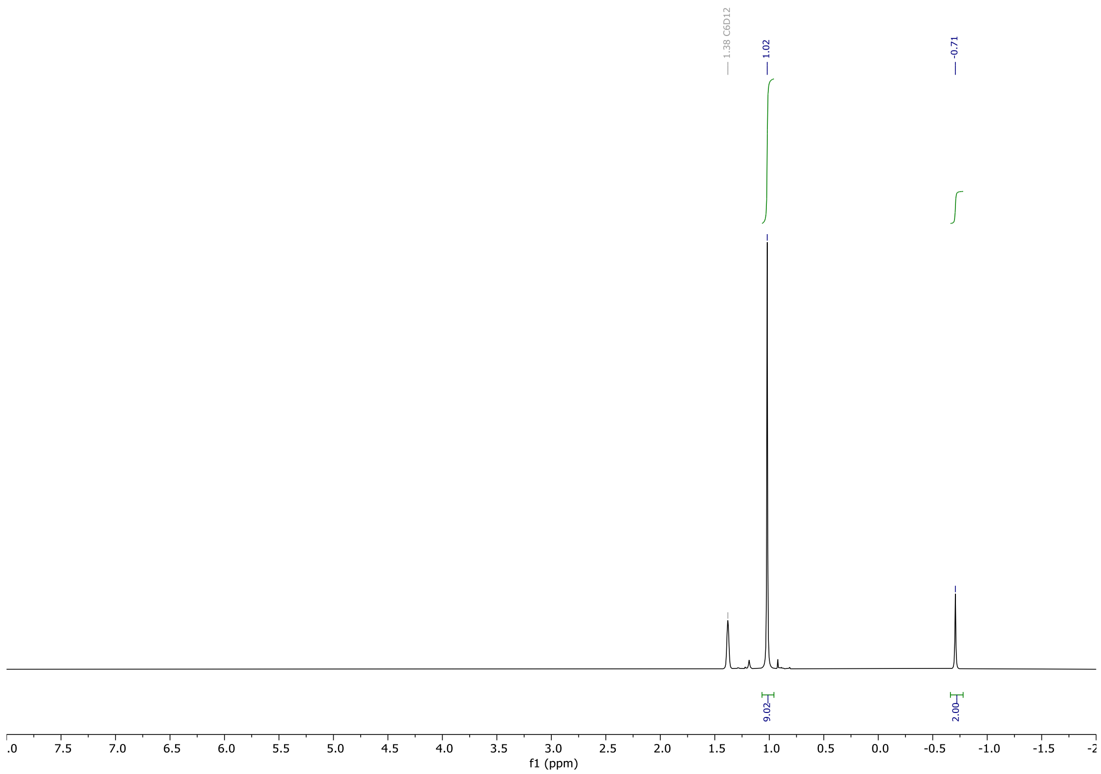


*Figure S3 $^1$H NMR of **1a** in $C_6D_{12}$.*

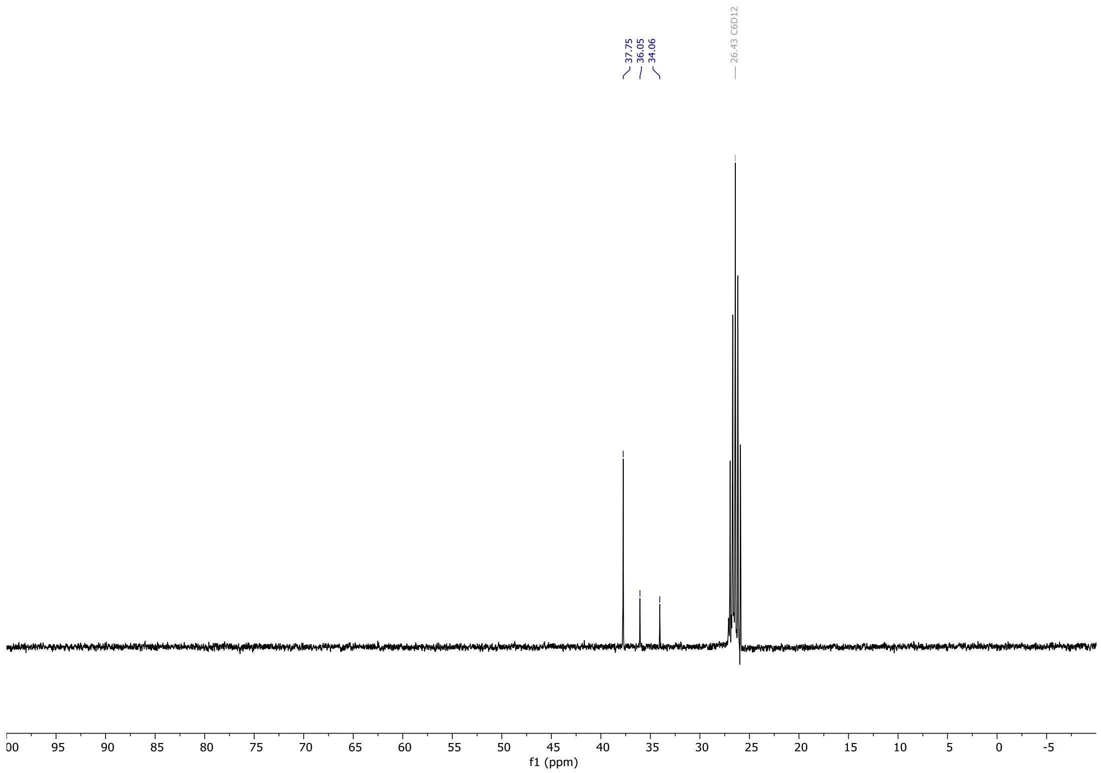


*Figure S4 $^{13}$C NMR of **1a** in $C_6D_{12}$.*

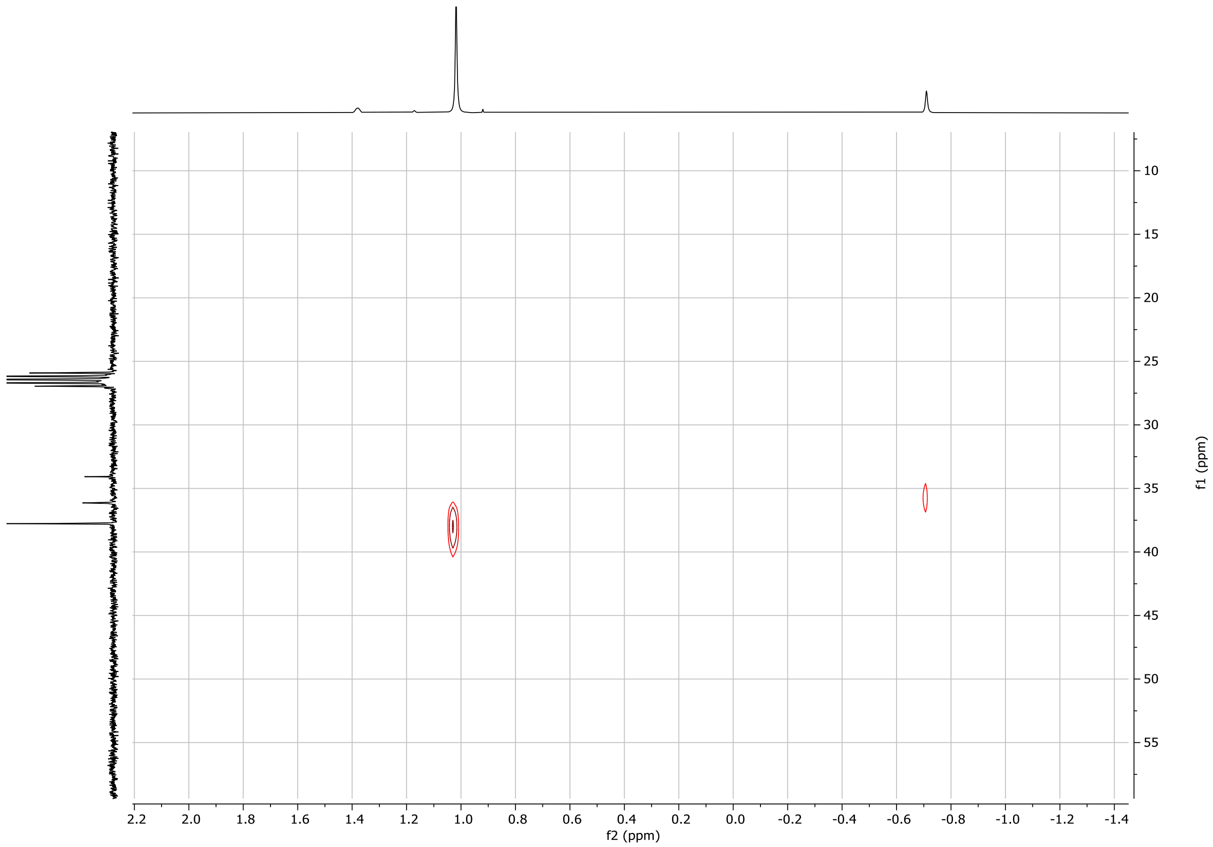


*Figure S5 $^{13}C/^{1}H$-HSQC of **1a** in $C_6D_{12}$.*

## Alternative synthesis of 1a

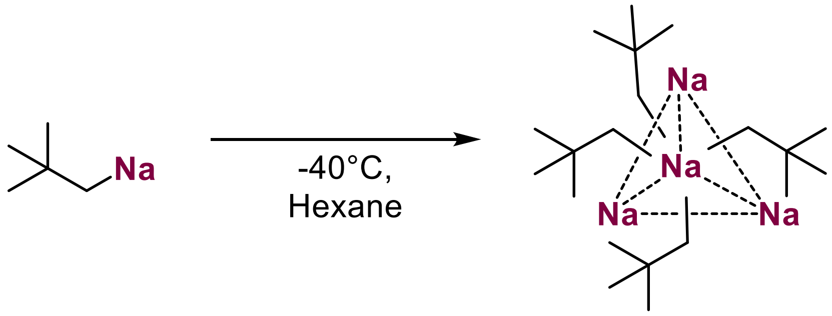


In a glovebox, $NaCH_2$*t*Bu (18.8 mg, 0.2 mmol) was dissolved in hexane (3 mL) in a 2-dram vial. Storing the solution in the glovebox freezer at -30°C led to the formation of a large crop of air sensitive colourless needle like crystals. (16 mg, 0.17 mmol, 85%). NMR and elemental analysis of the crystals confirmed the same product **1a** was formed.

## Synthesis of [{(PMDETA)Na(3-*t*BuPh)}$_2$] (1b)

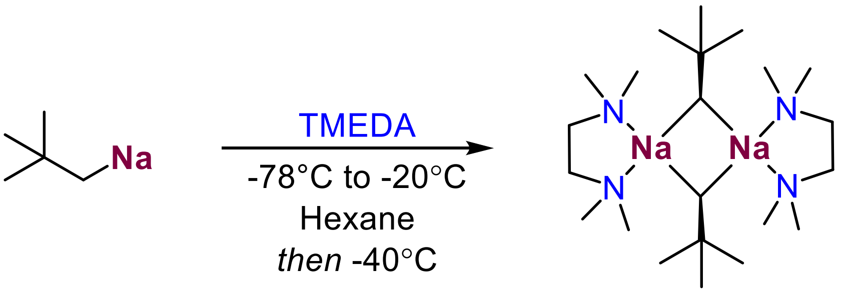


In an argon-flushed Schlenk flask, $NaCH_2$*t*Bu (1 mmol, 94 mg) was suspended in hexanes (5 mL) at -80°C, to which TMEDA (1 mmol, 0.150 mL) was added via syringe. The resulting suspension was slowly warmed to -20°C, where the suspension had turned to a clear solution. The solution was stored overnight at -40°C whereupon colourless needle like crystals had formed (suitable for X-Ray diffraction). High temperature sensitivity of the crystals (melting and decomposition above -30 °C) precluded isolation and yield determination.

**$^1$H NMR** (400 MHz, $C_6D_{12}$) δ 2.31 (s, 4H), 2.23 – 2.18 (m, 12H), 1.02 (s, 9H), -0.73 (s, 2H).

Attempts to acquire adequate **$C^{13}$ NMR** data were unsuccessful, showing only signals relating to neopentane and metalated TMEDA

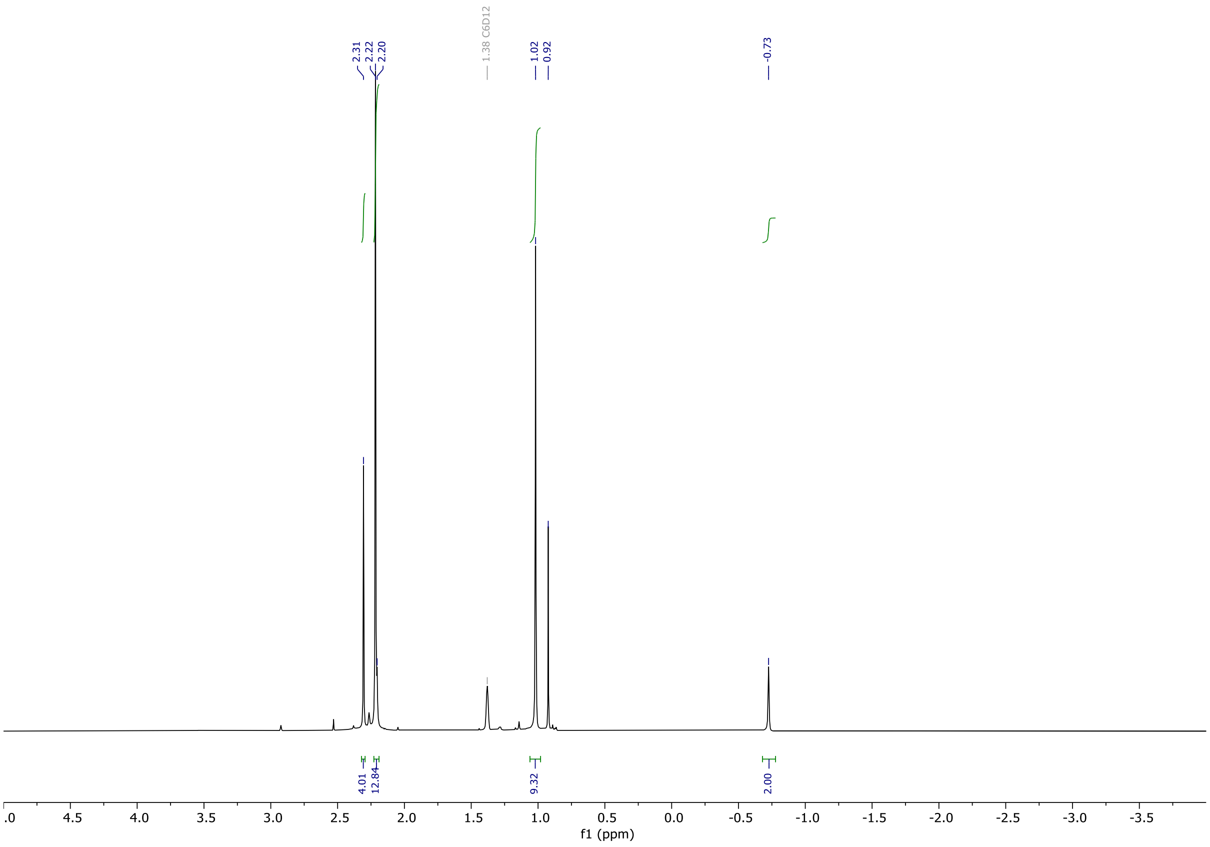


*Figure S6 $^1$H NMR of **1b** in $C_6D_{12}$.*

Despite several attempts, reliable elemental analysis was unable to be performed on the complex due to its high instability.

## Synthesis of [{(PMDETA)Na(3-*t*BuPh)}$_2$] (3a)

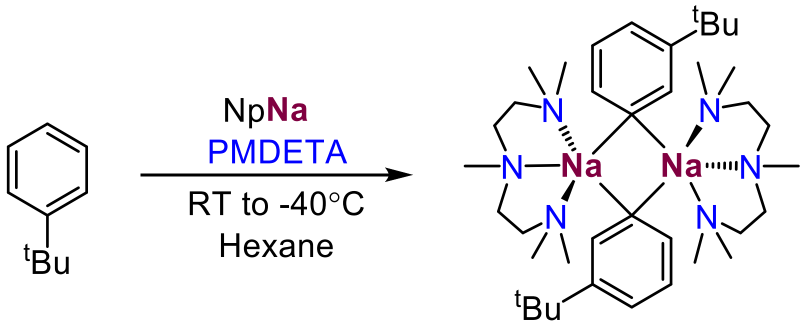


In an argon-flushed Schlenk flask, $NaCH_2CMe_3$ (1 mmol, 94 mg) was suspended in hexanes (5 mL) at room temperature, to which PMDETA (1 mmol, 0.210 mL) and tert-butylbenzene (2 mmol, 0.310 mL) were added via syringe. The resulting red solution was transferred to a freezer at -40°C and stored for 1 week, after which time, temperature sensitive colourless crystals of **3a** had formed (suitable for X-Ray diffraction). The crystals were isolated by canula filtration, washed with cold pentane and dried in vacuo (51 mg, 0.13 mmol, 13%)

**$^1$H NMR** (300 MHz, $C_6D_6$) δ 8.44 (d, *J* = 2.5 Hz, 1H), 8.19 (d, *J* = 6.3 Hz, 1H), 7.38 (t, *J* = 7.0 Hz, 1H), 7.30 – 7.20 (m, 1H), 2.12 (s, 12H), 2.04 (s, 8H), 1.67 (s, 3H), 1.60 (s, 9H).

**$^{13}$C NMR** (75 MHz, $C_6D_6$) δ 144.8 ($C_{Ar}$), 142.1 ($C_{Ar}$), 141.2 ($C_{Ar}$), 124.7 ($C_{Ar}$), 119.7 ($C_{Ar}$), 57.6 ($\underline{C}H_2$ PMDETA), 55.6 ($\underline{C}H_2$ PMDETA), 45.6 ($\underline{C}H_3$ PMDETA), 42.5 ($\underline{C}H_3$ PMDETA), 32.6 ($\underline{C}(CH_3)_3$), 31.5 ($C(\underline{C}H_3)_3$). ($\underline{C}$-Na signal not observed)

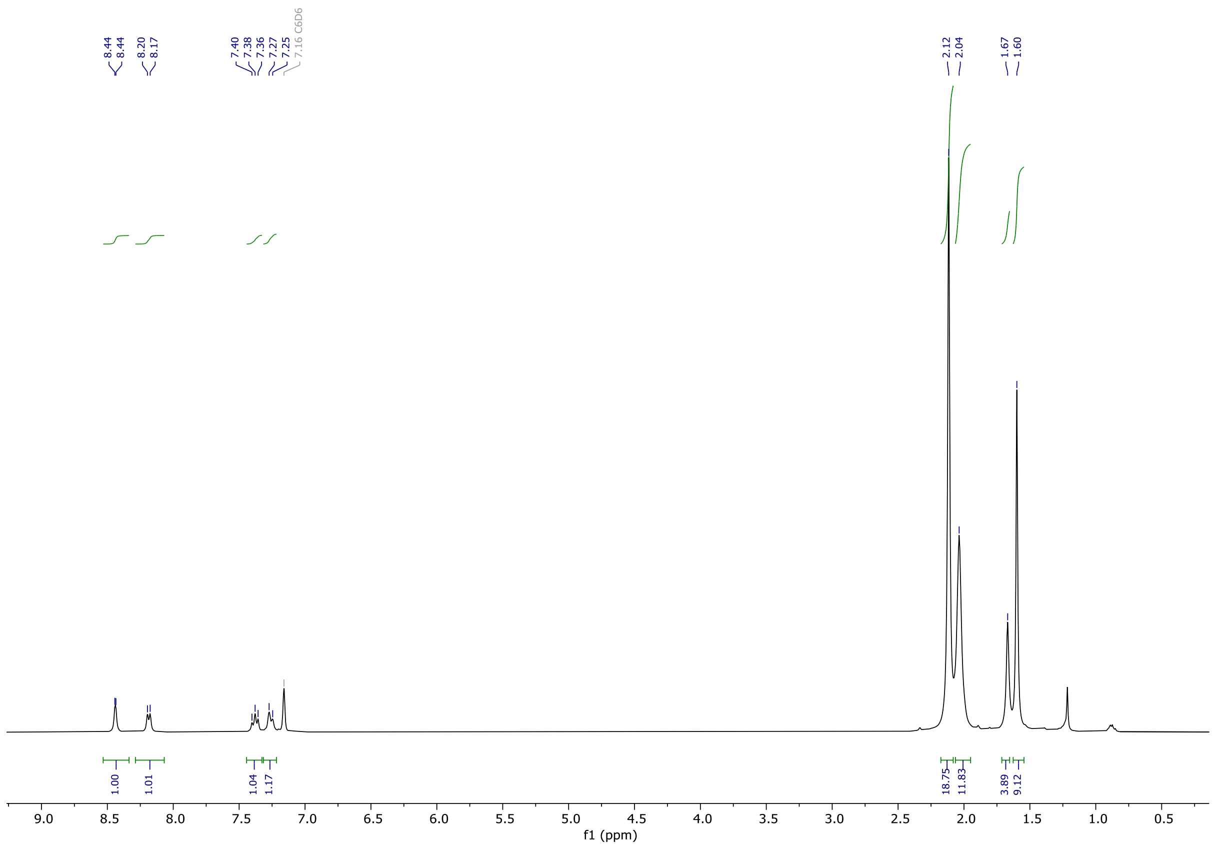


*Figure S7 $^{1}H$ NMR of **3a** in $C_6D_6$ (excess PMDETA added for solubility).*

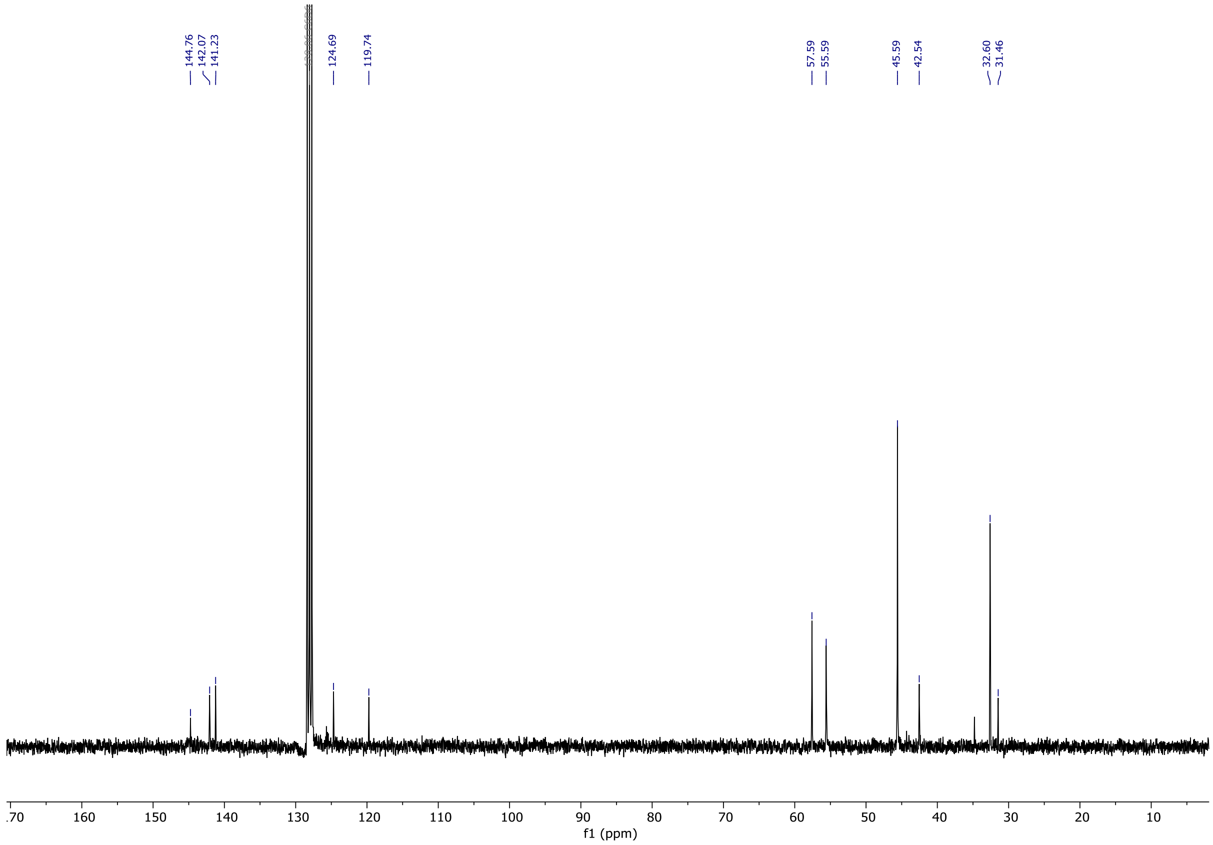


*Figure S8 $^{13}C$ NMR of **3a** in $C_6D_6$.*

Despite several attempts, reliable elemental analysis was unable to be performed on the complex due to the highly sensitive nature of the crystals.

## Synthesis of [{(PMDETA)Na(1-OMe-2,3-*t*Bu$_2$-C$_6$H$_2$)}$_2$] (3b)

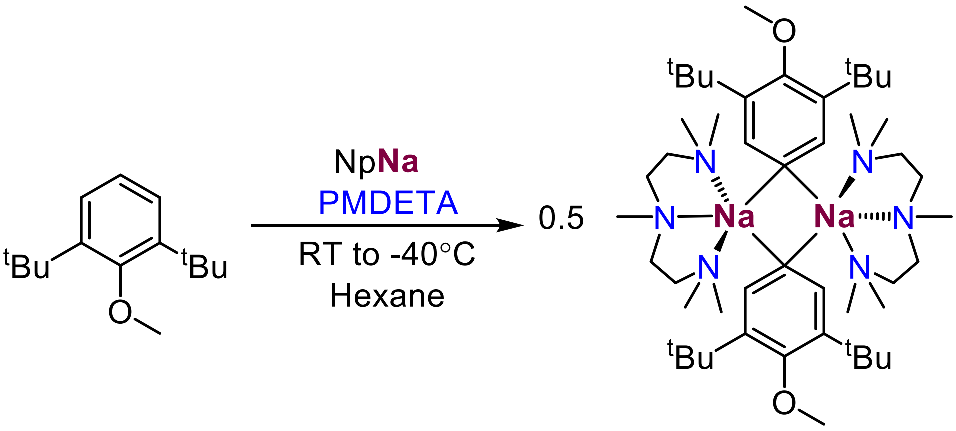


In an argon-flushed Schlenk flask, NaCH$_2$CMe$_3$ (1 mmol, 94 mg) was suspended in hexanes (5 mL) at -40°C, to which PMDETA (1 mmol, 0.210 mL) and 1,3-di-tert-butyl-2-methoxybenzene (2 mmol, 0.490 mL) were added via syringe. The white suspension was stirred at -40°C for 4 hours and transferred to a freezer where it was stored for 3 days at -40°C after which time, temperature sensitive colourless block like crystals had formed (suitable for X-Ray diffraction). The crystals were isolated through canula filtration, subsequent washing with cold pentane followed by drying in vacuo afforded **3b** as colourless crystals (41 mg, 0.10 mmol, 10%).

**$^1$H NMR** (400 MHz, C$_6$D$_6$) δ 7.23 (s, 2H), 3.38 (s, 3H), 2.10 (s, 10H), 2.05 (d, *J* = 12.7 Hz, 10H), 1.73 (s, 3H), 1.44 (s, 18H).

**$^{13}$C NMR** (101 MHz, C$_6$D$_6$) δ 196.5 (Na-$\underline{C}_{Ar}$) , 160.0 ($\underline{C}_{Ar}$), 143.7 ($\underline{C}_{Ar}$), 126.9 ($\underline{C}_{Ar}$), 64.1 (O$\underline{C}$H$_3$), 57.6 ($\underline{C}$H$_2$ PMDETA) , 55.7 ($\underline{C}$H$_2$ PMDETA), 45.6 ($\underline{C}$H$_3$ PMDETA), 42.5 ($\underline{C}$H$_3$ PMDETA), 35.9 ($\underline{C}$(CH$_3$)$_3$), 32.4 (C($\underline{C}$H$_3$)$_3$).

**Elemental analysis**: Calculated for C$_{24}$H$_{46}$NaON$_3$ : C, 69.35; H, 11.16; N, 10.11, found: C, 69.73; H, 11.16; N, 10.42.

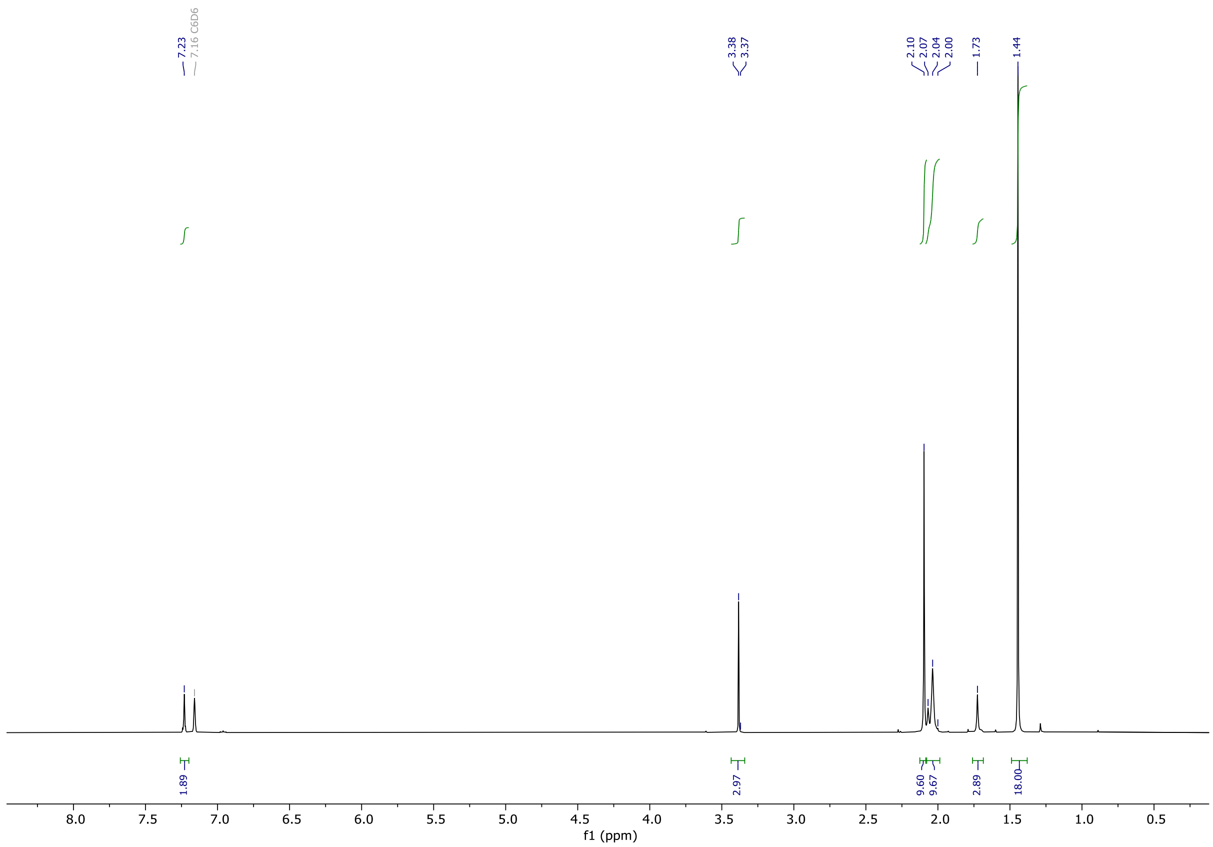


*Figure S9 ¹H NMR of **3b** in $C_6D_6$.*

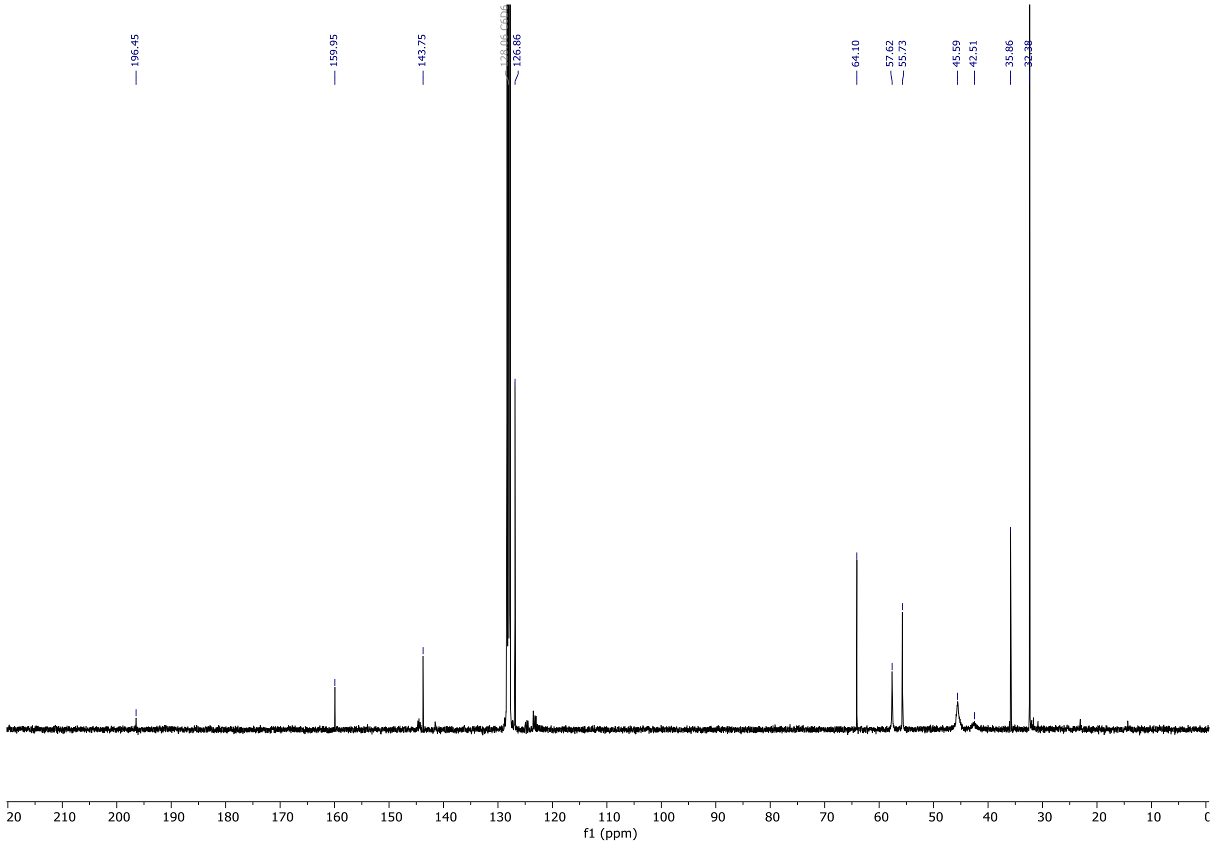


*Figure S10 ¹³C NMR of **3b** in $C_6D_6$.*

## Synthesis of [{(PMDETA)Na(2-norbonenyl)}$_2$] (4a)

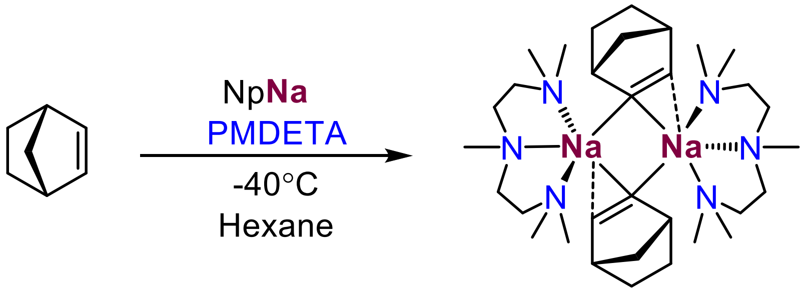


In an argon-flushed Schlenk flask, $NaCH_2CMe_3$ (0.5 mmol, 47 mg) was suspended in hexanes (3 mL) at -40°C, to which PMDETA (0.5 mmol, 0.105 mL) and norbornene (1 mmol, 0.94 mg) were added. The resulting pale-yellow suspension was stirred at -40°C for 4 hours. The suspension was slowly warmed to ~ 0°C until all solids had dissolved and quickly cooled back to -40 °C and stored for 2 days at that temperature, after which time, large colourless crystals had formed (suitable for X-Ray diffraction). The crystals were isolated by canula filtration, washed with cold pentane (3 x 5 mL) and dried under vacuum before storage in a glovebox freezer at -30 °C. (66 mg, 0.24 mmol, 48%).

**$^1$H NMR** (300 MHz, $C_6D_{12}$) δ 6.05 (d, $J$ = 2.2 Hz, 1H), 3.00 – 2.90 (m, 1H), 2.55 (s, 1H), 2.45 – 2.09 (m, 23H), 1.47 – 1.28 (m, 2H) (overlapping with solvent signal), 0.93 (q, $J$ = 2.2 Hz, 2H), 0.77 (t, $J$ = 9.3 Hz, 1H), 0.63 (t, $J$ = 9.4 Hz, 1H).

(Poor stability of sodiated norbornene in solution led to significant decomposition to norbornene and other unidentified products as seen by $^1$H NMR after 10 minutes. After longer times full formation of norbornene was observed.)

**Elemental analysis**: Calculated for $C_{16}H_{32}NaN_3$ : C, 66.40; H, 11.14; N, 14.52, found: C, 66.19; H, 11.18; N, 15.01.

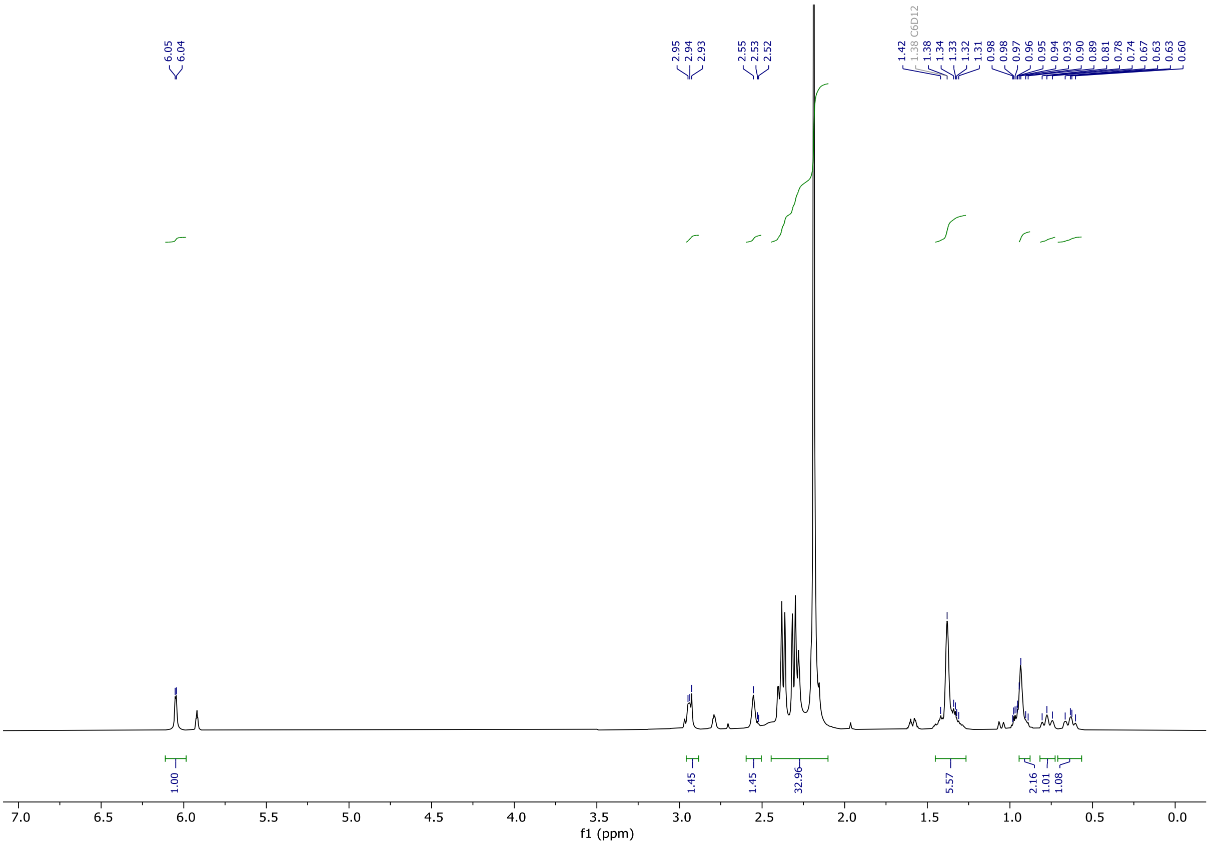


*Figure S11 $^{1}H$ NMR of **4a** in $C_6D_{12}$.*

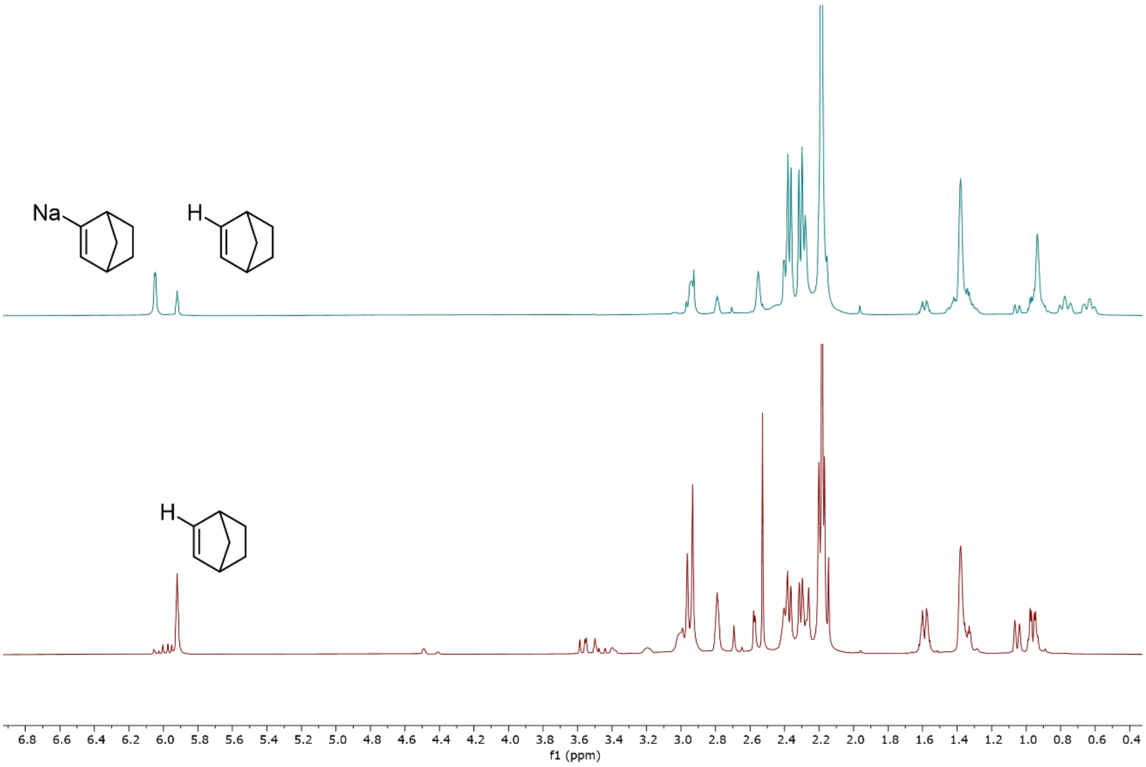


*Figure S12 Stacked $^{1}H$ NMR spectra of **4a** in $C_6D_{12}$ at different times showing increase in norbornene formation over time, Green – 15 minutes, Red – 1 hour.*

Attempts to acquire adequate **$C^{13}$ NMR** data were unsuccessful due to the observed poor stability of the complex in solution

## Synthesis [{(PMDETA)Na(Cyclohexenyl)}$_2$] (4b)

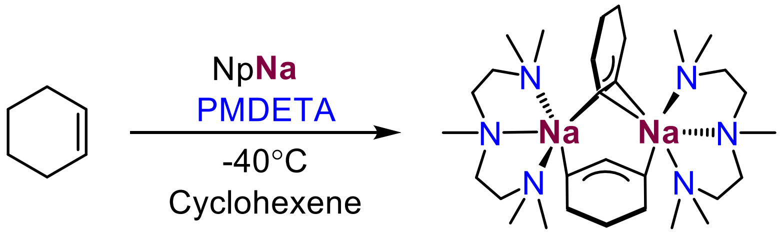


In an argon-flushed Schlenk flask, $NaCH_2CMe_3$ (0.5 mmol, 47 mg) was suspended in cyclohexene (3 mL) at -40°C, to which PMDETA (0.5 mmol, 0.105 mL) was added via syringe. The resulting bright yellow suspension was stirred at -40°C for 4 hours. The suspension was slowly warmed to ~ 0°C until all solids had dissolved and quickly cooled down to -30°C and stored for 2 days at that temperature, after which time, large yellow crystals had formed (suitable for X-Ray diffraction). The crystals were isolated by canula filtration, washed with cold pentane (3 x 5 mL) and dried under vacuum before storage in a glovebox freezer at -40°C. (61 mg, 0.22 mmol, 44%).

Poor solubility combined with rapid decomposition of crystals in solution lead to difficulty with full assignment of NMR. Full decomposition to cyclohexene and various unidentified products within 15 minutes in $C_6D_6$ observed by $^1H$ and $^{13}C$ NMR, ~50% decomposition within 15 minutes in $C_6D_{12}$.

**$^1$H NMR** (400 MHz, $C_6D_{12}$) δ 6.40 (t, *J* = 6.6 Hz, 2H), 2.46 – 2.42 (m, 3H) (resonance overlapping with PMDETA signal), 2.41 – 2.37 (m, 4H), 2.33 – 2.28 (m, 4H), 2.25 (s, 3H), 2.20 (s, 12H), 1.74 (p, *J* = 5.7 Hz, 4H).

**Elemental analysis**: Calculated for $C_{15}H_{32}NaN_3$ : C, 64.94; H, 11.63; N, 15.15, found: C, 65.78; H, 12.17; N, 15.73.

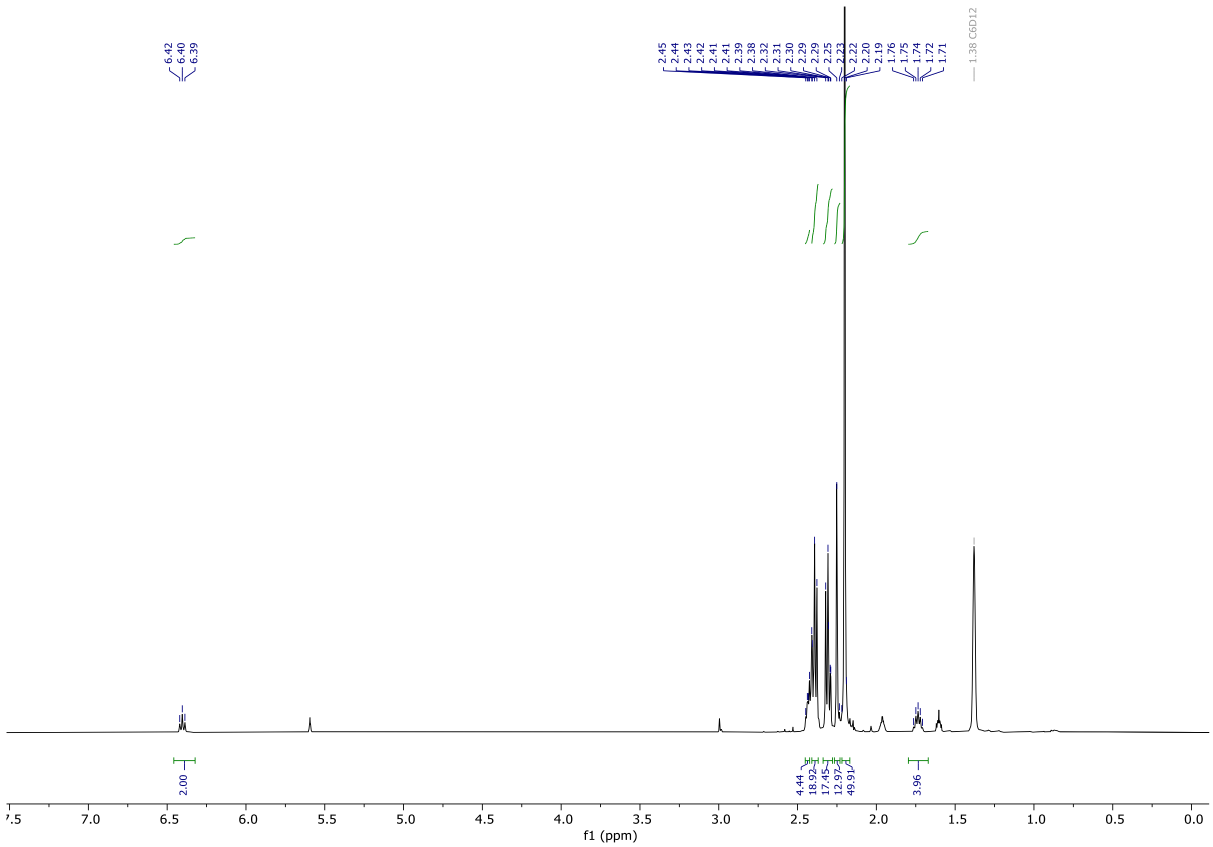

Figure S13 [1]H NMR of **4b** in $C_6D_{12}$ after 15 minutes.

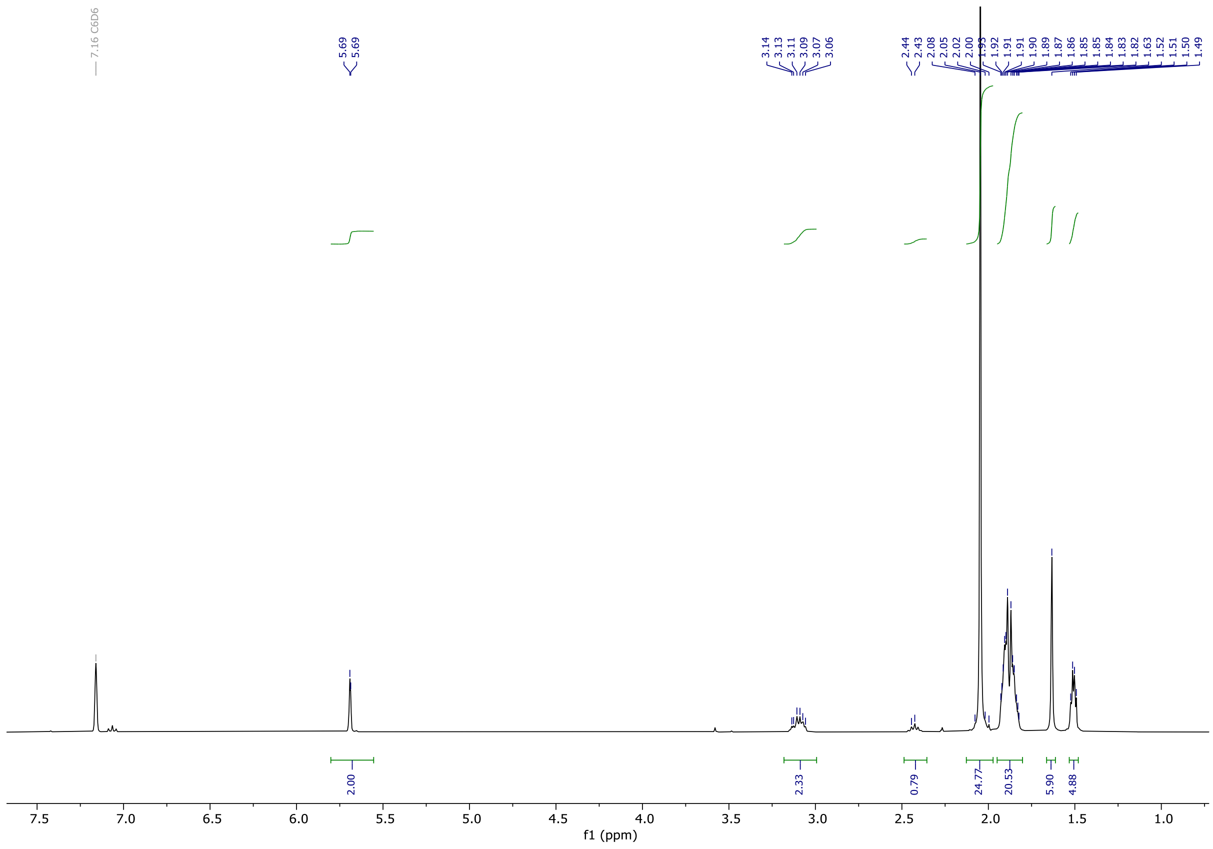


*Figure S14 [1]H NMR of* ***4b*** *in $C_6D_6$ after 15 minutes.*

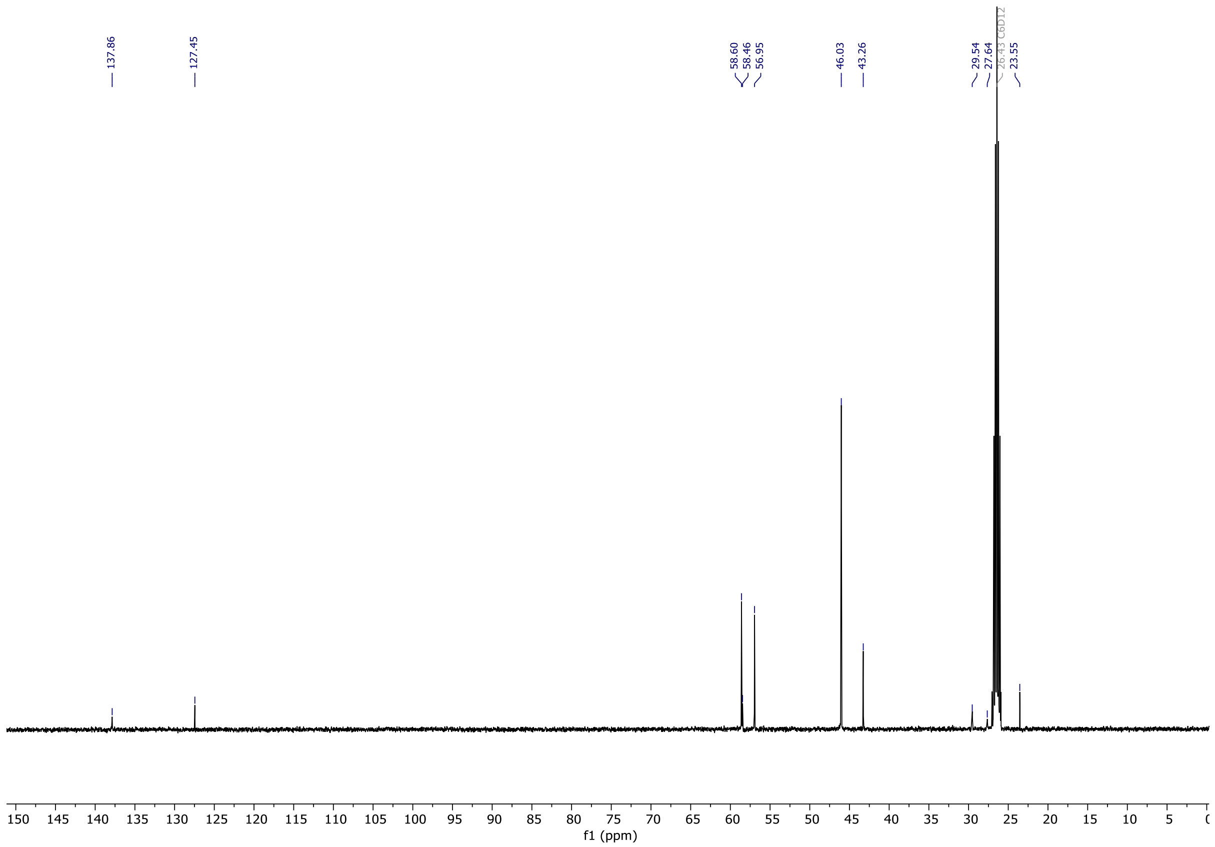


Figure S15 $^{13}C$ NMR of **4b** in $C_6D_{12}$ after 1 h.

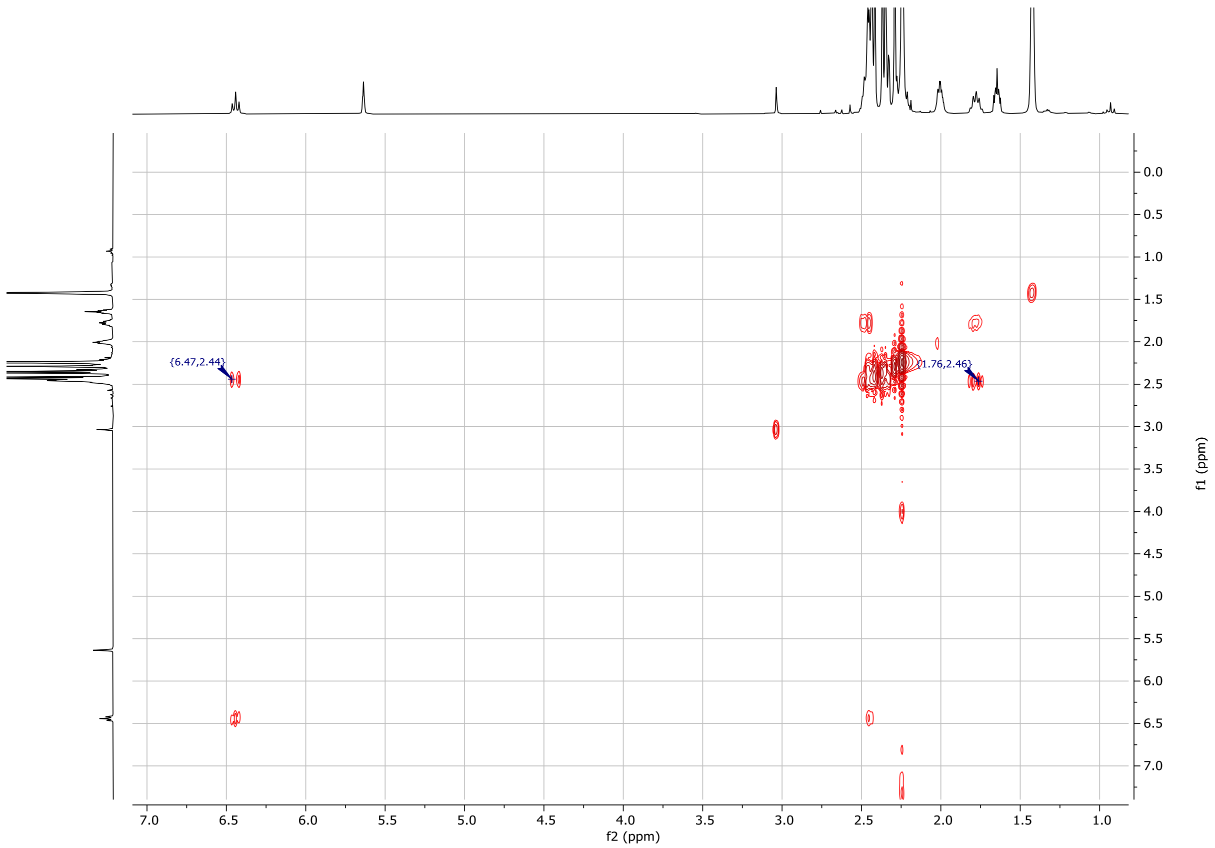


Figure S16 $^{1}H$ COSY NMR of **4b** in $C_6D_{12}$ showing signals relating to cyclohexyl ring in $^{1}H$ spectrum masked by PMDETA signals.

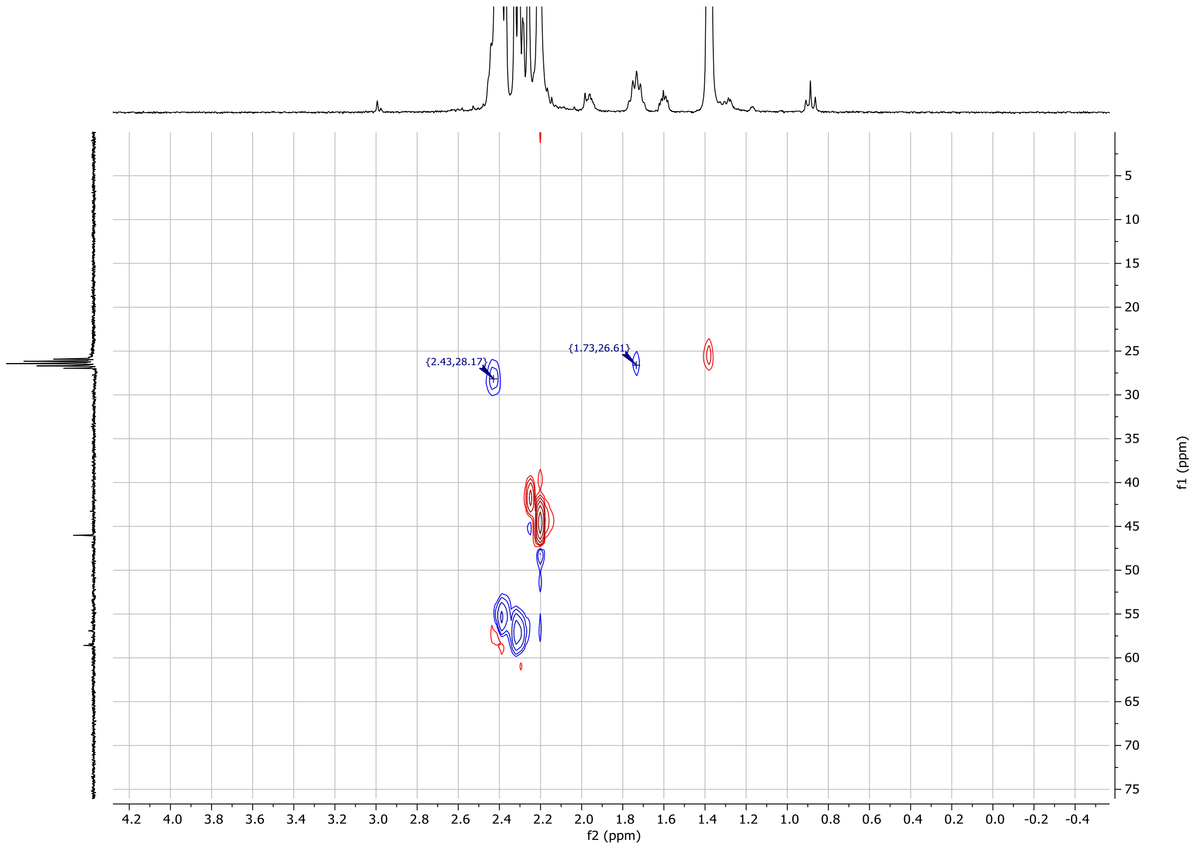


*Figure S17 $^{1}H/^{13}C$ HSQC NMR of **4b** in $C_6D_{12}$ showing carbon signals for **4b** are found under the residual solvent peak.*

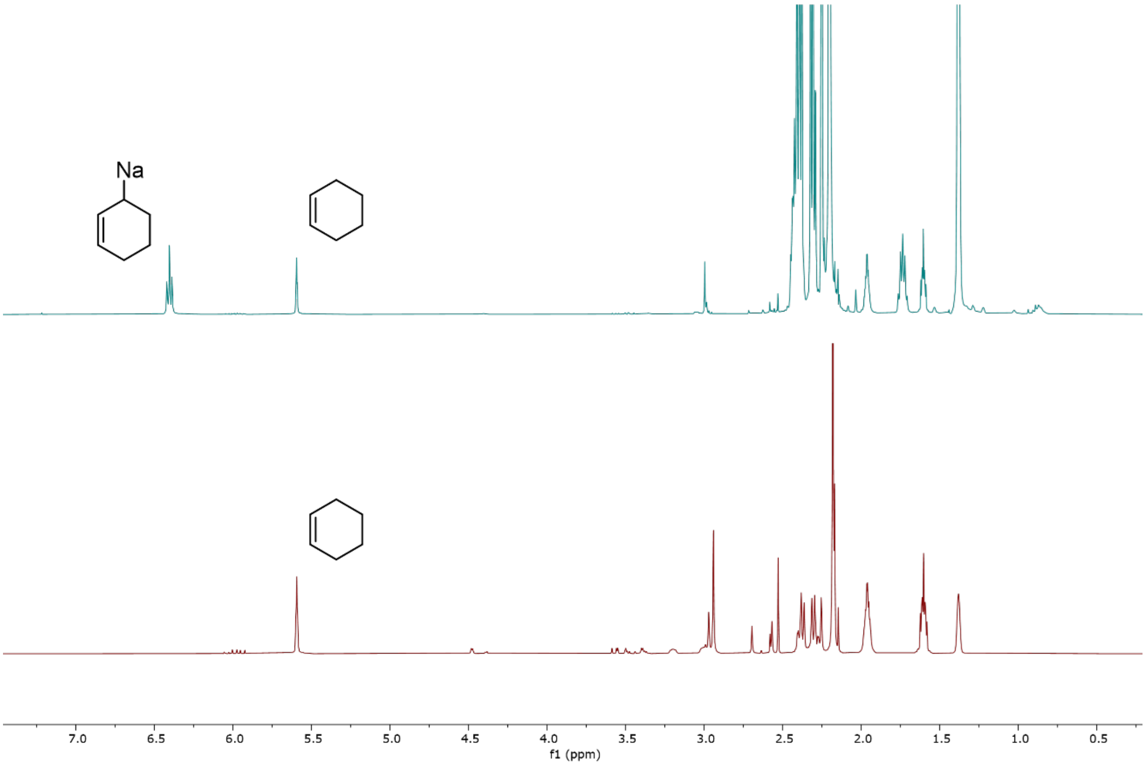


*Figure S18 $^{1}H$ NMR spectra of **4b** stacked to show formation of cyclohexene over 1 h. Green – 15 min, Red – 1 hour.*

**$CO_2$ Quench of 4b**

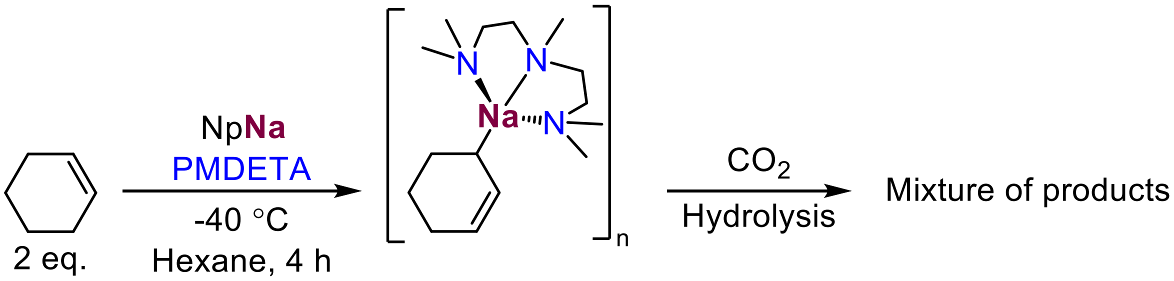


In an argon-flushed Schlenk flask, $NaCH_2CMe_3$ (0.5 mmol, 47 mg) was suspended in hexane (5 mL) at -40 °C, to which PMDETA (0.5 mmol, 0.105mL) was added via syringe. Cyclohexene (1 mmol, 0.100 mL) was added, and the reaction was stirred at -40 °C for 4 hours. $CO_2$ was added and the resulting suspension was slowly brought to ambient temperature and stirred for an additional 1 hour. The reaction was quenched with $H_2O$ and extracted from 1 M HCl with $Et_2O$. The organics were collected, dried over $MgSO_4$ and concentrated in vacuo.

$^1$H NMR of the crude reaction mixture showed several products present.

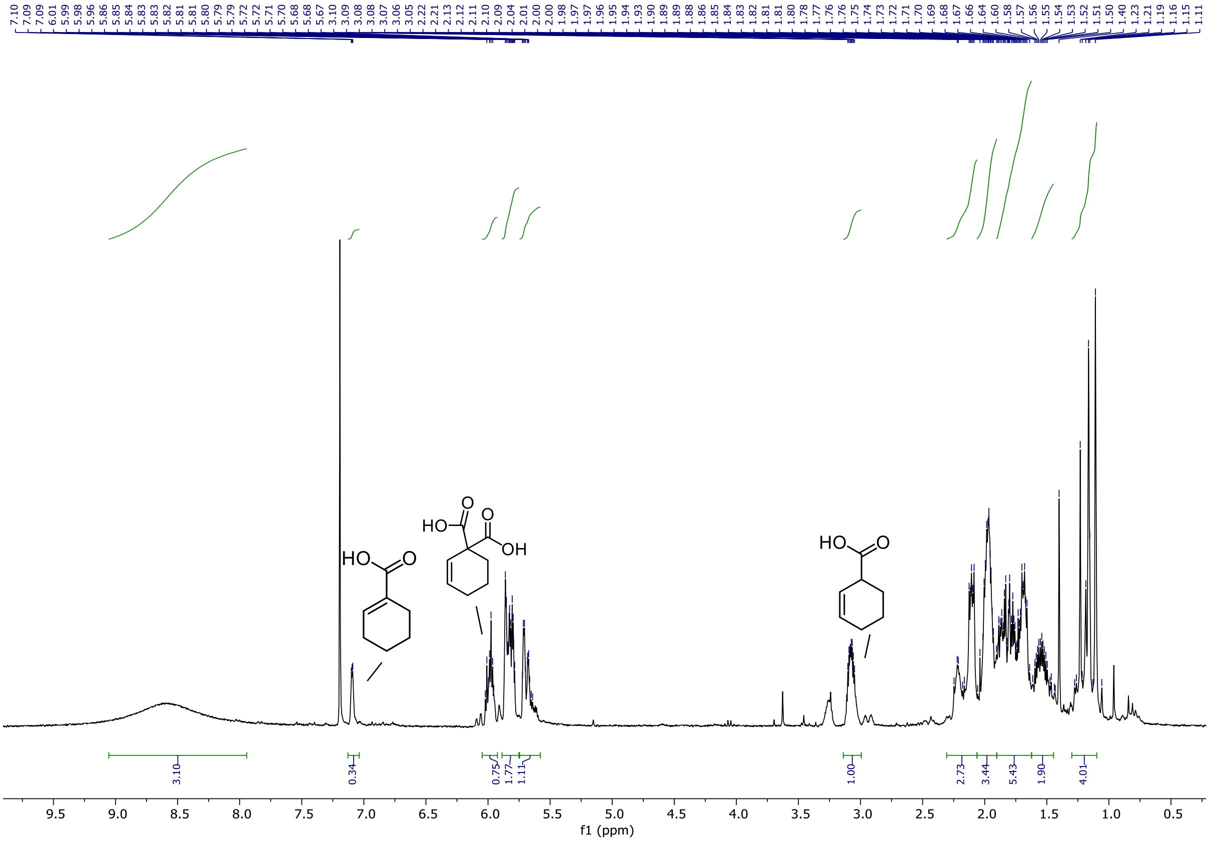


*Figure S19 $^1$H NMR of crude from reaction of **4b** with $CO_2$ with signals of major products identified.*

**Weinreb Amide Quench of 4b**

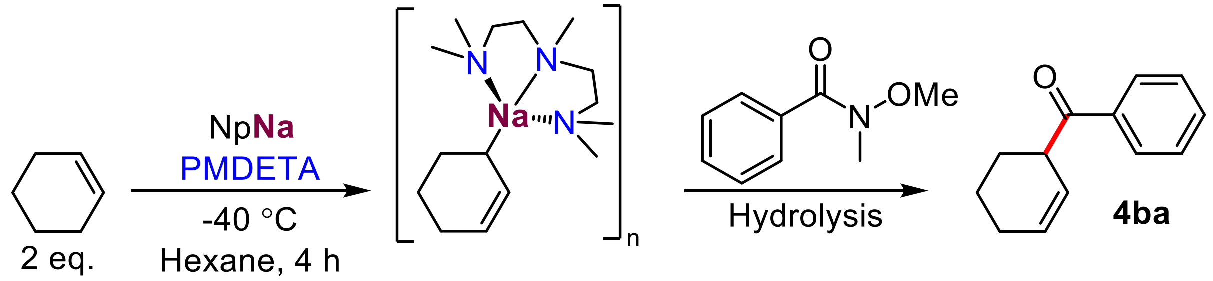


In an argon-flushed Schlenk flask, $NaCH_2CMe_3$ (0.5 mmol, 47 mg) was suspended in hexane (5 mL) at -40 °C, to which PMDETA (0.5 mmol, 0.105mL) was added via syringe. Cyclohexene (1 mmol, 0.100 mL) was added, and the reaction was stirred at -40 °C for 4 hours. N-methoxy-N-methylbenzamide (0.5 mmol, 0.076 mL) was added and the resulting suspension was slowly brought to ambient temperature and stirred for an additional 1 hour. The reaction was quenched with $H_2O$ and extracted from $NH_4Cl_{(aq)}$ with $Et_2O$. The organics were collected, dried over $MgSO_4$ and concentrated in vacuo. The crude product was purified by column chromatography (100% hexane – 90:10 hexane:EtOAt) to afford the quenched product **4ba** as a pale yellow oil (49 mg, 0.29 mmol, 58%). NMR consistent with literature reports.[3]

**$^1$H NMR** (300 MHz, $CDCl_3$) δ 8.00 – 7.92 (m, 2H), 7.56 (ddt, *J* = 8.3, 6.4, 1.4 Hz, 1H), 7.51 – 7.43 (m, 2H), 5.93 (dtd, *J* = 9.9, 3.7, 2.4 Hz, 1H), 5.80 – 5.68 (m, 1H), 4.09 (ddt, *J* = 8.2, 5.6, 2.8 Hz, 1H), 2.08 (dddd, *J* = 9.5, 7.2, 4.9, 3.1 Hz, 2H), 2.03 – 1.91 (m, 1H), 1.91 – 1.77 (m, 2H), 1.77 – 1.61 (m, 1H).

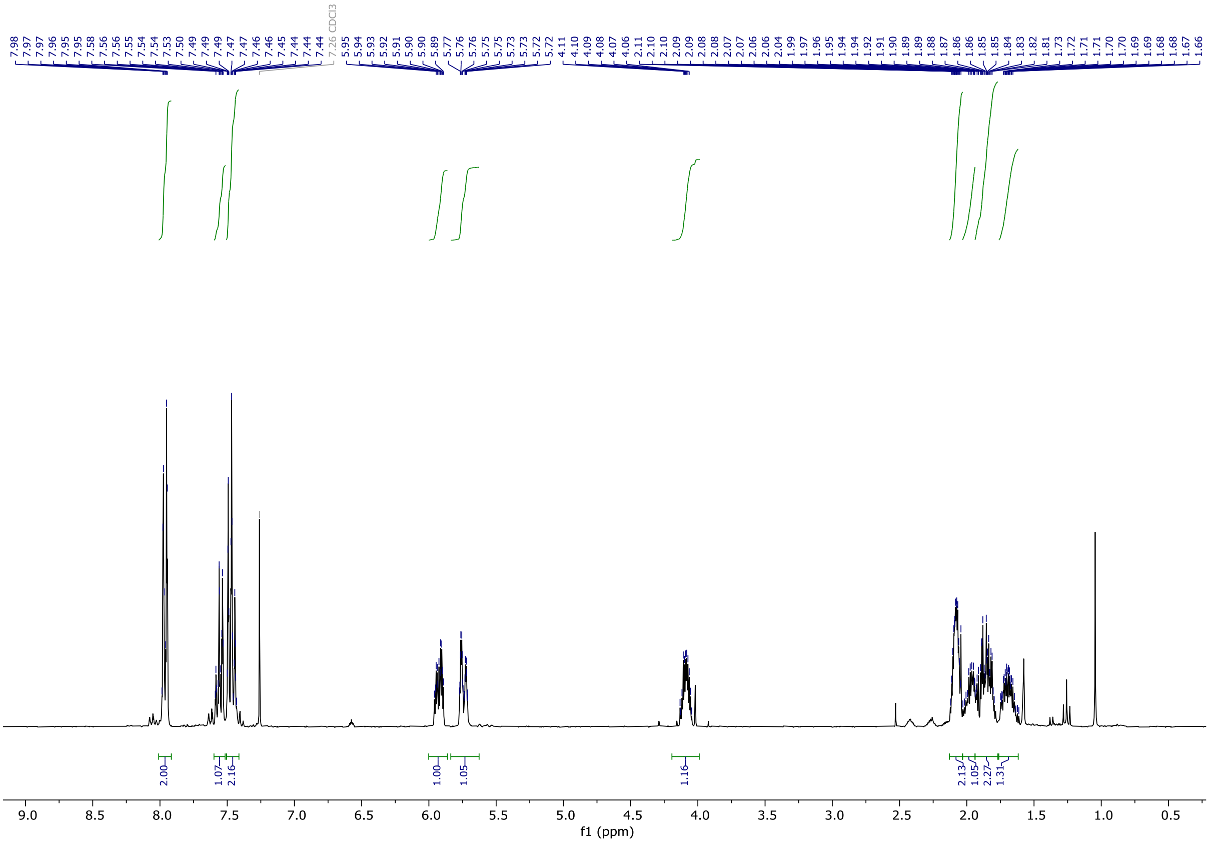


*Figure S20 $^{1}$H NMR of product of reaction of **4b** with phenyl Weinreb amide.*

# $^{1}$H-DOSY NMR Spectroscopic Analysis

Diffusion Ordered Spectroscopy (DOSY) experiments were conducted by NMR using the External Calibration Curve (ECC) method at 15 mM in $C_6D_6$ as described by Stalke.[4] Data was accumulated by linearly varying the diffusion encoding gradients over a range of 2% to 95% for 32 gradient values. The signal decay dimension on the pseudo-2D data was generated by Fourier transformation of the time-domain data. The diffusion profile and coefficients were ascertained by use of the DOSY processing features of TopSpin software. The peak of residual proteo- solvent was used as an internal standard.

### [{$NaCH_2$***t***Bu}$_4$] (1a) $^{1}$H DOSY NMR

Using $C_6D_{12}$ as an internal standard, we calculated the estimated molecular weight of the **[$NaCH_2CMe_3$]** aggregate to be 403 g mol$^{-1}$ (+7% from theoretical tetramer molecular weight (376 g mol$^{-1}$)). Indicating a tetrameric structure in $C_6D_{12}$.

Average diffusion coefficient = 4.250 x $10^{-10}$ $m^2$ $s^{-1}$

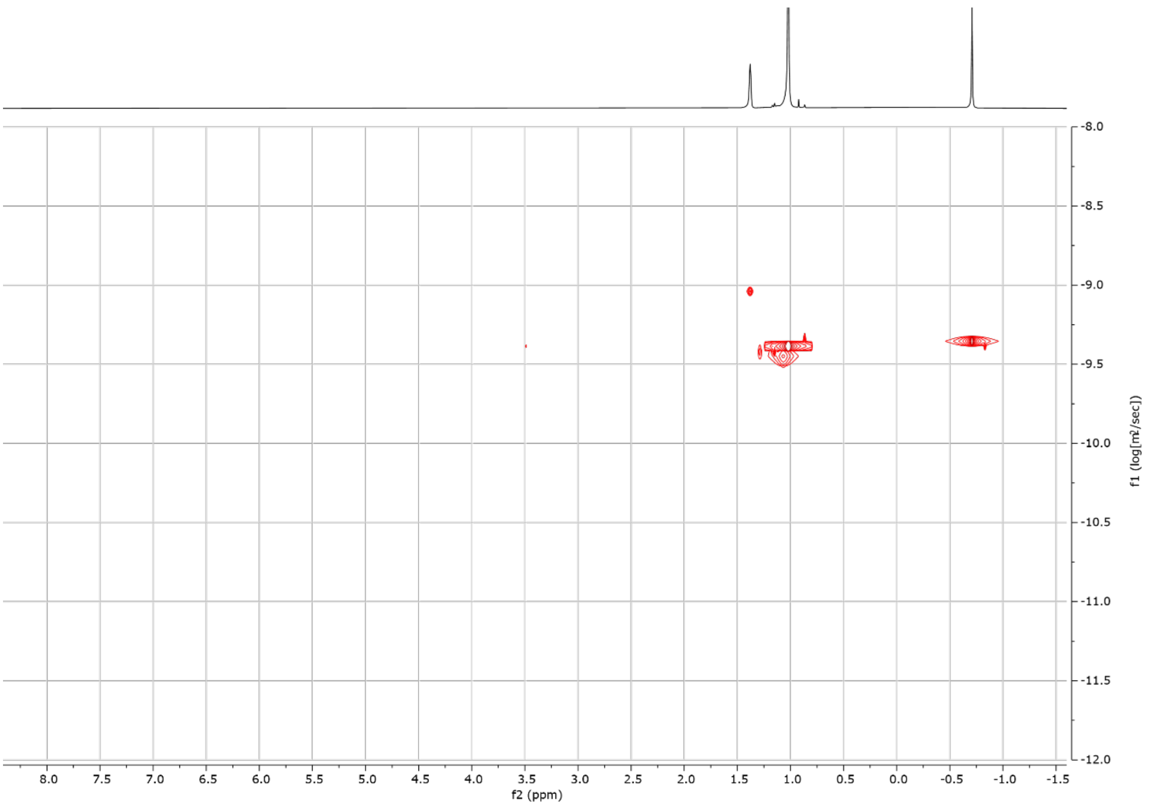


*Figure S21 $^{1}$H DOSY NMR of **1a** in $C_6D_{12}$.*

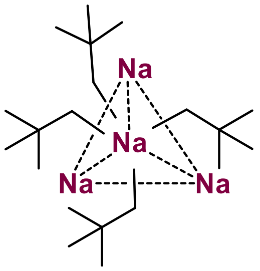


Molecular weight = 376 g mol$^{-1}$

## [{$NaCH_2$*t*Bu}$_2$$TMEDA_2$] (1b) $^1$H DOSY NMR

Using $C_6D_{12}$ as an internal standard, we calculated the estimated molecular weight of the **[{$NaCH_2CMe_3$}TMEDA]** aggregate to be 377 g mol$^{-1}$ (-11% from theoretical dimer molecular weight (420 g mol$^{-1}$)). Indicating a dimeric structure in $C_6D_{12}$.

Average diffusion coefficient = 5.054 x 10$^{-10}$ m$^2$ s$^{-1}$

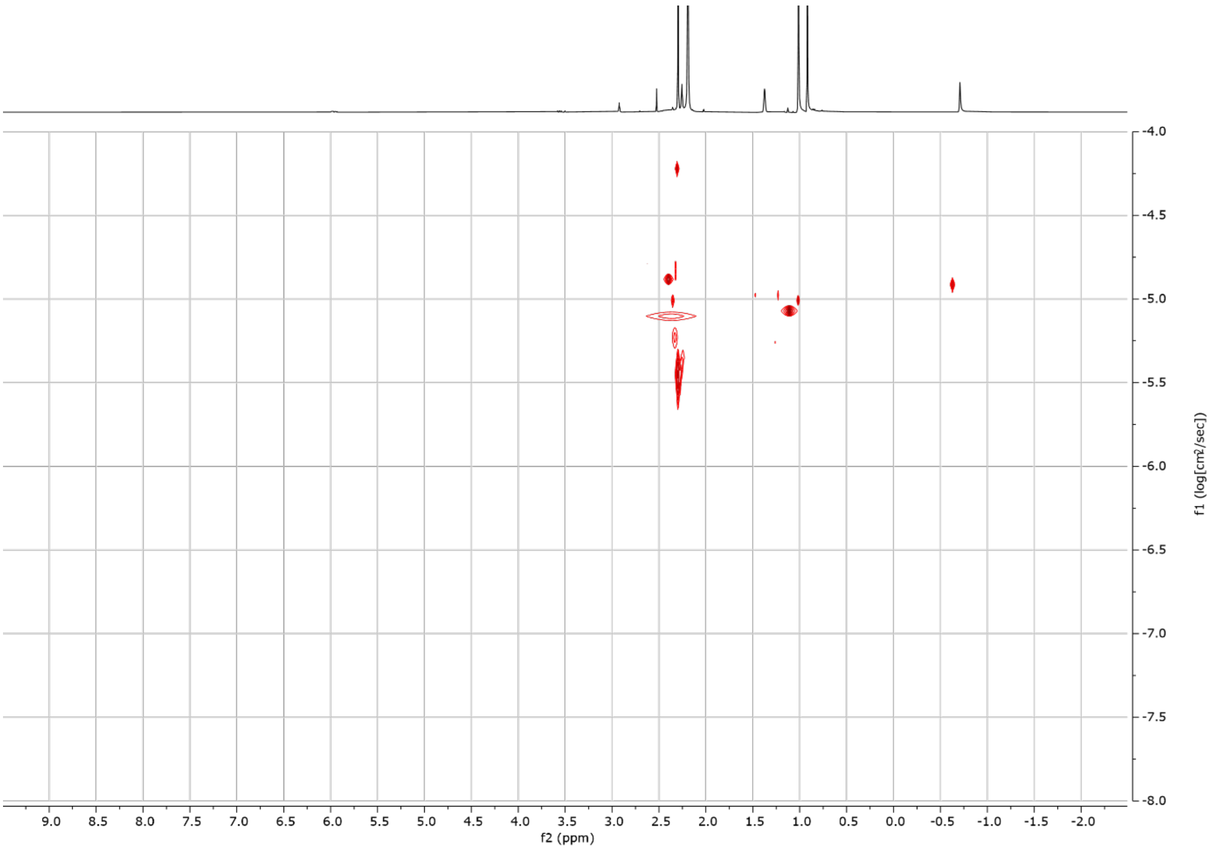


*Figure S22 $^1$H DOSY NMR of **1b** in $C_6D_{12}$.*

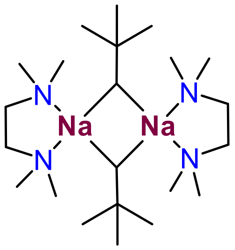


Molecular weight = 420 g $mol^{-1}$

# NMR Monitoring of Addition of PMDETA to 1a at Room Temperature

Addition of PMDETA to a solution of $NaCH_2CMe_3$ in $C_6D_{12}$ monitored by $^1H$ NMR, demonstrating rapid decomposition of the base at room temperature with the formation of neopentane.

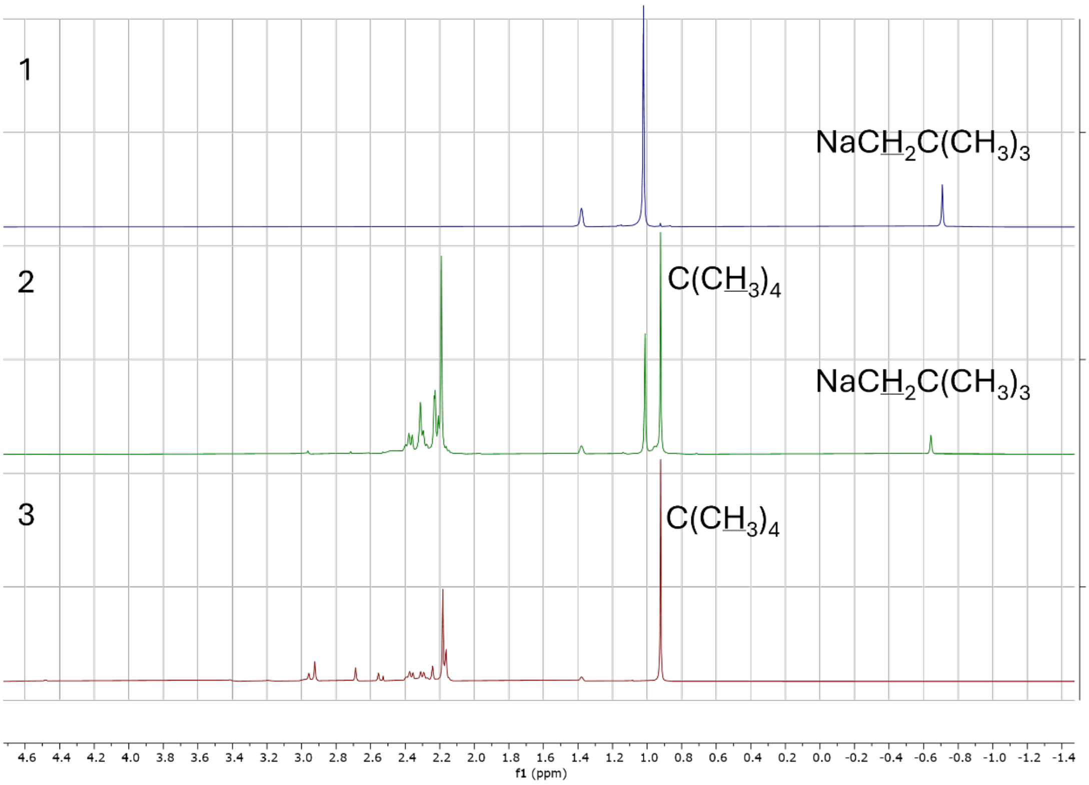


*Figure S23 Stacked $^1H$ NMR spectra of PMDETA addition to **1a** in $C_6D_{12}$ over time. 1) **1a** before addition of PMDETA. 2) 10 minutes after addition of PMDETA. 3) 1 h after PMDETA addition.*

# Synthesis of Organic Products

## Sodium Halogen Exchange of 1-Bromonaphthalene

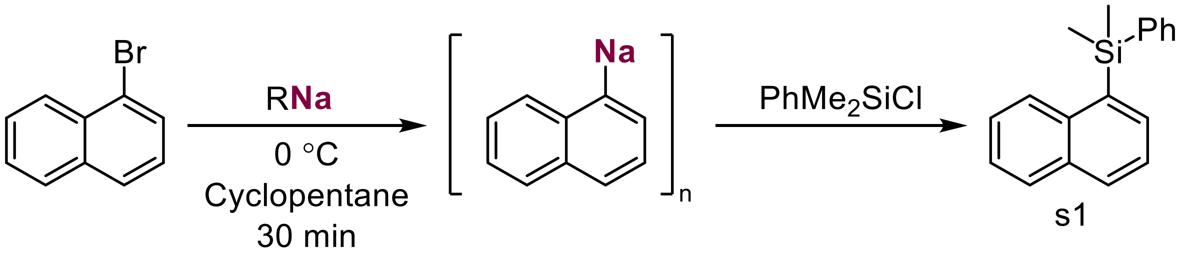


In an argon-flushed Schlenk flask, the alkyl sodium reagent (1 mmol, 2 eq) was suspended in cyclopentane (5 mL) at 0 °C, to which 1-bromonaphthalene (0.5 mmol, 0.070 mL) was added via syringe., the reaction was stirred at 0°C for 30 minutes. $PhMe_2SiCl$ (0.6 mmol, 0.100 mL) was added by syringe and the resulting suspension was slowly brought to ambient temperature and stirred for an additional 30 minutes. The reaction was quenched with $H_2O$ and extracted from saturated $NH_4Cl$ with EtOAc. The organics were collected, dried over $MgSO_4$ and concentrated in vacuo to give the product **s1** as a colourless oil. Yield of product was determined by $^1H$ NMR against an internal standard of $C_6Me_6$ (10 mol%).

**$^1$H NMR** (300 MHz, $CDCl_3$) δ 7.93 (dt, *J* = 8.1, 1.0 Hz, 2H), 7.89 – 7.83 (m, 1H), 7.73 (dd, *J* = 6.8, 1.3 Hz, 1H), 7.59 – 7.52 (m, 2H), 7.51 – 7.44 (m, 2H), 7.41 – 7.32 (m, 4H), 0.71 (s, 6H).

NaNp yield = 94%

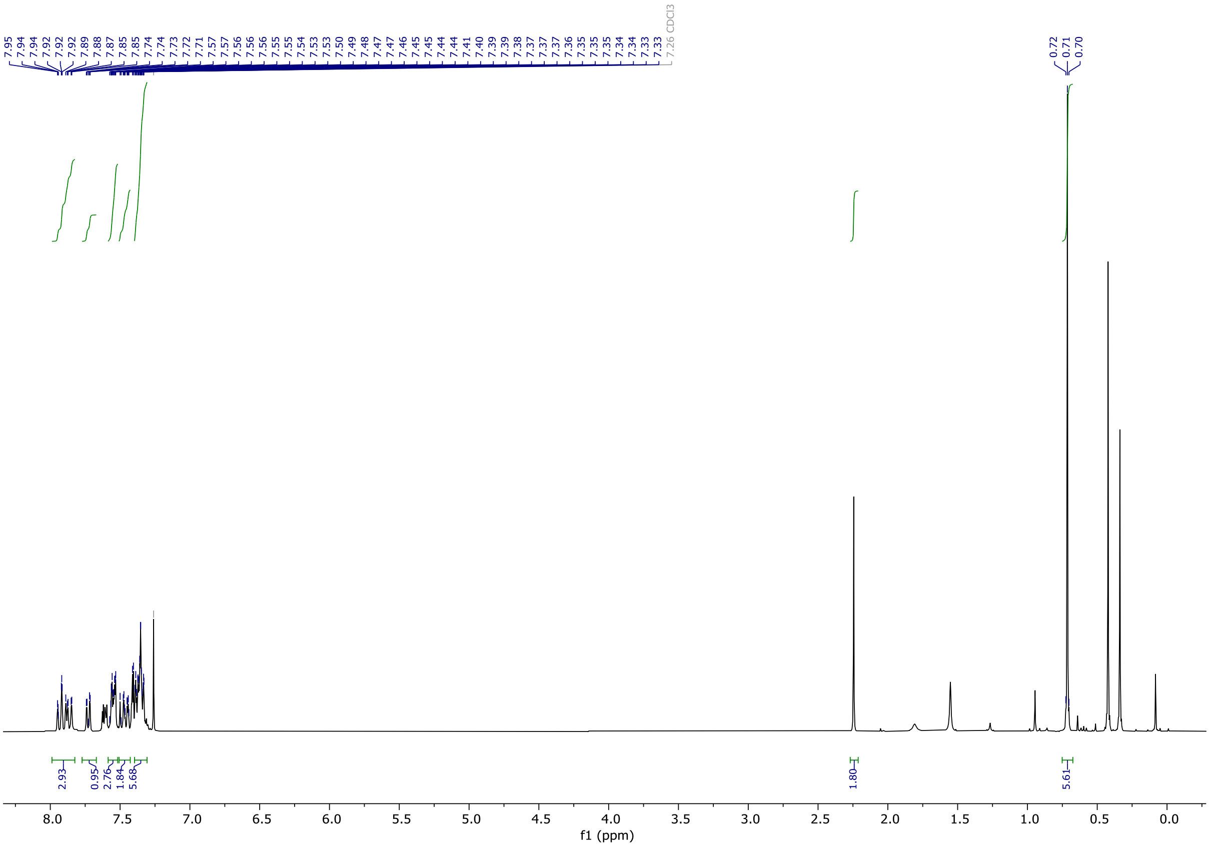


*Figure S24 $^1$H NMR of **s1** formed from NaNp, in $CDCl_3$ containing 10 mol% $C_6Me_6$*

*n*BuNa yield = 54%

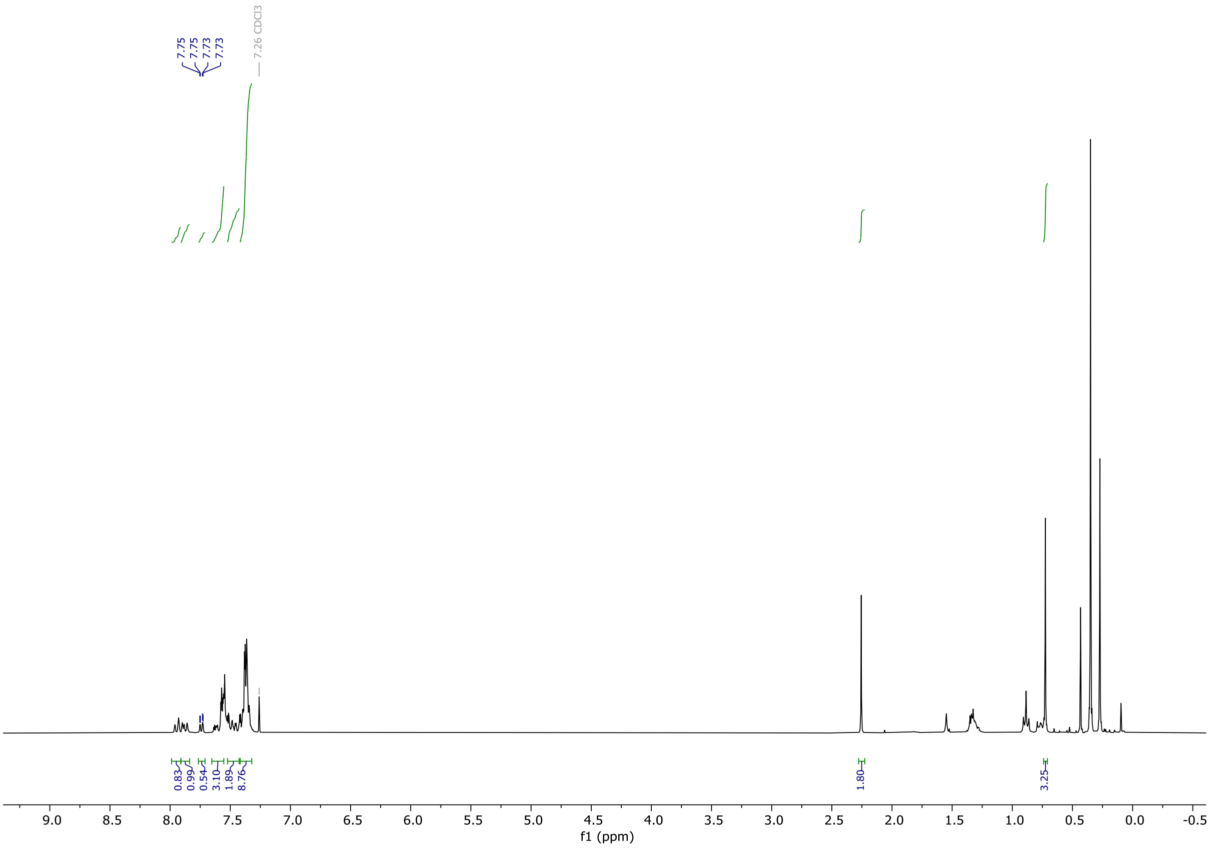

*Figure S25 ¹H NMR of **s1** formed from nBuNa, in $CDCl_3$ containing 10 mol% $C_6Me_6$*

## General Procedure for Carboxylations (General procedure A)

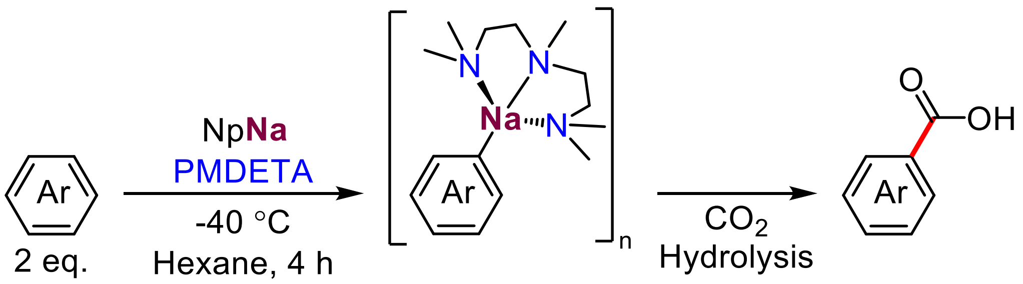


In an argon-flushed Schlenk flask, $NaCH_2tBu$ (0.5 mmol, 47 mg) was suspended in hexane (5 mL) at -40 °C, to which PMDETA (0.5 mmol, 0.105mL) was added via syringe. The aryl or

vinyl derivative (1 mmol) was added, and the reaction was stirred at -40 °C for 4 hours. $CO_2$ was subsequently bubbled through the solution and the resulting suspension was slowly brought to ambient temperature and stirred for an additional 1 hour. The reaction was quenched with $H_2O$ and extracted from 1 M HCl with $Et_2O$. The organics were collected, and an acid base extraction was carried out on the crude reaction mixture. The organics were dried over $MgSO_4$ and concentrated in vacuo to afford the pure carboxylic acids.

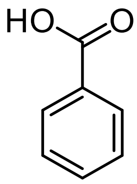


**Benzoic acid**

Prepared according to the general procedure A and volatiles removed under vacuum, affording **2a** as a colourless crystalline solid (51 mg, 0.42 mmol, 83%). NMR consistent with literature reports.[5]

**$^1$H NMR** (300 MHz, $CDCl_3$) δ 8.18 – 8.08 (m, 2H), 7.63 (tt, *J* = 6.8, 1.4 Hz, 1H), 7.55 – 7.43 (m, 2H).

**$^{13}$C NMR** (75 MHz, $CDCl_3$) δ 172.2, 134.0, 130.4, 129.4, 128.6.

**HRMS**: ESI [M-H]- $C_7H_5O_2$ Calc. 121.0295 found 121.0295.

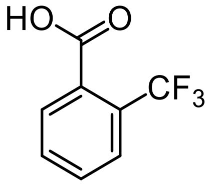


**2-(trifluoromethyl)benzoic acid**

Prepared according to the general procedure A and volatiles removed under vacuum, affording **2b** as a colourless crystalline solid (75 mg, 0.40 mmol, 79%). NMR consistent with literature reports.[6]

**$^1$H NMR** (300 MHz, $CDCl_3$) δ 7.97 – 7.86 (m, 1H), 7.81 – 7.68 (m, 1H), 7.67 – 7.53 (m, 2H).

**$^{13}$C NMR** (75 MHz, $CDCl_3$) δ 171.9, 132.4, 132.0, 131.3, 129.9, 129.8, 129.5, 127.17 (q, *J* = 5.7 Hz), 123.33 (d, *J* = 273.5 Hz).

**$^{19}$F NMR** (282 MHz, $CDCl_3$) δ -59.4.

**HRMS**: ESI [M-H]- $C_8H_4O_2F_3$ Calc. 189.0169 found 189.0170.

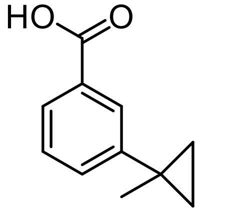 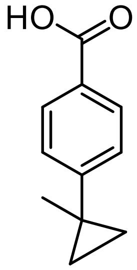

**3 and 4 -(1-methylcyclopropyl)benzoic acid**

**2c** prepared according to the general procedure A and volatiles removed under vacuum, affording **2c** as a colourless crystalline solid (75 mg, 0.43 mmol, 85%). NMR consistent with literature reports.[7]

*m*- isomer **$^{1}$H NMR** (300 MHz, $CDCl_3$) δ 12.56 (s, 1H), 8.05 – 8.01 (m, 1H), 7.94 (dt, *J* = 7.7, 1.4 Hz, 1H), 7.56 – 7.47 (m, 1H), 7.39 (t, *J* = 7.7 Hz, 1H), 1.46 (s, 3H), 1.02 – 0.87 (m, 2H), 0.87 – 0.78 (m, 2H).

*p*- isomer **$^{1}$H NMR** (300 MHz, $CDCl_3$) δ 12.56 (s, 1H), 8.08 – 8.01 (m, 2H), 7.32 (d, *J* = 8.2 Hz, 2H), 1.46 (s, 3H), 1.02 – 0.87 (m, 2H), 0.87 – 0.76 (m, 2H).

**$^{13}$C NMR** (75 MHz, $CDCl_3$) δ 172.9, 172.7, 154.0, 147.8, 132.4, 130.4, 129.4, 128.6, 128.5, 127.5, 126.4, 126.3, 25.6, 24.9, 19.8, 17.1, 15.9.

**HRMS**: ESI [M-H]- $C_{11}H_{11}O_2$ Calc. 175.0765 found 175.0754.

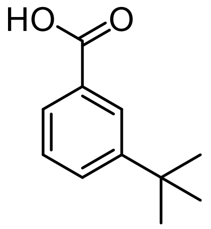 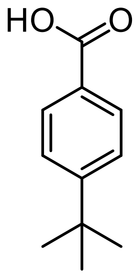

**3 and 4 -(tert-butyl)benzoic acid**

Prepared according to the general procedure A and volatiles removed under vacuum, affording **2d** as a colourless crystalline solid (69 mg, 0.39 mmol, 77%). NMR consistent with literature reports.[8][9]

*m*- isomer **$^{1}$H NMR** (400 MHz, $CDCl_3$) δ 8.16 (t, *J* = 1.9 Hz, 1H), 7.95 (dt, *J* = 7.7, 1.4 Hz, 1H), 7.69 – 7.61 (m, 1H), 7.42 (t, *J* = 7.8 Hz, 1H), 1.37 (s, 11H).

*p*- isomer **$^{1}$H NMR** (400 MHz, $CDCl_3$) δ 8.09 – 8.02 (m, 2H), 7.54 – 7.46 (m, 2H), 1.36 (s, 7H).

*m*- isomer **$^{13}$C NMR** (101 MHz, $CDCl_3$) δ 172.3, 151.8, 131.1, 130.3, 129.2, 128.4, 127.6, 127.3, 35.4, 31.4.

*p*- isomer **$^{13}$C NMR** (101 MHz, $CDCl_3$) δ 172.6, 157.8, 130.3, 126.7, 125.6, 35.4, 31.3.

**HRMS**: ESI [M-H]- $C_{11}H_{13}O_2$ Calc. 177.0921 found 177.0921.

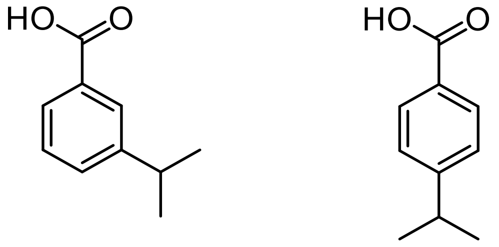

**3 and 4 -isopropylbenzoic acid**

Prepared according to the general procedure A and volatiles removed under vacuum, affording **2e** as a colourless waxy solid (66 mg, 0.40 mmol, 80%). NMR consistent with literature reports.[10][6]

*m*- isomer **$^1$H NMR** (300 MHz, $CDCl_3$) δ 8.00 (s, 1H), 7.95 (dt, *J* = 7.5, 1.5 Hz, 1H), 7.49 (dt, *J* = 7.6, 1.7 Hz, 1H), 7.40 (t, *J* = 7.6 Hz, 1H), 3.00 (hept, *J* = 6.9 Hz, 1H), 1.30 (d, *J* = 6.9 Hz, 6H).

*p*- isomer **$^1$H NMR** (300 MHz, $CDCl_3$) δ 8.06 (d, *J* = 8.3 Hz, 2H), 7.34 (d, *J* = 8.2 Hz, 2H), 3.00 (hept, *J* = 6.9 Hz, 1H), 1.29 (d, *J* = 4.2 Hz, 6H).

*m*- isomer **$^{13}$C NMR** (75 MHz, $CDCl_3$) δ 172.7, 149.5, 132.3, 129.4, 128.6, 128.4, 127.9, 34.2, 24.0.

*p*- isomer **$^{13}$C NMR** (75 MHz, $CDCl_3$) δ 130.6, 126.8, 34.5, 23.8. (quaternary carbons not observed)

**HRMS**: ESI [M-H]- $C_{10}H_{11}O_2$ Calc. 163.0765 found 163.0765.

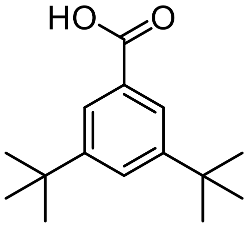

**3,5-di-tert-butylbenzoic acid**

Prepared according to the general procedure A and volatiles removed under vacuum, affording **2f** as a white crystalline solid (94 mg, 0.40 mmol, 80%). NMR consistent with literature reports.[11]

**$^1$H NMR** (300 MHz, $CDCl_3$) δ 7.98 (d, *J* = 1.9 Hz, 2H), 7.69 (t, *J* = 1.9 Hz, 1H), 1.37 (s, 18H).

**$^{13}$C NMR** (75 MHz, $CDCl_3$) δ 172.6, 151.4, 128.7, 128.2, 124.6, 35.1, 31.5.

**HRMS**: ESI [M-H]- $C_{15}H_{21}O_2$ Calc. 233.1547 found 233.1547.

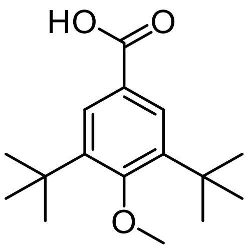

**3,5-di-tert-butyl-4-methoxybenzoic acid**

Prepared according to the general procedure A and volatiles removed under vacuum, affording **2g** as a white crystalline solid (90 mg, 0.34 mmol, 68%). NMR consistent with literature reports.[12]

**$^1$H NMR** (300 MHz, $CDCl_3$) δ 8.03 (d, *J* = 0.8 Hz, 2H), 3.73 (s, 3H), 1.46 (s, 18H).

**$^{13}$C NMR** (75 MHz, $CDCl_3$) δ 172.2, 164.7, 144.3, 129.0, 123.4, 64.5, 35.9, 31.9.

**HRMS**: ESI [M-H]- $C_{16}H_{23}O_3$ Calc. 263.1653 found 263.1652.

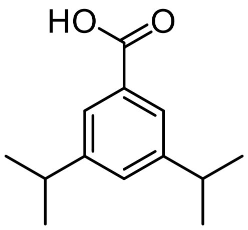

**3,5-diisopropylbenzoic acid**

Prepared according to the general procedure A and volatiles removed under vacuum, affording **2h** as a colourless crystalline solid (71 mg, 0.35 mmol, 69%).

**$^1$H NMR** (300 MHz, $CDCl_3$) δ 7.82 (d, *J* = 1.7 Hz, 2H), 7.33 (t, *J* = 1.8 Hz, 1H), 2.97 (hept, *J* = 7.0 Hz, 2H), 1.29 (d, *J* = 6.9 Hz, 12H).

**$^{13}$C NMR** (75 MHz, $CDCl_3$) δ 172.5, 149.4, 130.8, 129.4, 125.9, 34.2, 24.1.

**HRMS**: ESI [M-H]- $C_{13}H_{17}O_2$ Calc. 205.1234 found 205.1233.

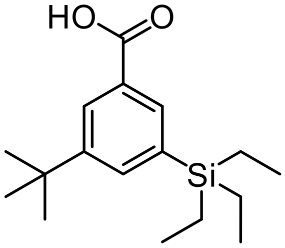

**3-(tert-butyl)-5-(triethylsilyl)benzoic acid**

Prepared according to the general procedure A and volatiles removed under vacuum. Further purified by column chromatography (Hexane:$Et_2O$ 100:0 to 98:2) affording **2i** as a colourless oil (92 mg, 0.31 mmol, 63%).

**$^1$H NMR** (300 MHz, $CDCl_3$) δ 8.13 (t, *J* = 1.8 Hz, 1H), 8.09 – 8.04 (m, 1H), 7.76 (dd, *J* = 2.1, 1.1 Hz, 1H), 1.37 (s, 9H), 0.99 (t, *J* = 7.7 Hz, 9H), 0.84 (q, *J* = 7.2 Hz, 6H).

**$^{13}$C NMR** (75 MHz, $CDCl_3$) δ 173.0, 150.6, 137.9, 136.8, 133.3, 128.4, 127.6, 34.9, 31.4, 7.5, 3.5.

**HRMS**: ESI [M-H]- $C_{17}H_{27}O_2{}^{28}Si$ Calc. 291.1786 found 291.1786.

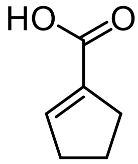


**cyclopent-1-ene-1-carboxylic acid**

Prepared according to the general procedure A and volatiles removed under vacuum, affording **2j** as a beige crystalline solid (37 mg, 0.33 mmol, 66%). NMR consistent with literature reports.[13]

**$^1$H NMR** (300 MHz, $CDCl_3$) δ 6.93 (p, *J* = 2.1 Hz, 1H), 2.65 – 2.47 (m, 4H), 1.99 (p, *J* = 7.7 Hz, 2H).

**$^{13}$C NMR** (75 MHz, $CDCl_3$) δ 170.7, 147.0, 136.2, 33.8, 31.1, 23.3.

**HRMS**: ESI [M-H]- $C_6H_7O_2$ Calc. 111.0452 found 111.0452.

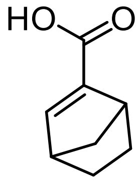


**bicyclo[2.2.1]hept-2-ene-2-carboxylic acid**

Prepared according to the general procedure A and volatiles removed under vacuum, affording **2k** as an off-white crystalline solid (46 mg, 0.34 mmol, 67%). NMR consistent with literature reports.[14]

**$^1$H NMR** (300 MHz, $CDCl_3$) δ 11.70 (s, 1H), 7.08 (d, *J* = 3.1 Hz, 1H), 3.26 (dq, *J* = 3.1, 1.5 Hz, 1H), 3.04 (dq, *J* = 3.3, 1.5 Hz, 1H), 1.86 – 1.67 (m, 2H), 1.55 – 1.46 (m, 1H), 1.21 (dq, *J* = 8.7, 1.3 Hz, 1H), 1.15 – 1.01 (m, 2H).

**$^{13}$C NMR** (75 MHz, $CDCl_3$) δ 170.7, 150.2, 140.4, 48.3, 43.9, 41.7, 24.6, 24.5.

**HRMS**: ESI [M-H]- $C_8H_9O_2$ Calc. 137.0608 found 137.0610.

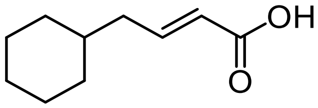


**(E)-4-cyclohexylbut-2-enoic acid**

Prepared according to the general procedure A and volatiles removed under vacuum, affording **2l** as a pale yellow oil (45 mg, 0.27 mmol, 54%). NMR consistent with literature reports.[15]

**$^1$H NMR** (300 MHz, $CDCl_3$) δ 7.07 (dt, *J* = 15.3, 7.5 Hz, 1H), 5.80 (dt, *J* = 15.5, 1.5 Hz, 1H), 2.12 (ddd, *J* = 7.8, 6.9, 1.5 Hz, 2H), 1.70 (dt, *J* = 14.0, 3.6 Hz, 5H), 1.54 – 1.34 (m, 1H), 1.30 – 1.11 (m, 3H), 1.03 – 0.79 (m, 2H).

**$^{13}$C NMR** (75 MHz, $CDCl_3$) δ 172.3, 151.6, 121.6, 40.4, 37.3, 33.3, 26.4, 26.3.

**HRMS**: ESI [M-H]- $C_{10}H_{15}O_2$ Calc. 167.1078 found 167.1079.

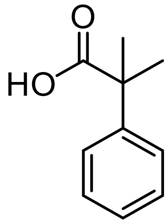

**2-methyl-2-phenylpropanoic acid**

Prepared according to a modified general procedure A with a metalation time of 12 h. After acid/base extraction, volatiles removed under vacuum, affording **2m** as a colourless crystalline solid (48 mg, 0.29 mmol, 58%). NMR consistent with literature reports.[16]

**$^1$H NMR** (300 MHz, $CDCl_3$) δ 7.46 – 7.36 (m, 2H), 7.41 – 7.29 (m, 2H), 7.32 – 7.20 (m, 1H), 1.61 (s, 6H).

**$^{13}$C NMR** (75 MHz, $CDCl_3$) δ 183.0, 143.9, 128.6, 127.1, 126.0, 46.4, 26.4.

**HRMS**: ESI [M-H]- $C_{10}H_{11}O_2$ Calc 163.0765 found 163.0764.

## General Procedure for Cross couplings (General procedure B)

In an argon-flushed Schlenk flask, $NaCH_2tBu$ (1 mmol, 94 mg) was suspended in hexane (5 mL) at -40 °C, to which PMDETA (1 mmol, 0.210 mL) was added via syringe. The aryl or vinyl derivative (1.5 mmol) was added, and the reaction was stirred at -40 °C for 4 hours. $B(O^iPr)_3$ (1 mmol, 0.230 mL) was subsequently added via syringe and the resulting solution was slowly brought to ambient temperature and stirred for an additional 14 hours. The hexane was removed in vacuo and replaced with toluene (10 mL), to the reaction mixture was added $Pd(dppf)Cl_2$ (0.04 mmol, 29 mg), and 1-bromo-4-fluorobenzene (0.8 mmol, 0.087 mL), followed by the addition of 0.2 mL of water. The reaction mixture was heated at 100°C for six hours. The reaction was quenched with $H_2O$ and extracted from aq. $NH_4Cl$ with EtOAc. The organics were collected, dried over $MgSO_4$ and concentrated in vacuo. The crude organic products were then purified by column chromatography.

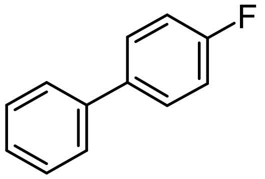


**5a 4-fluoro-1,1'-biphenyl**

Prepared according to the general procedure B and purified via silica column chromatography (Hexane:$Et_2O$ 100:0 to 95:5) to produce **5a** as a white crystalline solid (110 mg, 0.64 mmol, 80%). NMR consistent with literature reports.[17]

**$^1$H NMR** (300 MHz, $CDCl_3$) δ 7.58 – 7.51 (m, 4H), 7.48 – 7.40 (m, 2H), 7.38 – 7.31 (m, 1H), 7.18 – 7.06 (m, 2H).

**$^{13}$C NMR** (75 MHz, $CDCl_3$) δ 162.6 (d, *J* = 246.2 Hz), 140.4, 137.5 (d, *J* = 3.2 Hz), 129.0, 128.8 (d, *J* = 8.1 Hz), 127.4, 127.2, 115.8 (d, *J* = 21.4 Hz).

**$^{19}$F NMR** (282 MHz, $CDCl_3$) δ -115.9.

**HRMS**: ESI [M]+ $C_{12}H_9F$ Calc 172.0683 found 172.0684.

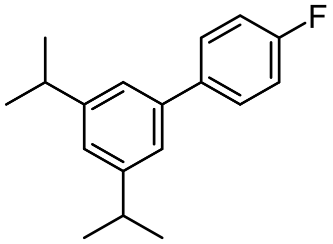


**5b 4'-fluoro-3,5-diisopropyl-1,1'-biphenyl**

Prepared according to the general procedure B and purified via silica column chromatography (Hexane 100%) to produce **5b** as a colourless oil (166 mg, 0.65 mmol, 81%).

**$^1$H NMR** (300 MHz, $CDCl_3$) δ 7.61 – 7.49 (m, 2H), 7.22 (d, *J* = 1.7 Hz, 2H), 7.17 – 7.06 (m, 3H), 2.96 (hept, *J* = 6.9 Hz, 2H), 1.31 (d, *J* = 6.9 Hz, 12H).

**$^{13}$C NMR** (75 MHz, $CDCl_3$) δ 162.5 (d, *J* = 245.7 Hz), 149.6, 140.4, 138.2, 128.9 (d, *J* = 7.9 Hz), 124.0, 122.9, 115.6 (d, *J* = 21.4 Hz), 34.4, 24.3.

**$^{19}$F NMR** (282 MHz, $CDCl_3$) δ -116.3.

**HRMS**: ESI [M]+ $C_{18}H_{21}F$ Calc 256.1622 found 256.1625.

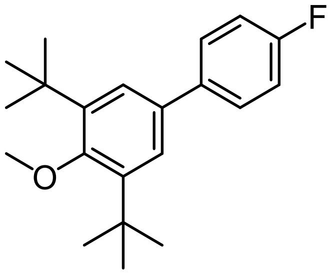


**5c 3,5-di-tert-butyl-4'-fluoro-4-methoxy-1,1'-biphenyl**

Prepared according to the general procedure B and purified via silica column chromatography (Hexane:$EtO_2$ 100:0 to 95:5) to produce **5c** as a white solid (197 mg, 0.62 mmol, 78%).

**$^1$H NMR** (300 MHz, $CDCl_3$) δ 7.56 – 7.47 (m, 2H), 7.42 (s, 2H), 7.16 – 7.07 (m, 2H), 3.75 (s, 3H), 1.50 (s, 18H).

**$^{13}$C NMR** (75 MHz, $CDCl_3$) δ 162.3 (d, $J$ = 245.5 Hz), 159.3, 144.2, 138.2, 138.1, 134.7, 128.7 (d, $J$ = 7.9 Hz), 125.5, 115.6 (d, $J$ = 21.3 Hz), 64.4, 36.1, 32.3.

**$^{19}$F NMR** (282 MHz, $CDCl_3$) δ -116.7.

**HRMS**: ESI [M]+ $C_{21}H_{27}OF$ Calc 314.2040 found 314.2044.

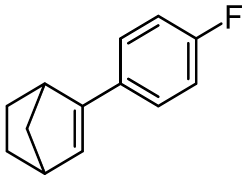


**5d 2-(4-fluorophenyl)bicyclo[2.2.1]hept-2-ene**

Prepared according to the general procedure B and purified via silica column chromatography (Hexane:$EtO_2$ 100:0 to 95:5) to produce **5d** as a colourless oil (61 mg, 0.33 mmol, 41%). NMR consistent with literature reports.

**$^1$H NMR** (300 MHz, $CDCl_3$) δ 7.40 – 7.23 (m, 2H), 6.98 – 6.83 (m, 2H), 6.14 (d, $J$ = 3.1 Hz, 1H), 3.20 (s, 1H), 2.92 (s, 1H), 1.69 (tdt, $J$ = 11.6, 6.2, 3.0 Hz, 2H), 1.50 – 1.43 (m, 1H), 1.22 – 1.15 (m, 1H), 1.14 – 0.98 (m, 2H).

**$^{13}$C NMR** (75 MHz, $CDCl_3$) δ 161.97 (d, $J$ = 245.5 Hz), 146.9, 132.1, 129.4, 129.3, 126.5 (d, $J$ = 7.8 Hz), 115.4 (d, $J$ = 21.4 Hz), 48.1, 43.7, 43.3, 27.0, 24.9.

**$^{19}$F NMR** (282 MHz, $CDCl_3$) δ -115.9.

**HRMS**: ESI [M]+ $C_{13}H_{13}F$ Calc 188.0996 found 188.0998.

# NMR spectra of isolated products

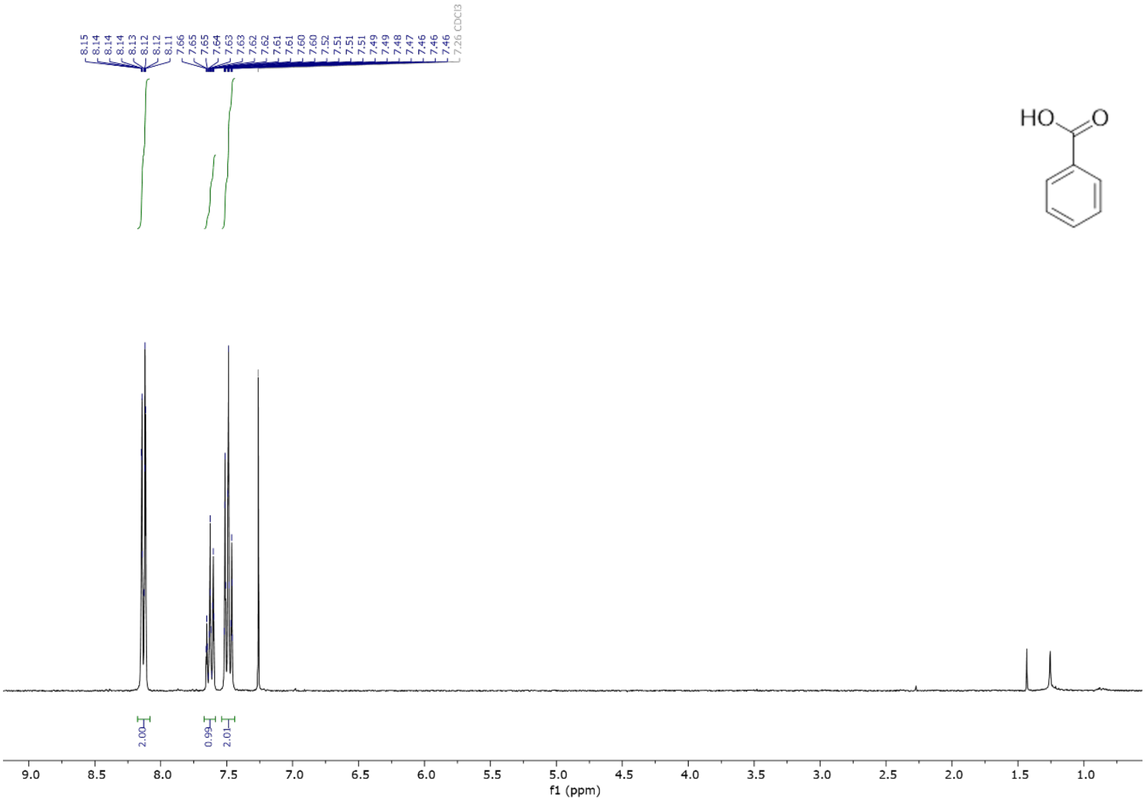

Figure S26[1]H NMR of **2a** in $CDCl_3$.

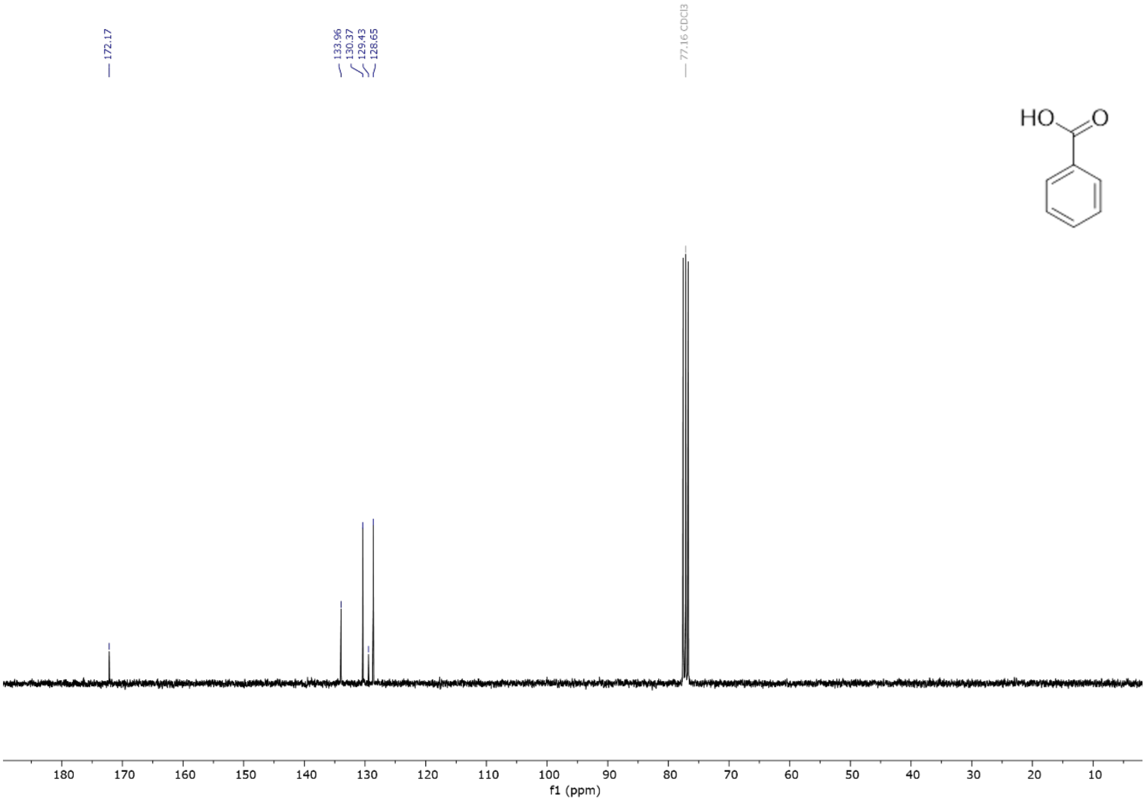

Figure S27 $^{13}$C NMR of **2a** in $CDCl_3$.

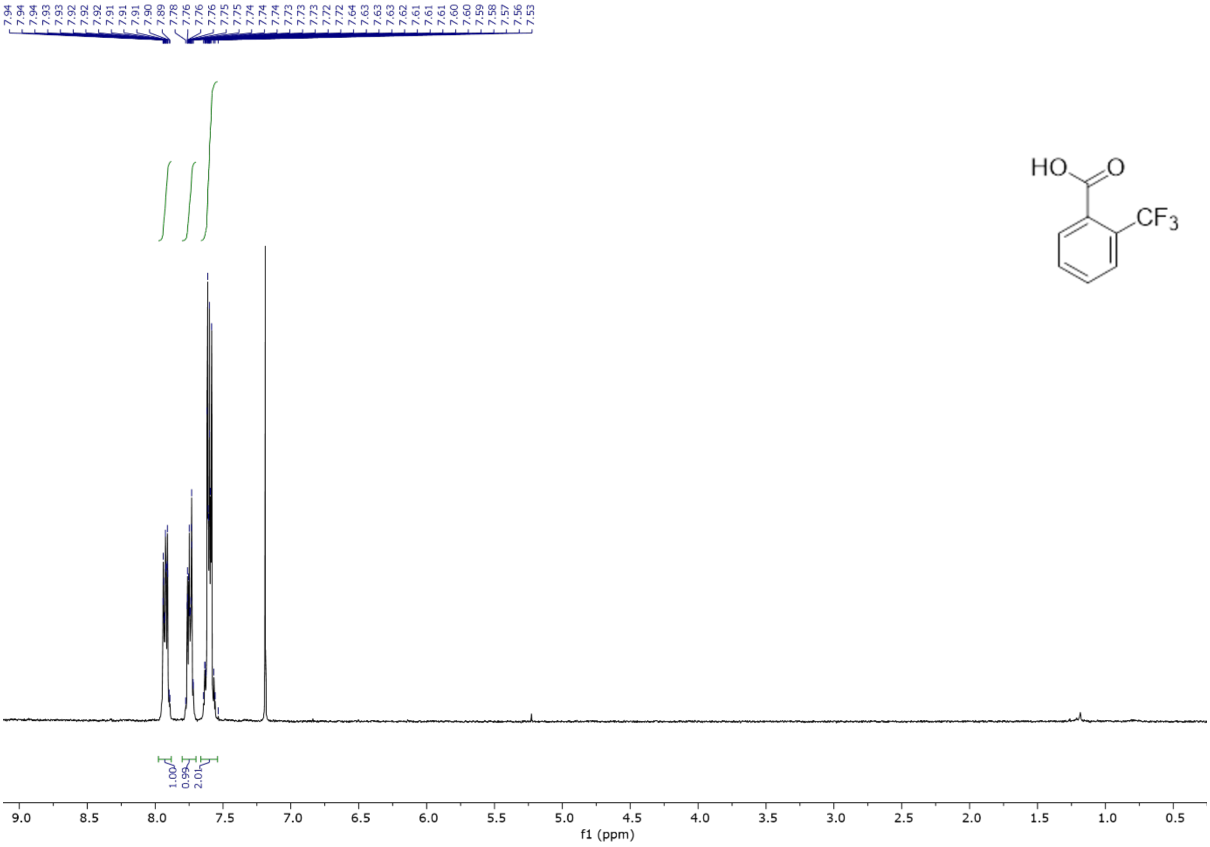


*Figure S28* $^{1}H$ *NMR of* ***2b*** *in* $CDCl_3$*.*

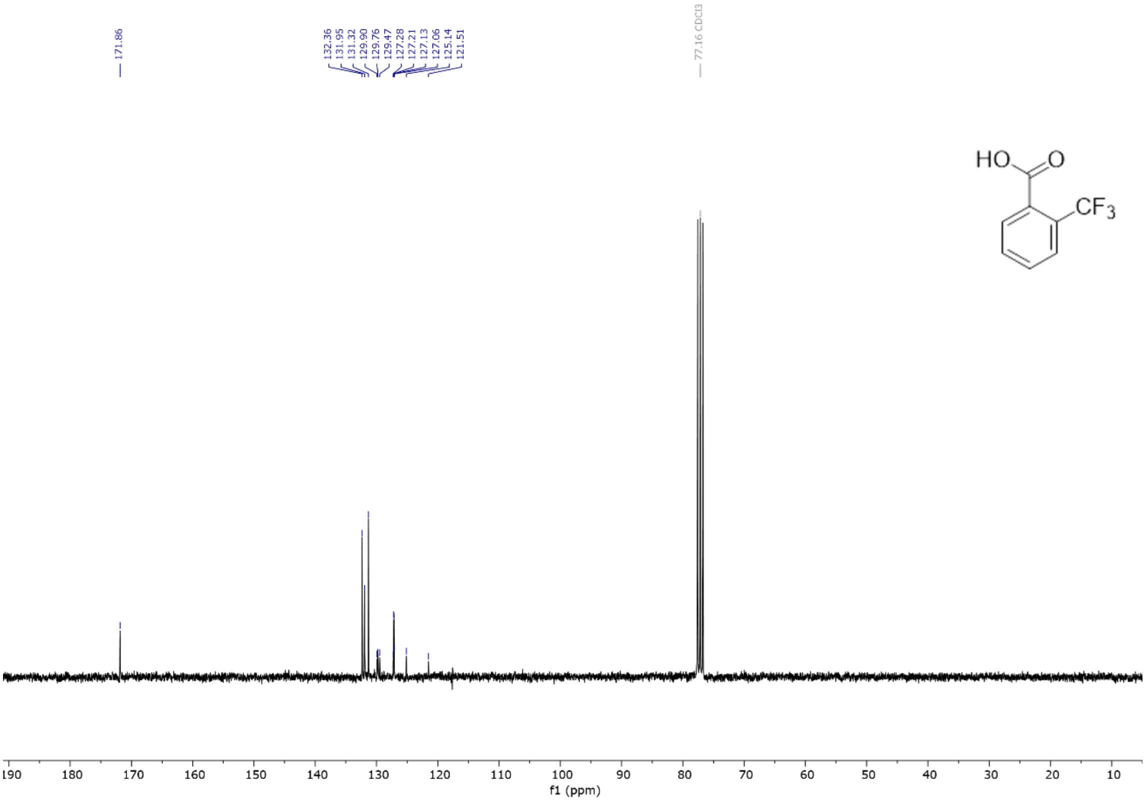


*Figure S29* $^{13}C$ *NMR of* ***2b*** *in* $CDCl_3$*.*

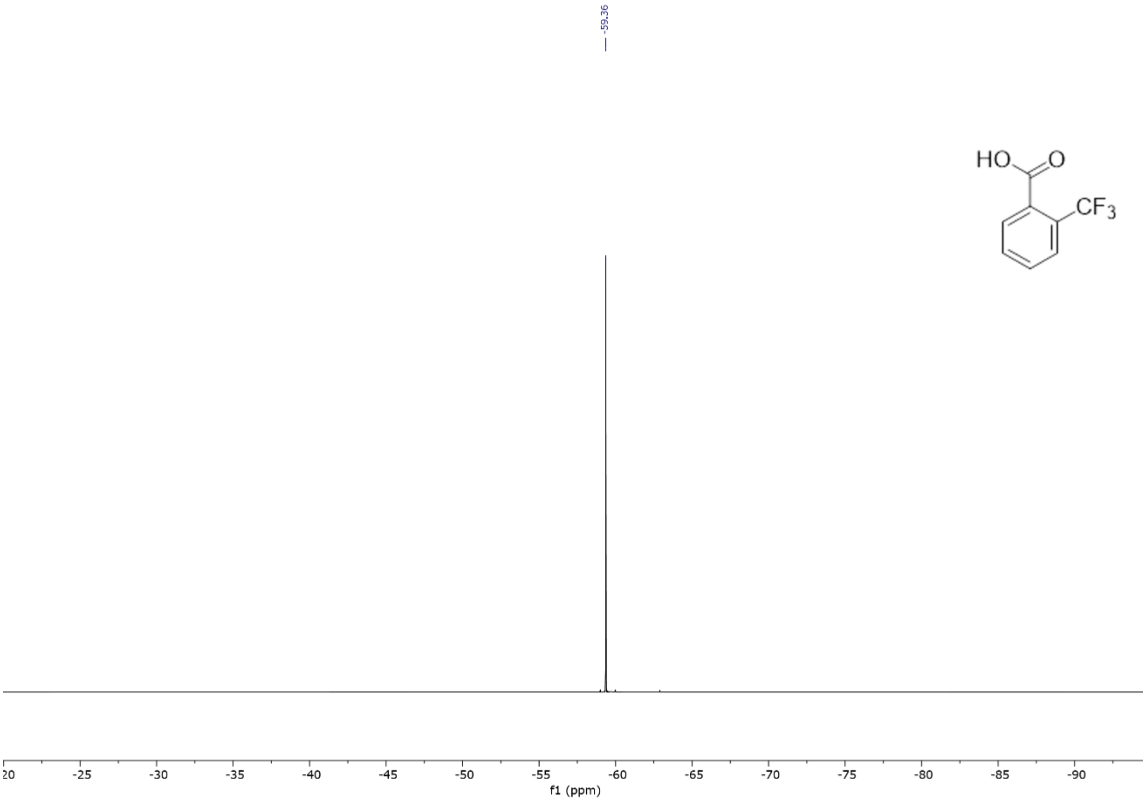

*Figure S30 $^{19}$F NMR of **2b** in $CDCl_3$.*

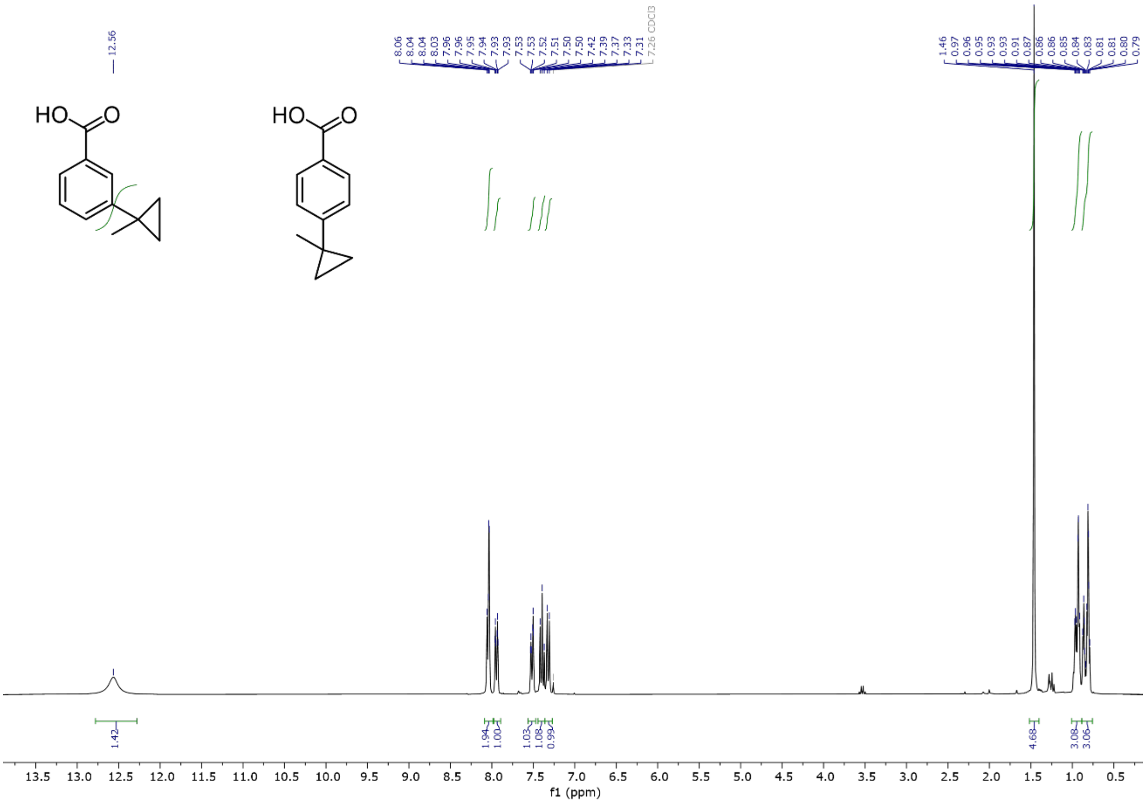

*Figure S31 $^{1}$H NMR of **2c** in $CDCl_3$.*

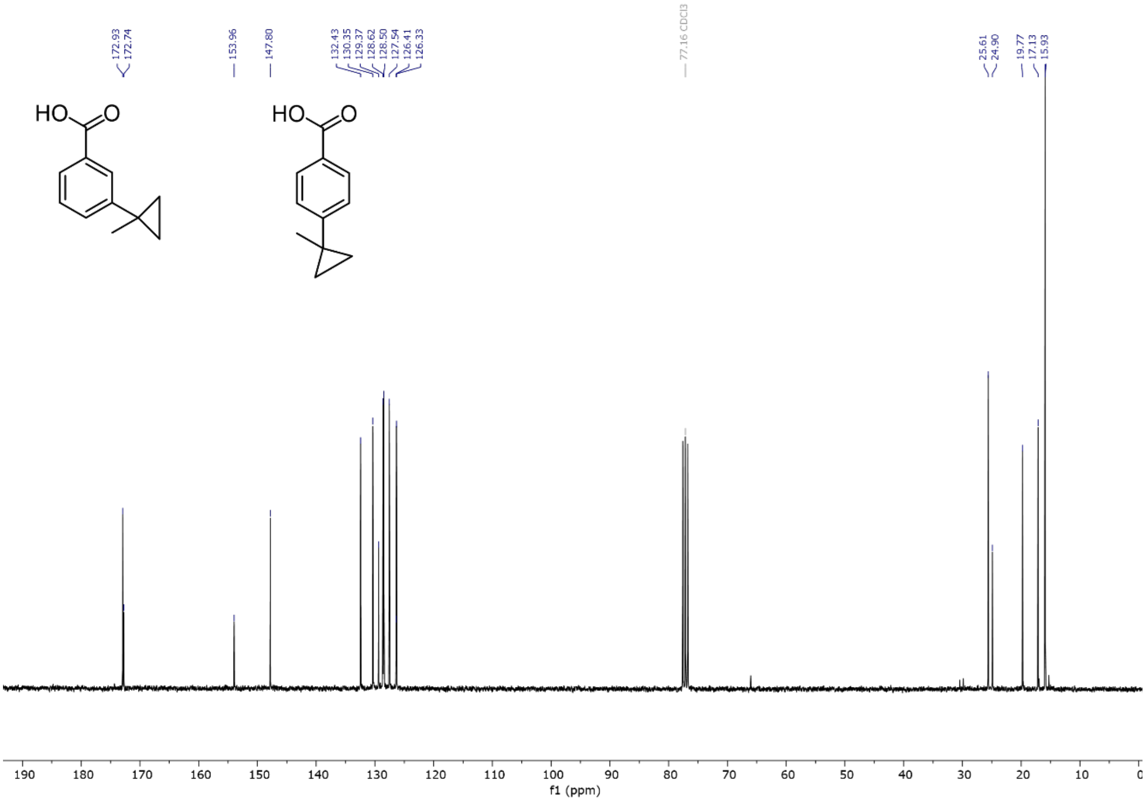


*Figure S32 $^{13}$C NMR of **2c** in $CDCl_3$.*

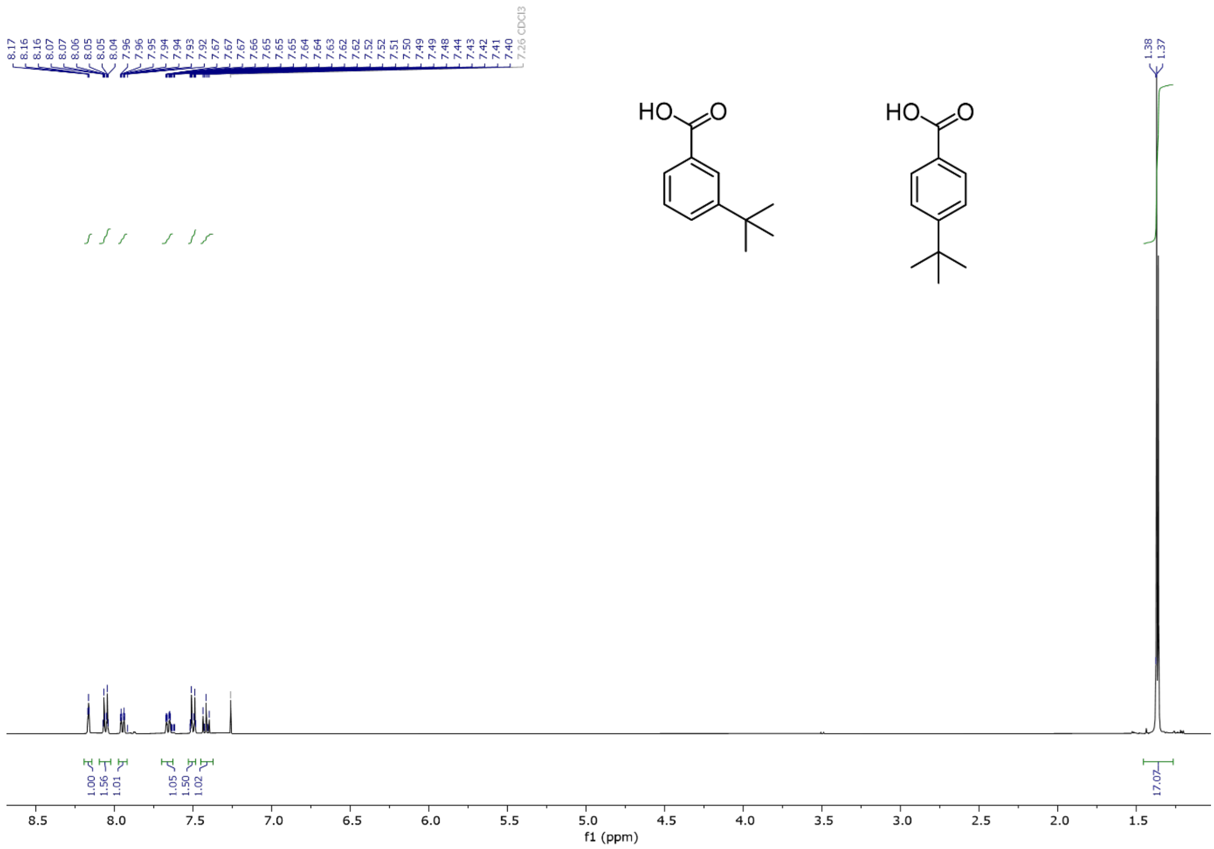

Figure S33 $^{1}H$ NMR of **2d** in $CDCl_3$.

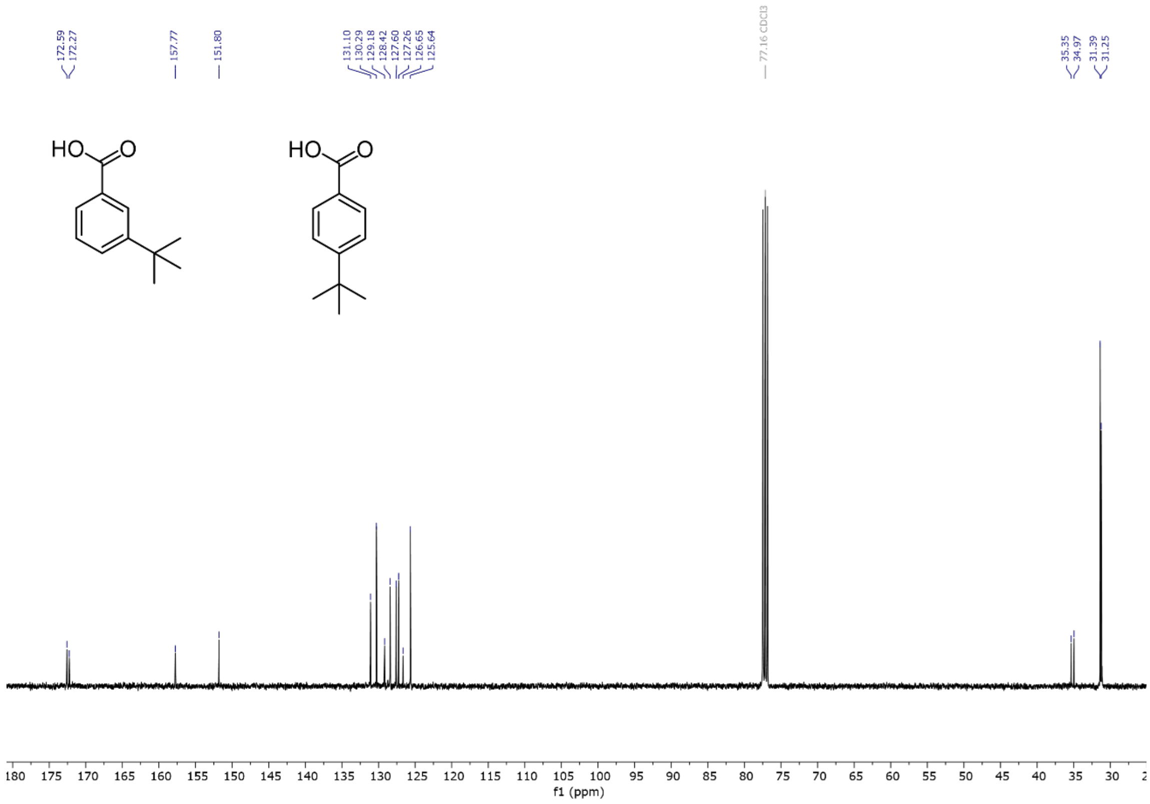

Figure S34 $^{13}C$ NMR of **2d** in $CDCl_3$.

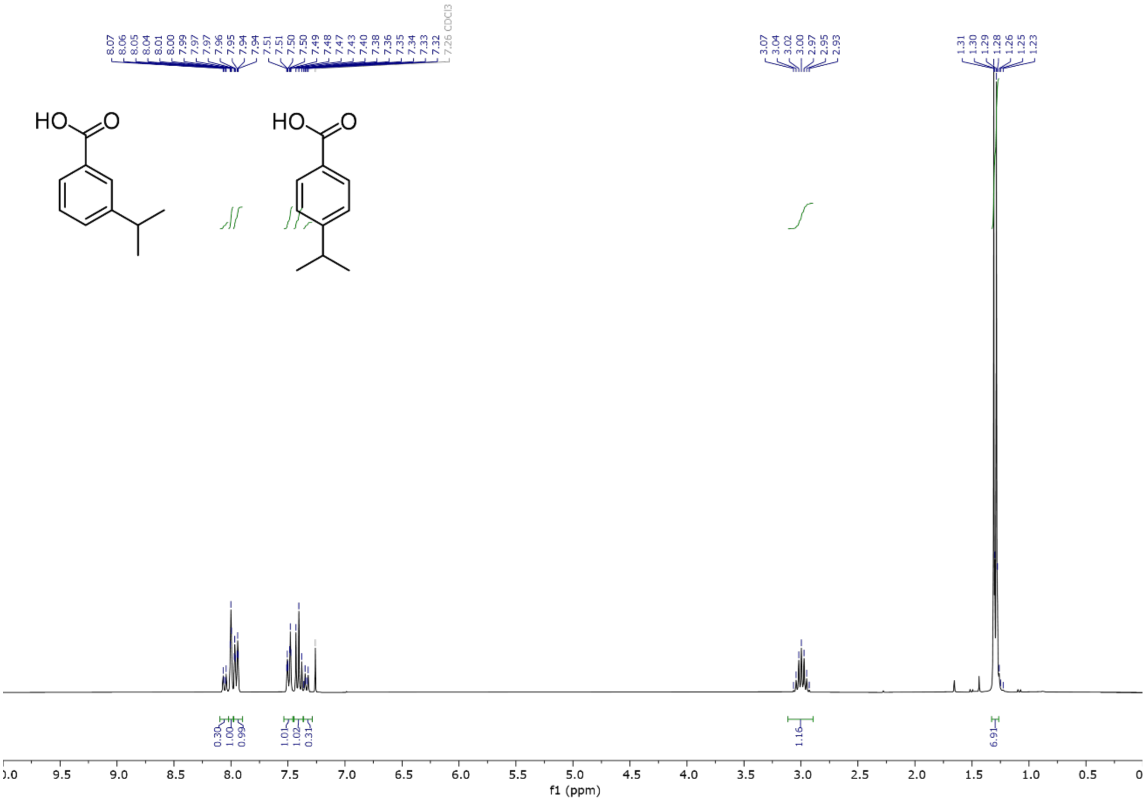

*Figure S35 $^{1}H$ NMR of **2e** in $CDCl_3$.*

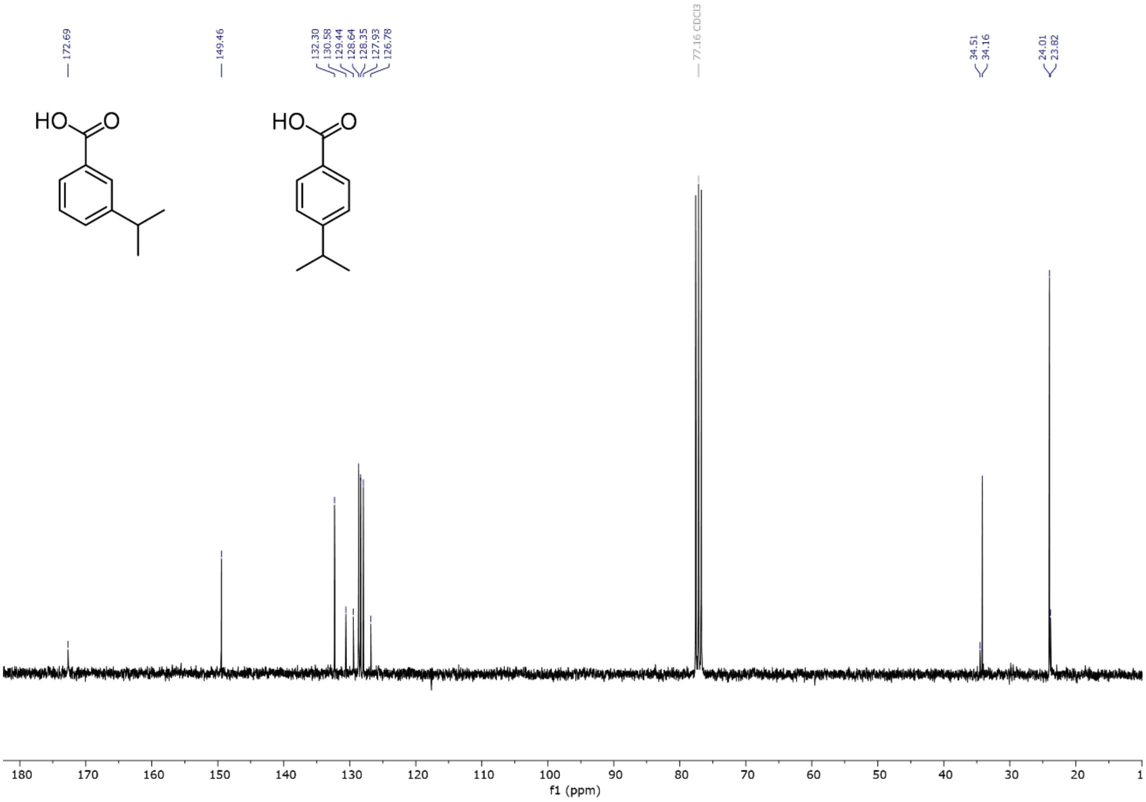

*Figure S36 $^{13}C$ NMR of **2e** in $CDCl_3$.*

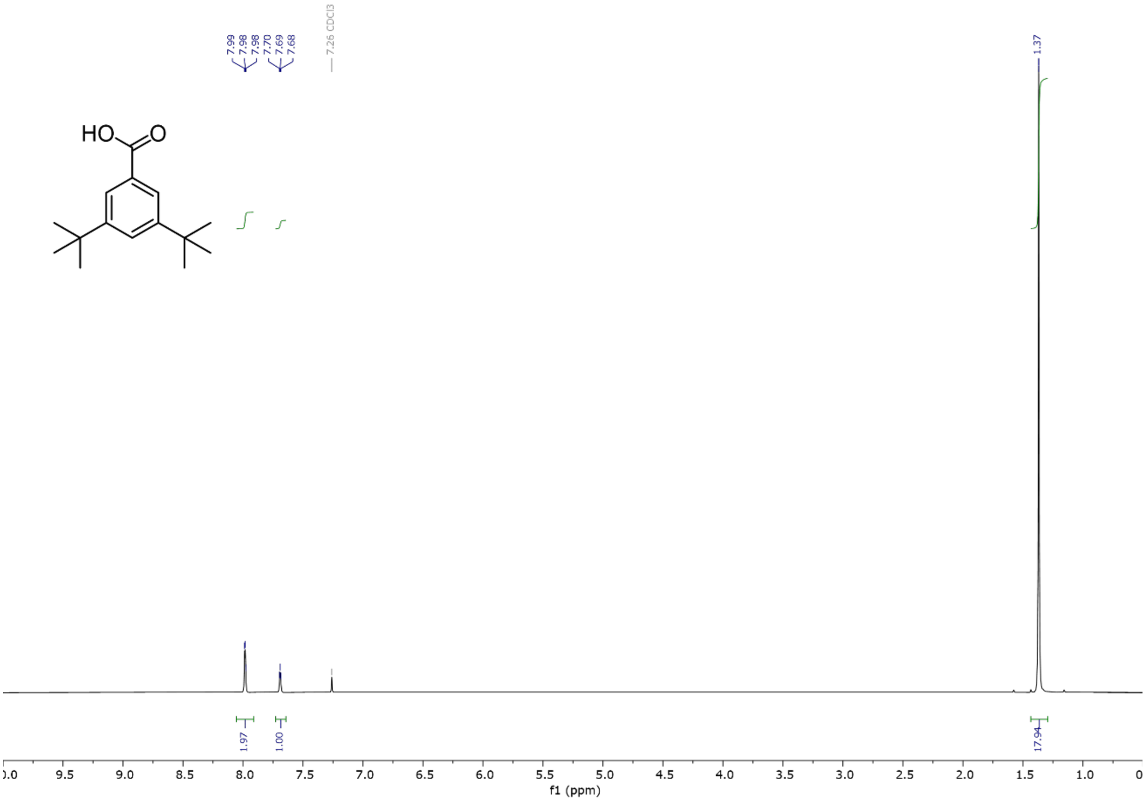


*Figure S37 $^{1}$H NMR of **2f** in $CDCl_3$.*

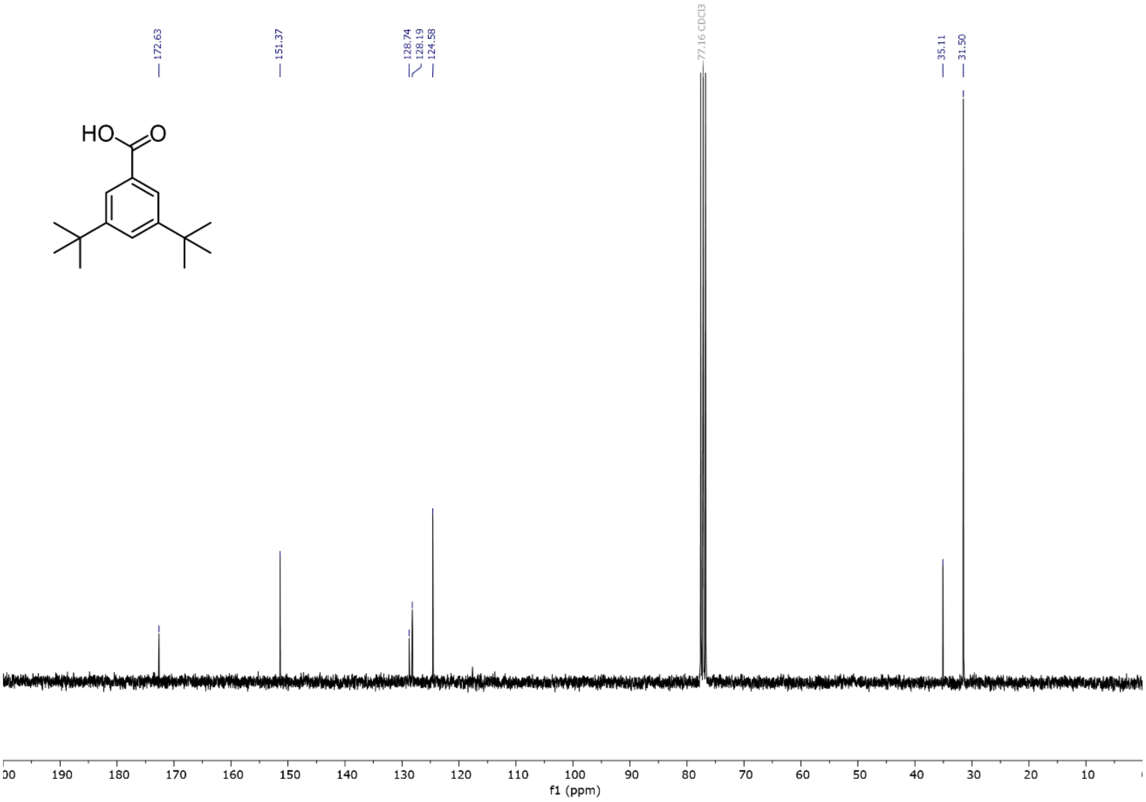


*Figure S38 $^{13}$C NMR of **2f** in $CDCl_3$.*

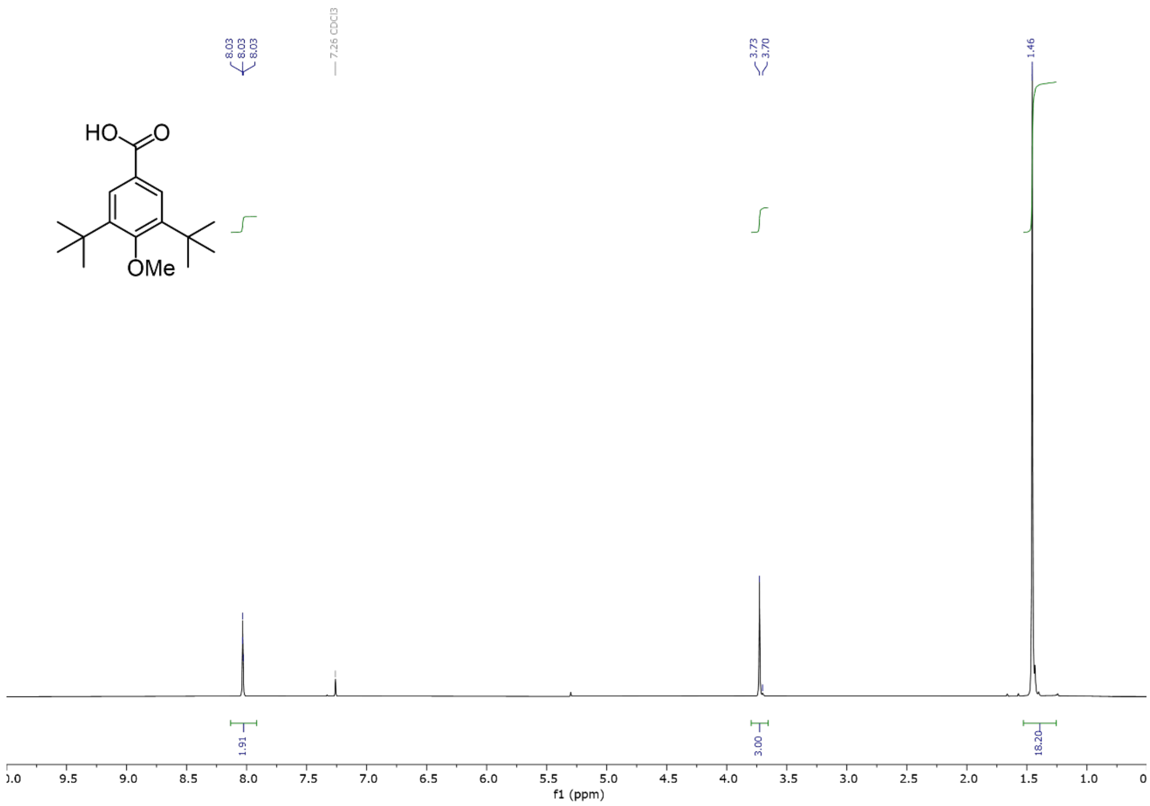


*Figure S39 [1]H NMR of **2g** in $CDCl_3$.*

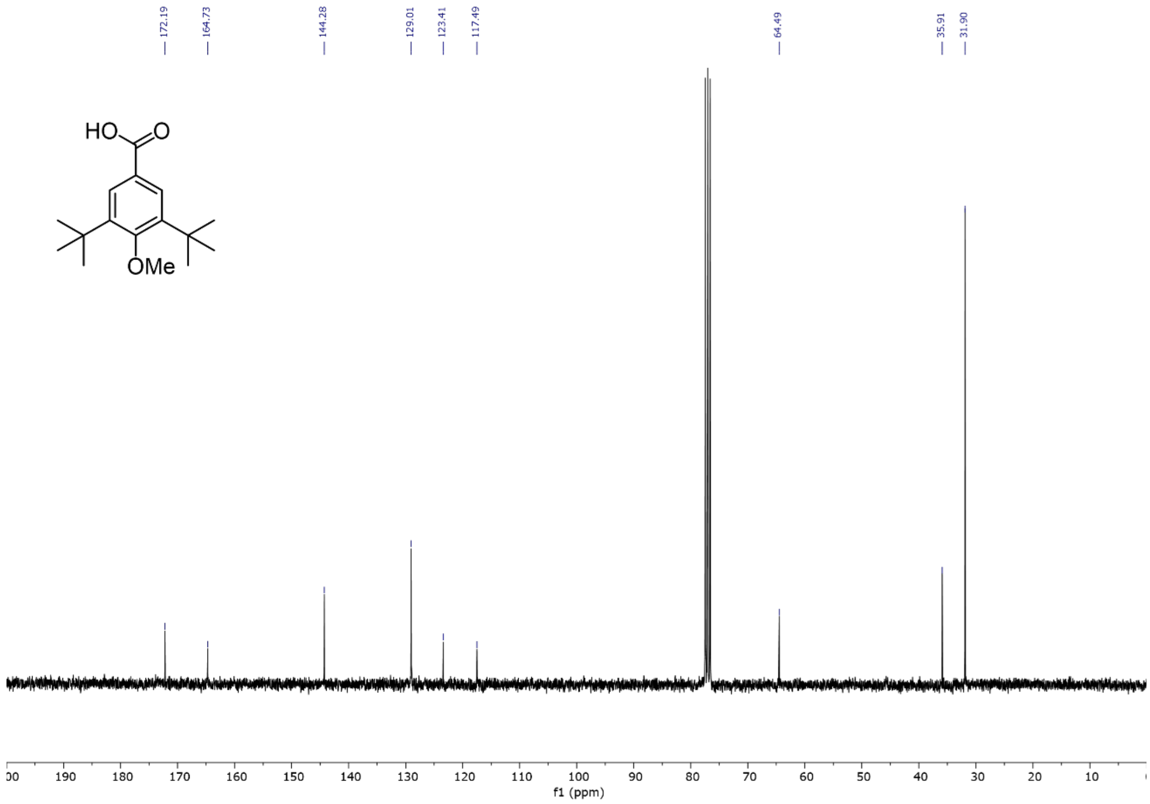


*Figure S40 [13]C NMR of **2g** in $CDCl_3$.*

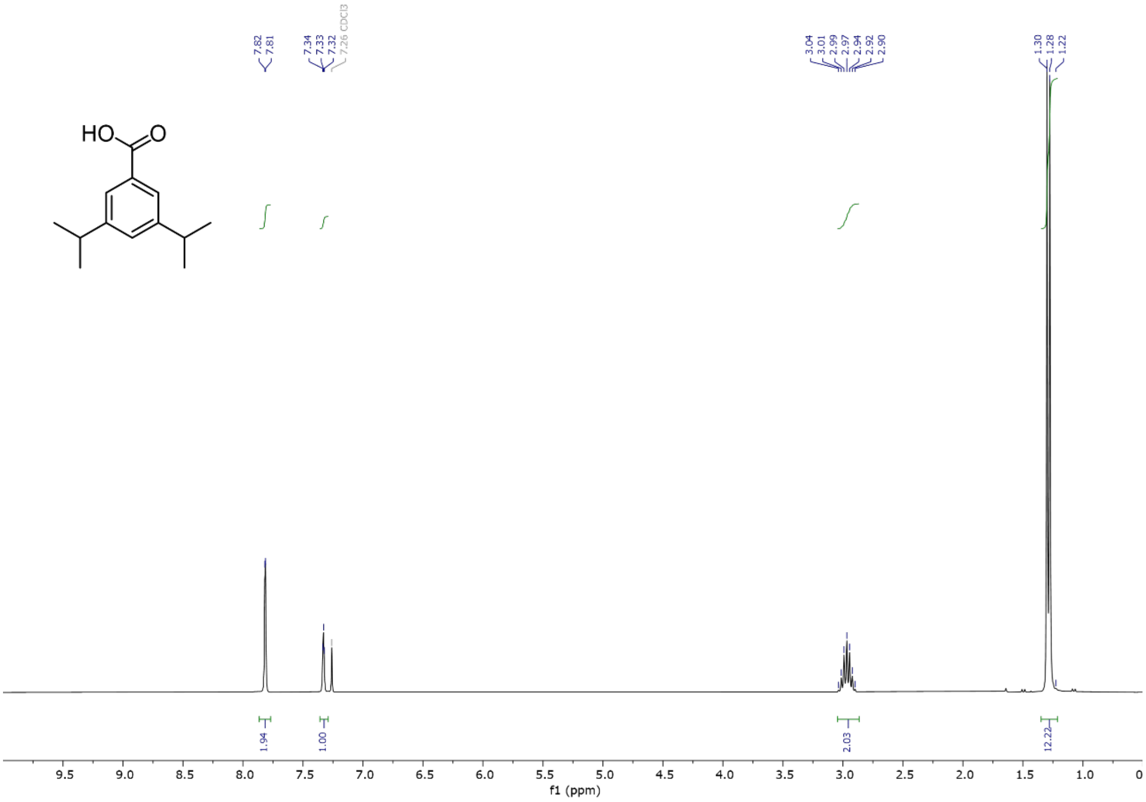


*Figure S41* [1]*H NMR of* ***2h*** *in* $CDCl_3$.

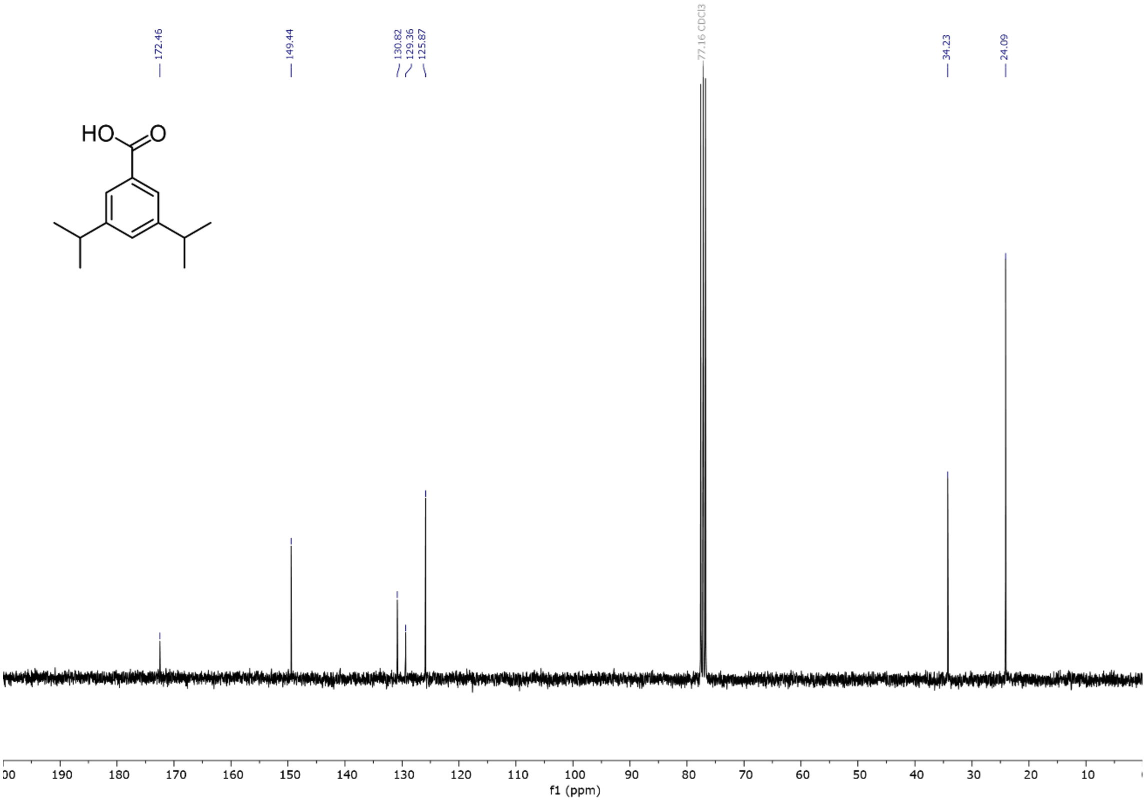


*Figure S42* [13]*C NMR of* ***2h*** *in* $CDCl_3$.

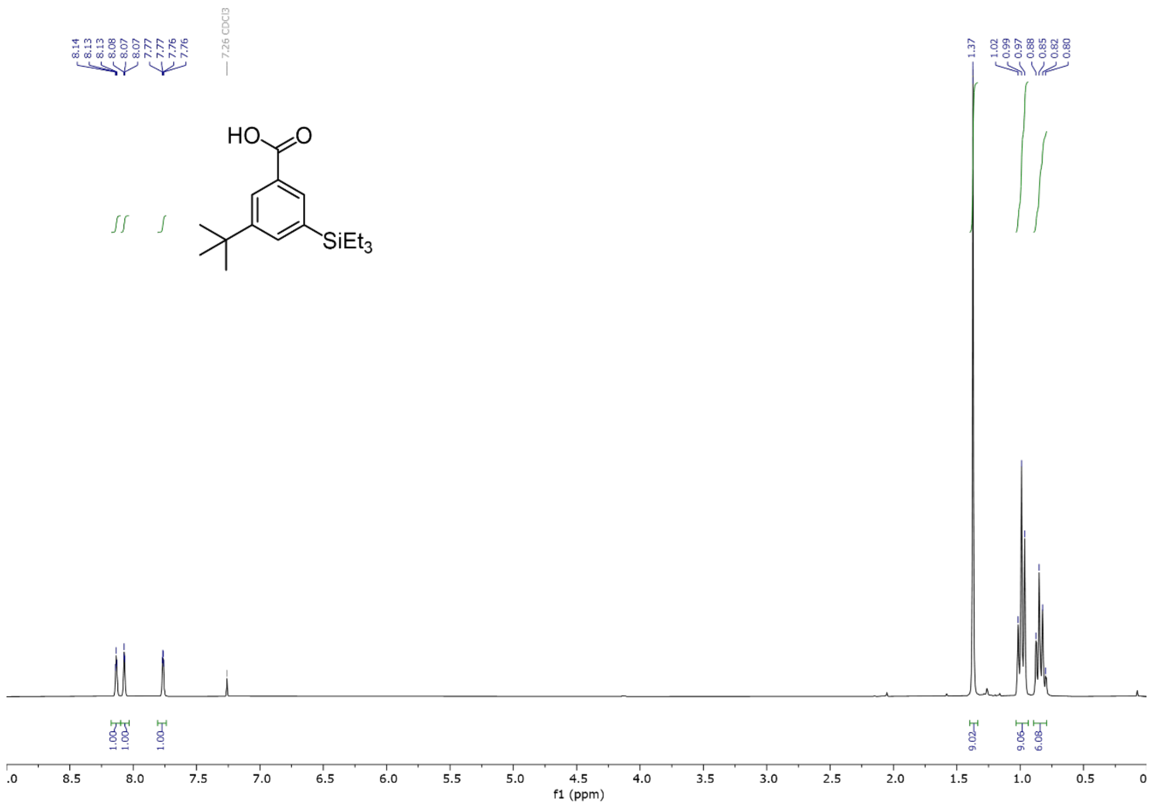

*Figure S43 ¹H NMR of **2i** in $CDCl_3$.*

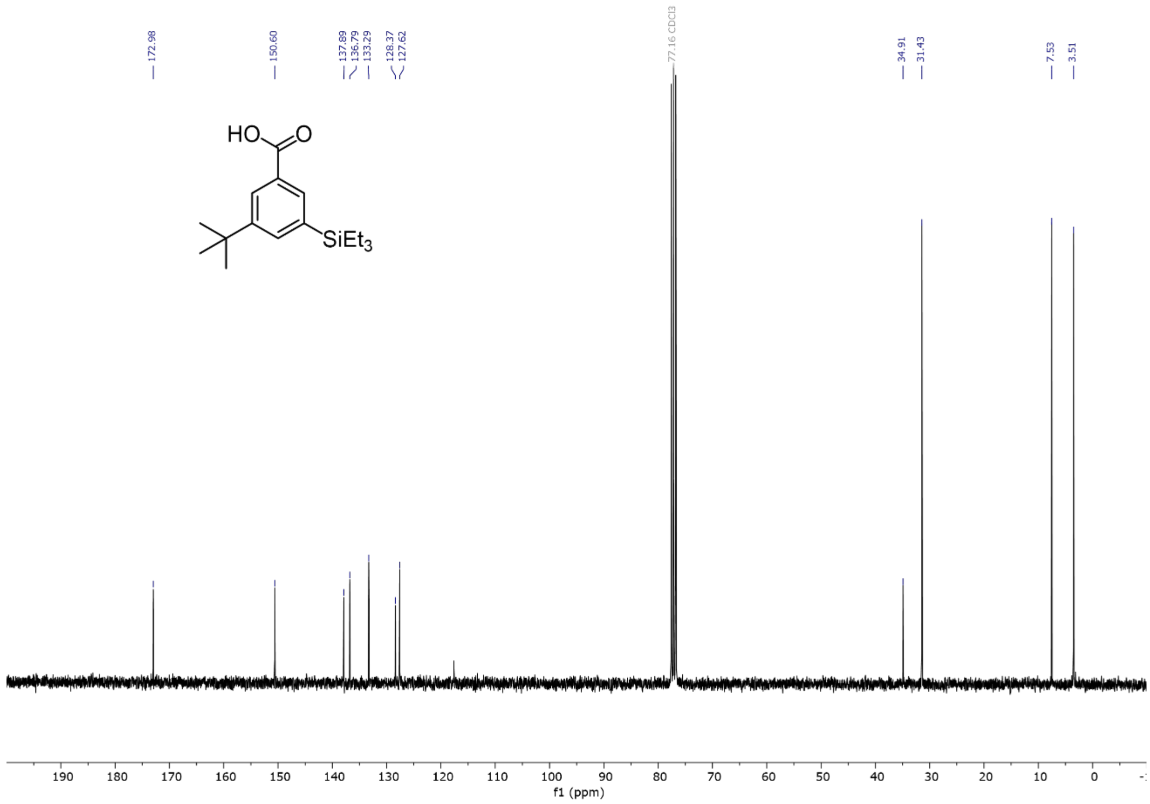

*Figure S44 ¹³C NMR of **2i** in $CDCl_3$.*

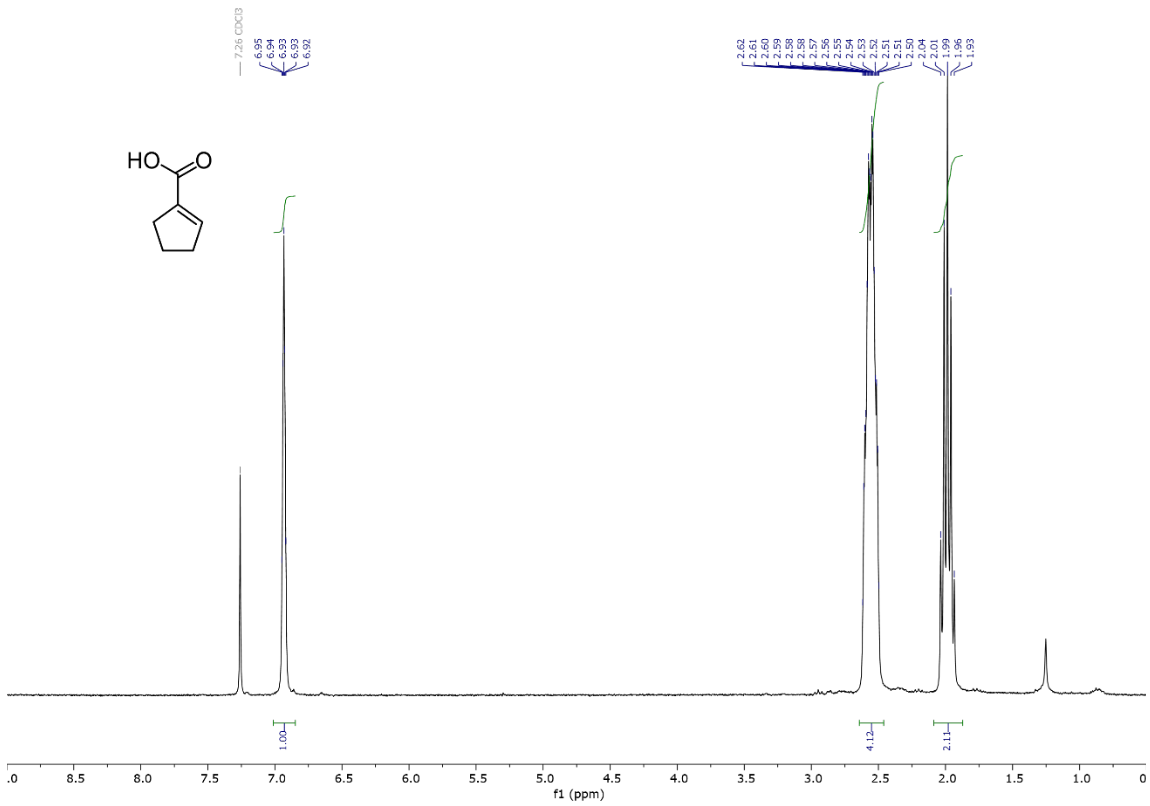

*Figure S45 $^1$H NMR of **2j** in $CDCl_3$.*

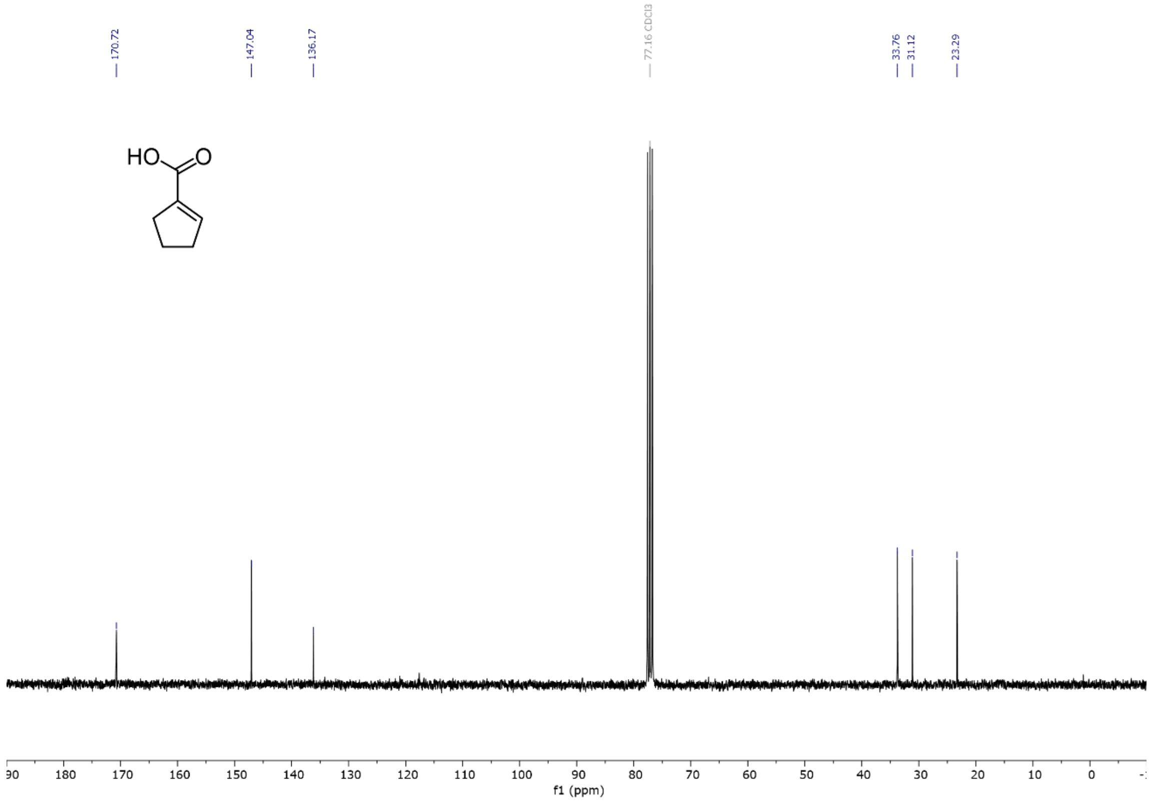

*Figure S46 $^{13}$C NMR of **2j** in $CDCl_3$.*

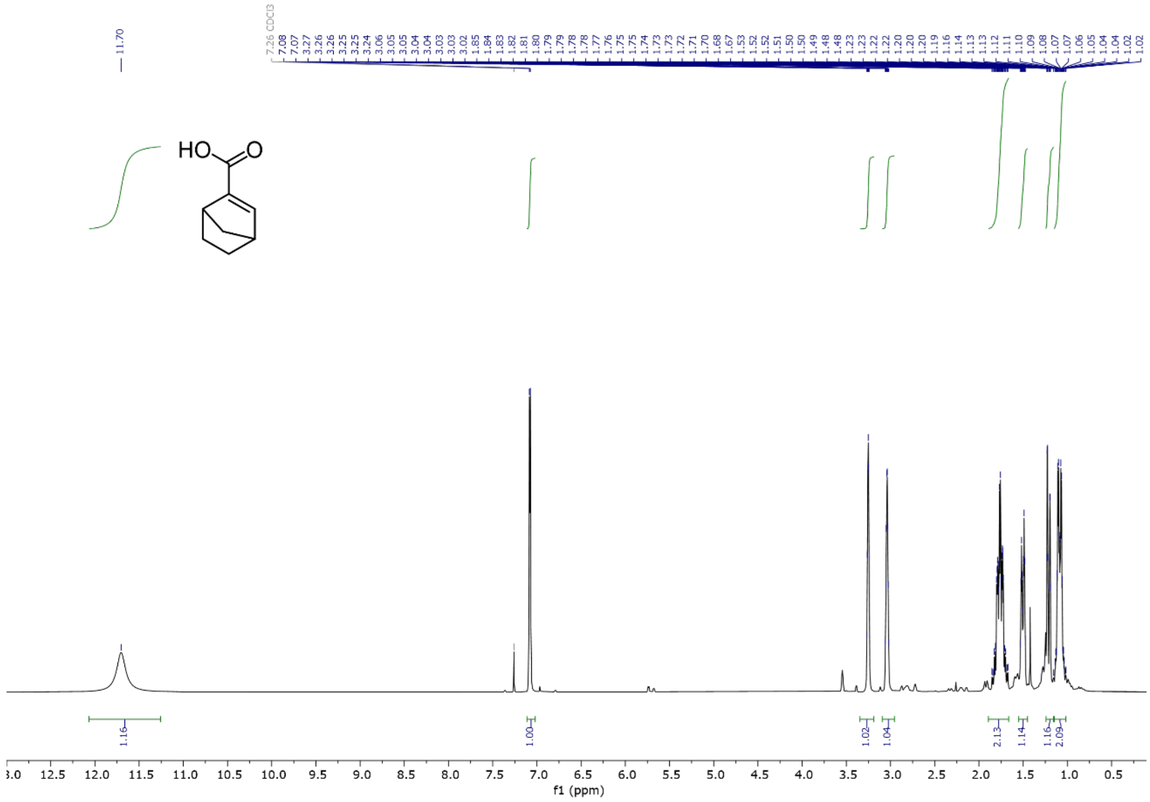

*Figure S47 $^{1}H$ NMR of **2k** in $CDCl_3$.*

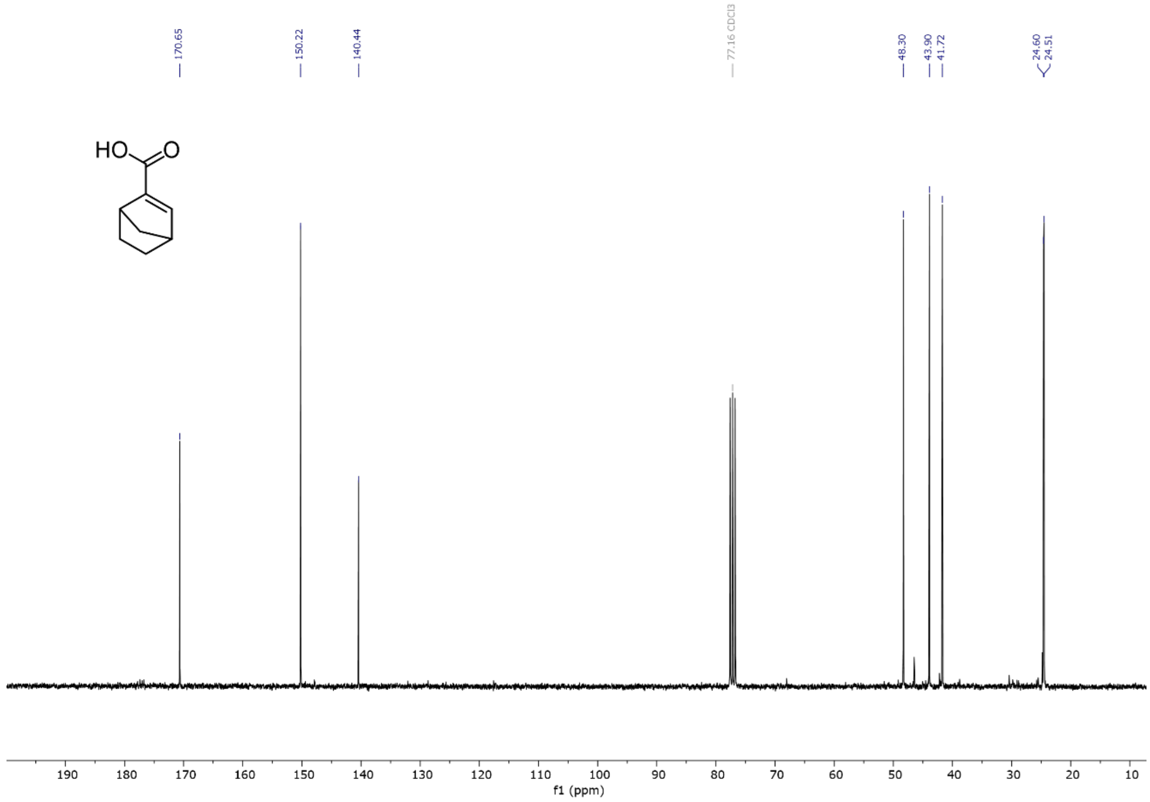

*Figure S48 $^{13}C$ NMR of **2k** in $CDCl_3$.*

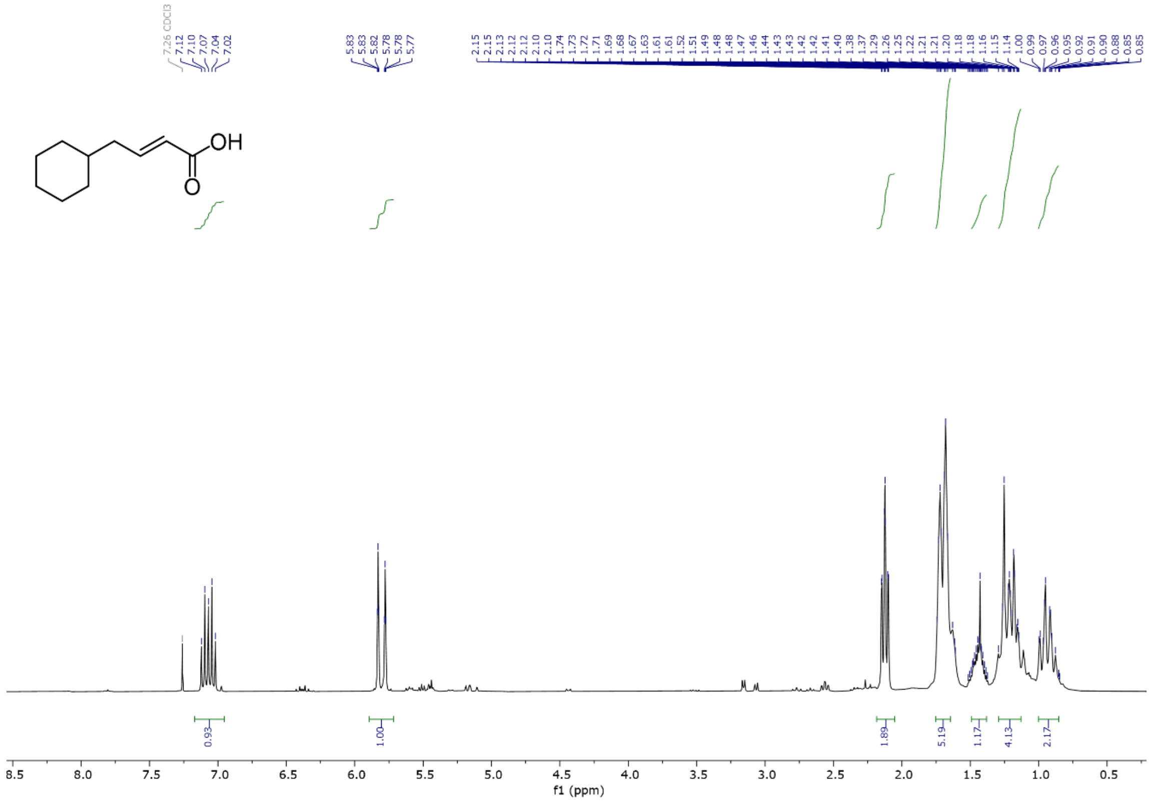


*Figure S49* $^{1}H$ *NMR of* ***2l*** *in* $CDCl_3$.

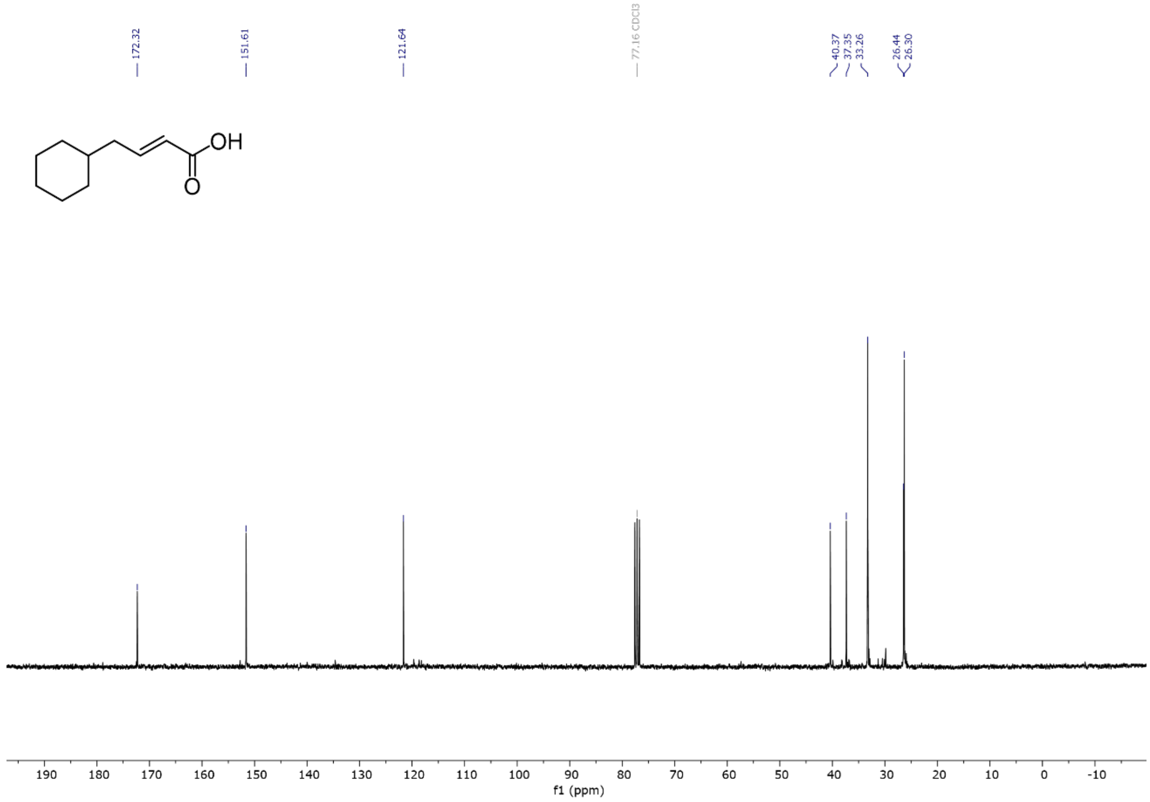


*Figure S50* $^{13}C$ *NMR of* ***2l*** *in* $CDCl_3$.

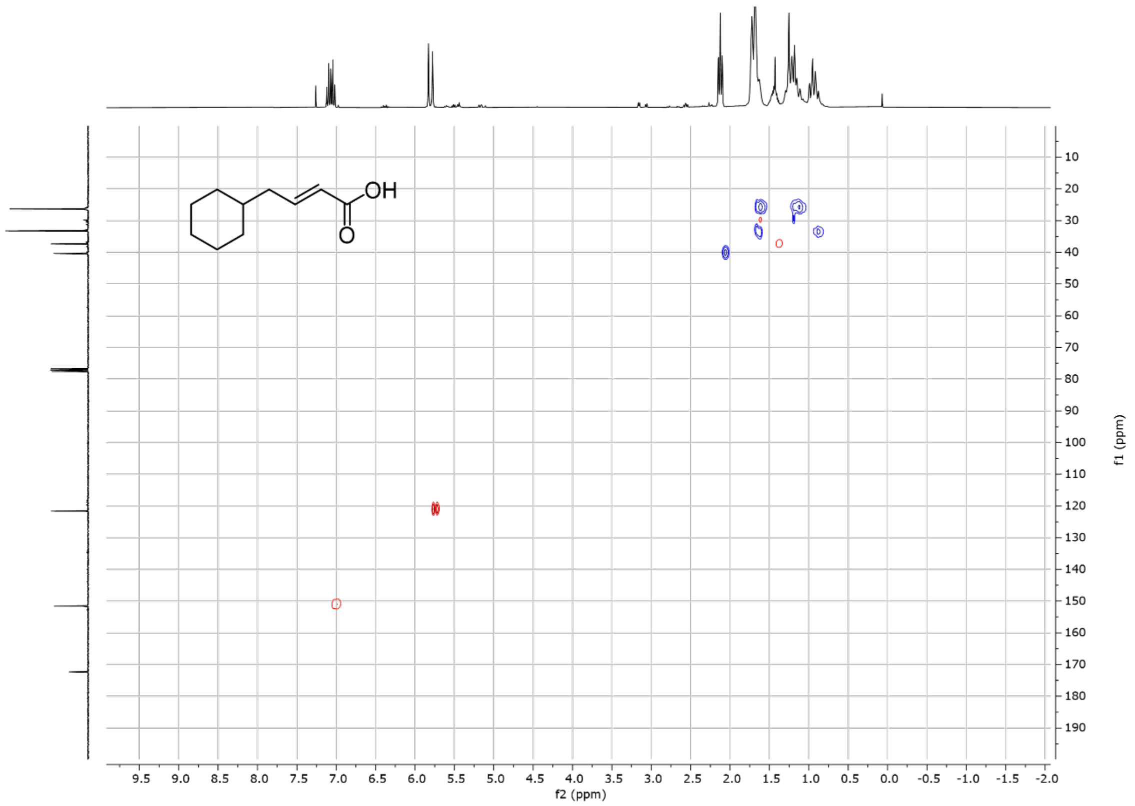


*Figure S51 $^1$H-$^{13}$C HSQC NMR of **2l** in $CDCl_3$.*

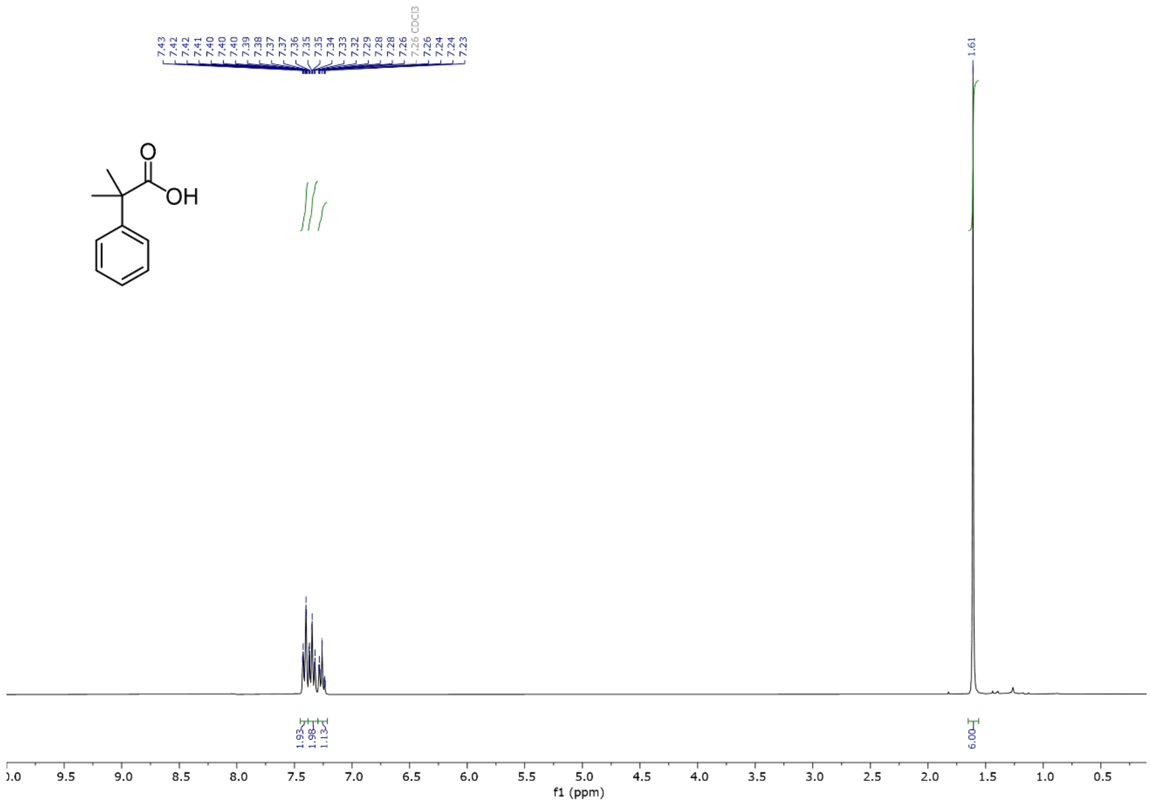


*Figure S52 $^1$H NMR of **2m** in $CDCl_3$.*

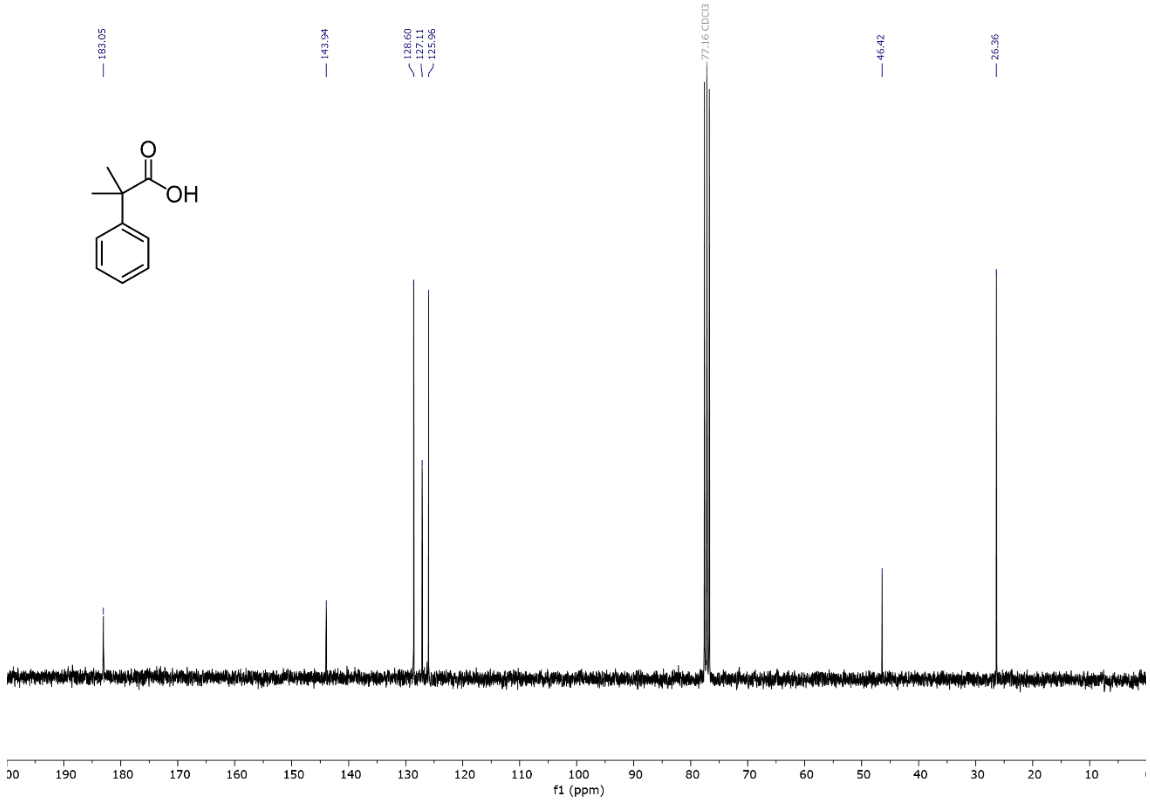

*Figure S53 $^{13}C$ NMR of **2m** in $CDCl_3$.*

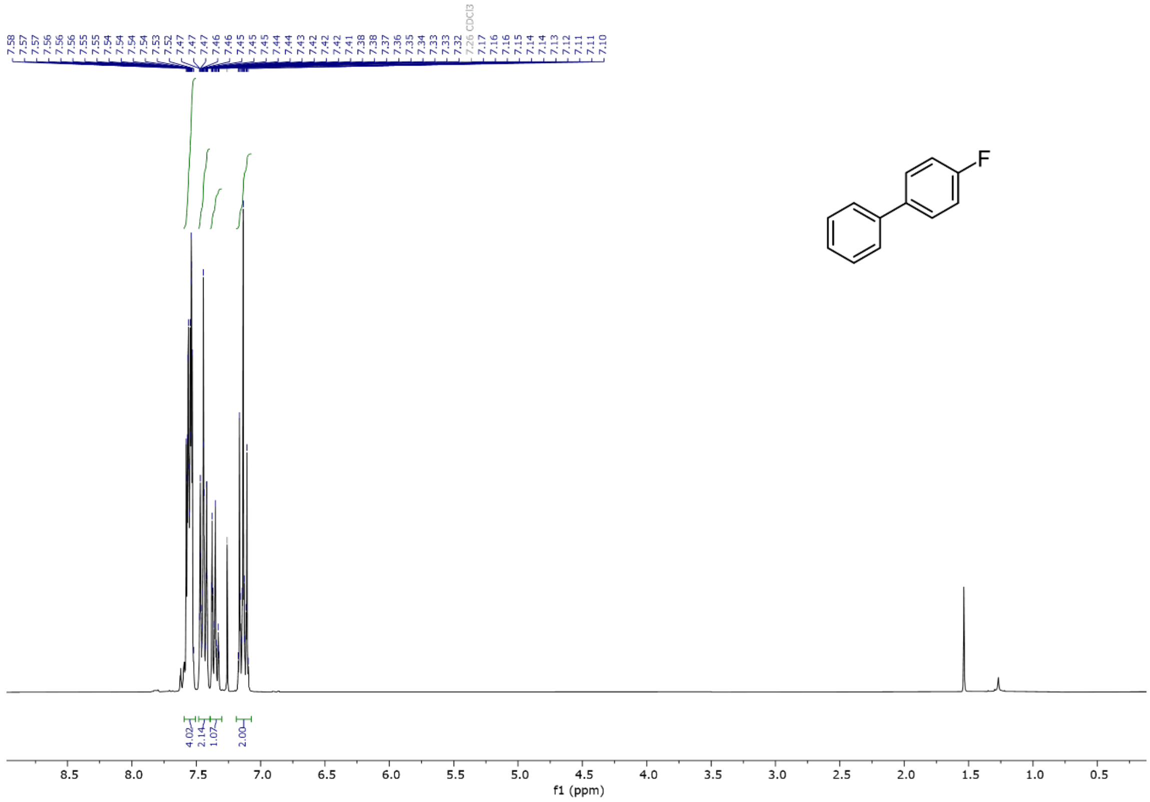

*Figure S54 $^{1}H$ NMR of **5a** in $CDCl_3$.*

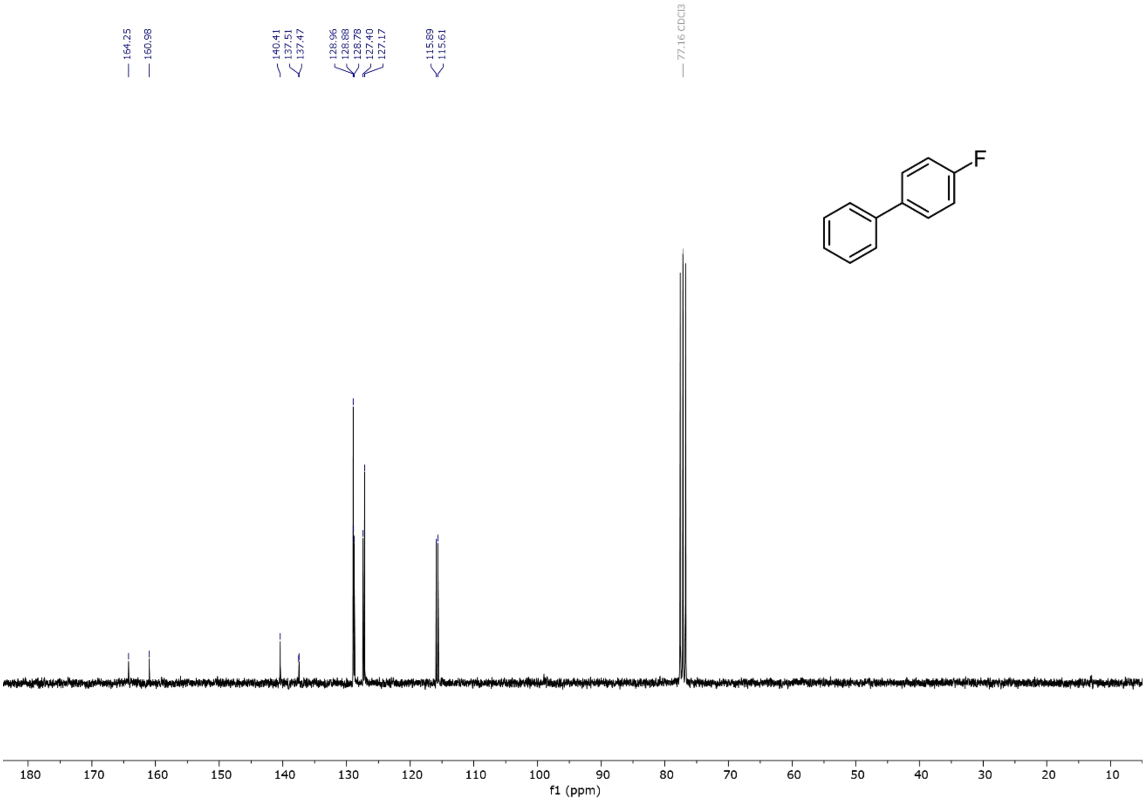


*Figure S55 $^{19}$F NMR of* ***5a*** *in $CDCl_3$.*

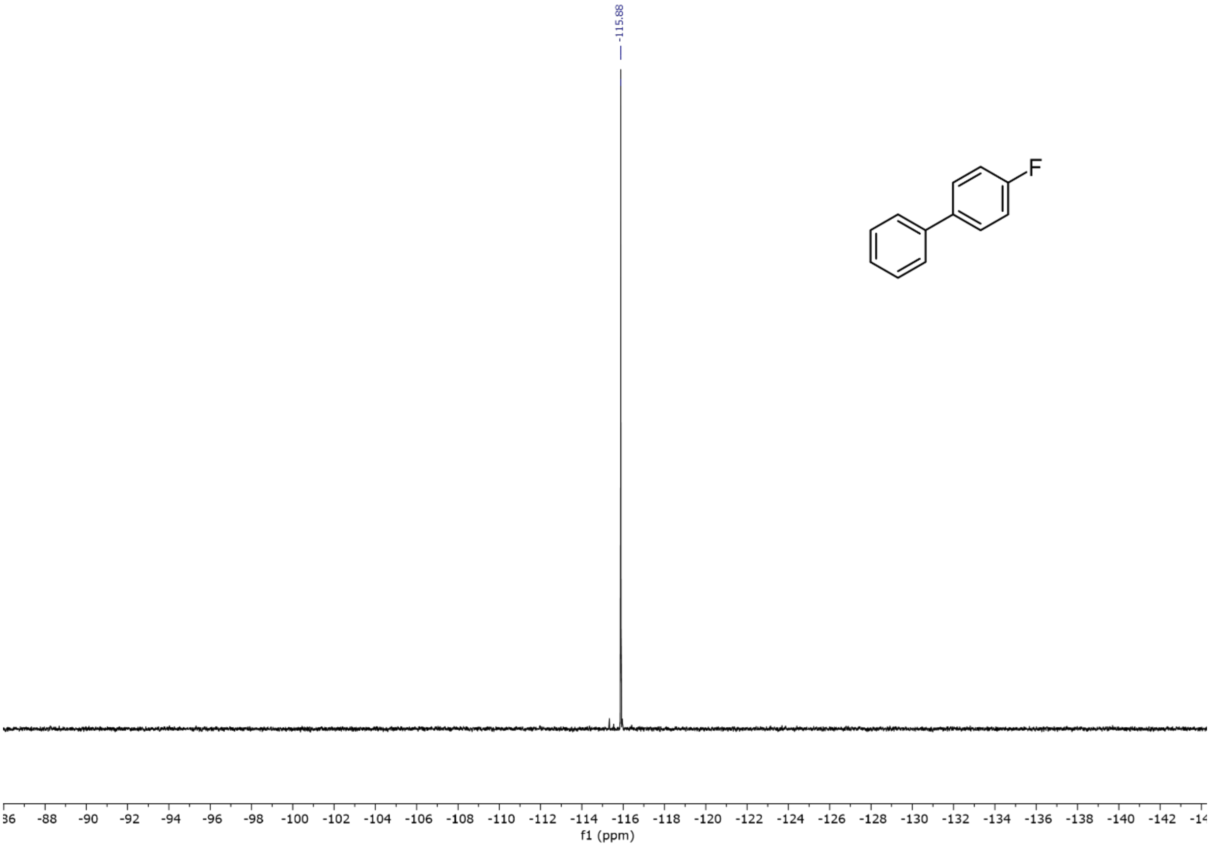


*Figure S56 $^{19}$F NMR of* ***5a*** *in $CDCl_3$.*

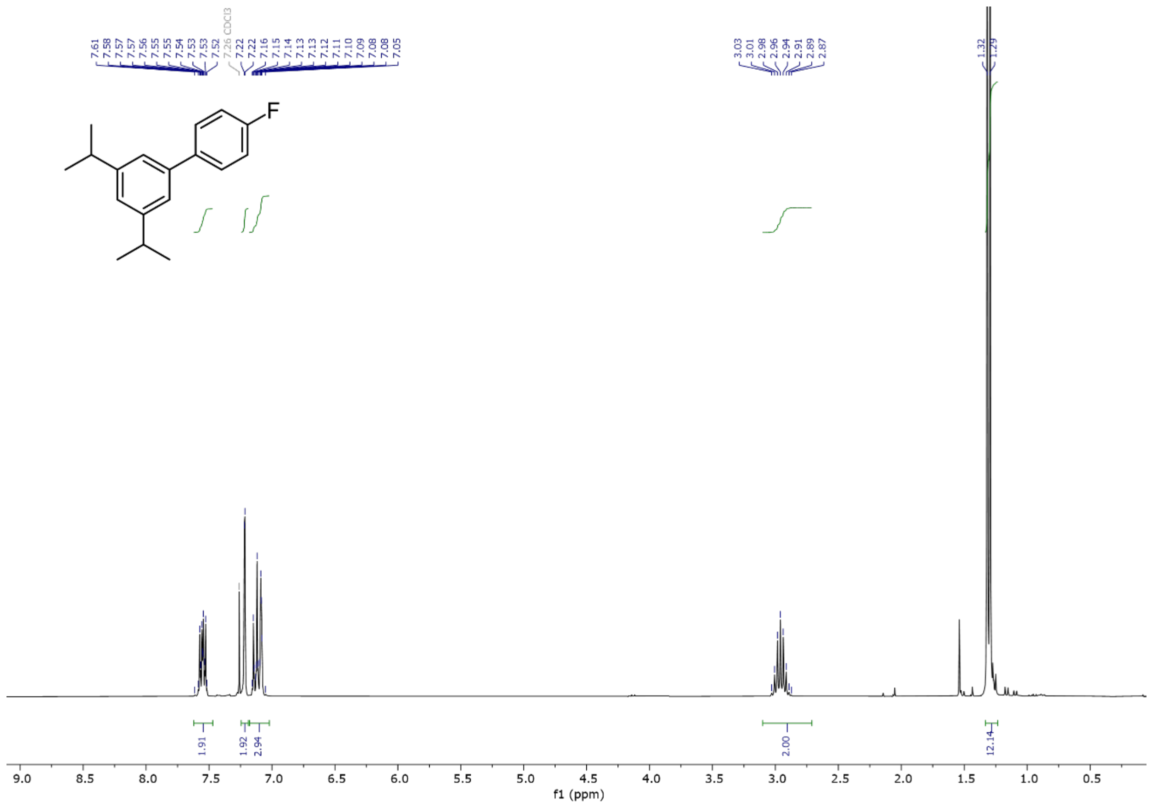

*Figure S57 $^{1}H$ NMR of **5b** in $CDCl_3$.*

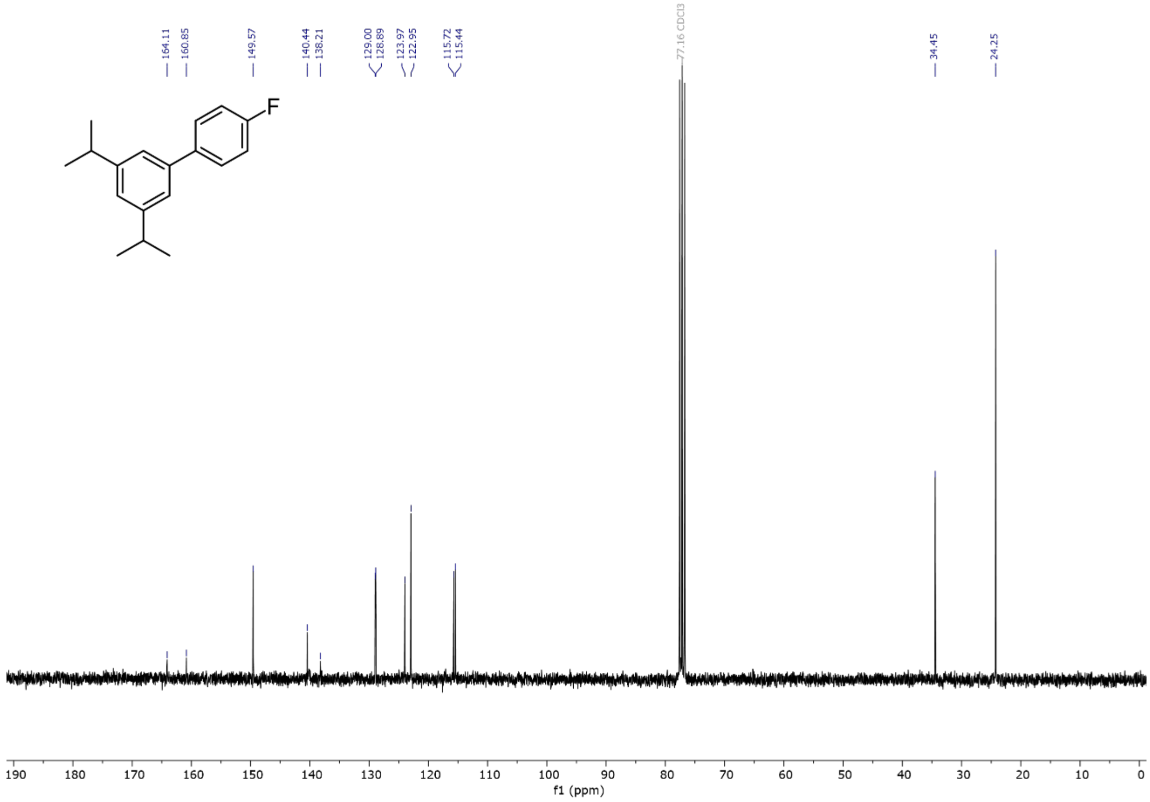

*Figure S58 $^{13}C$ NMR of **5b** in $CDCl_3$.*

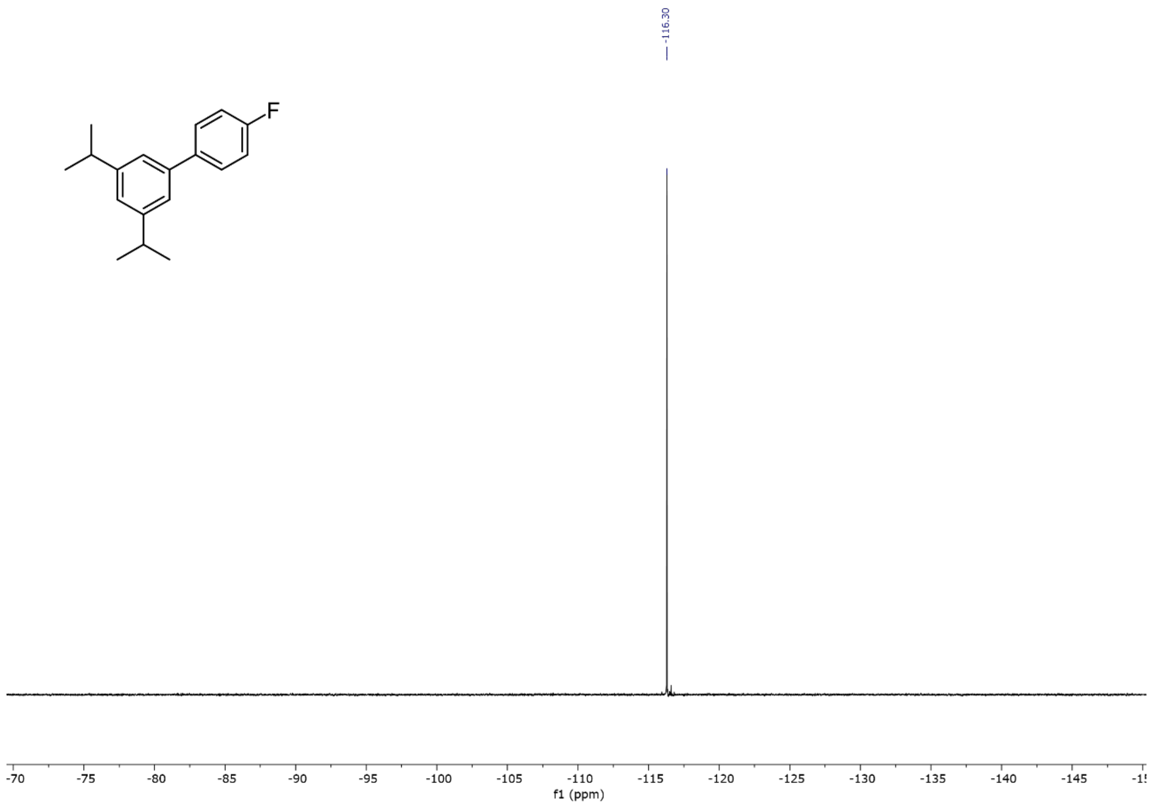

*Figure S59 $^{19}$F NMR of **5b** in $CDCl_3$.*

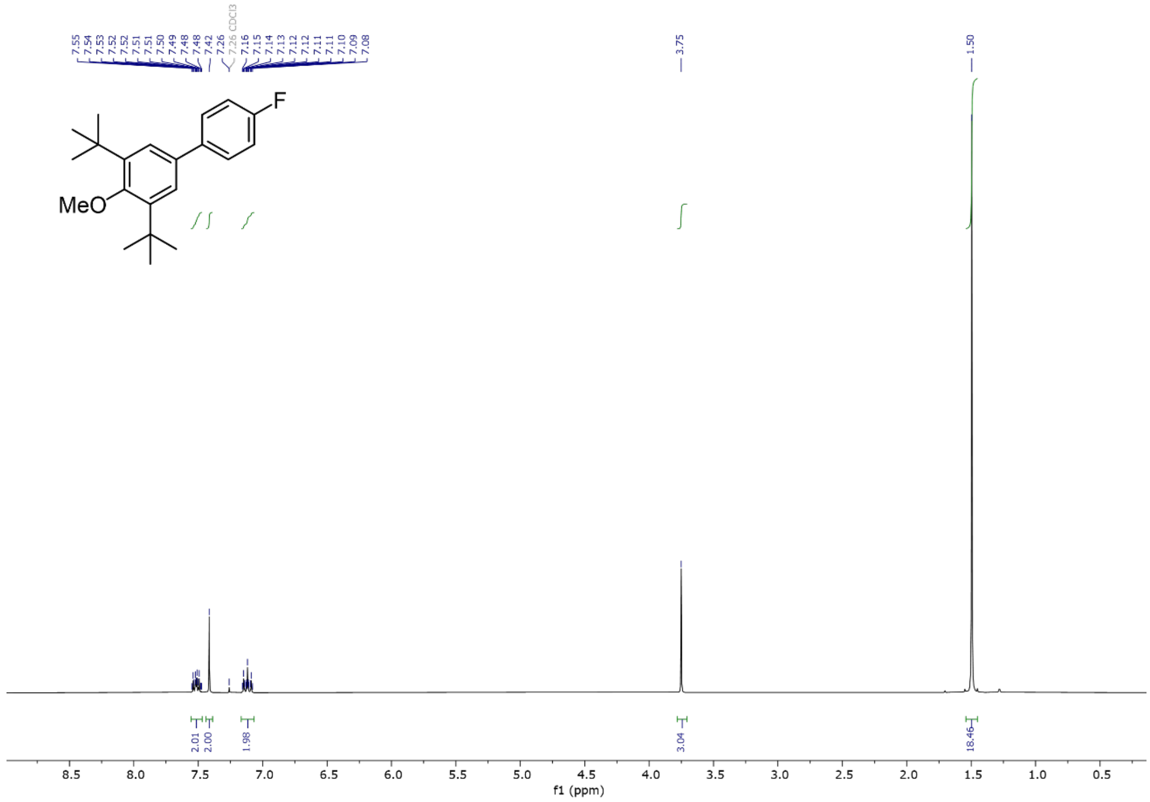

*Figure S60 $^{1}$H NMR of **5c** in $CDCl_3$.*

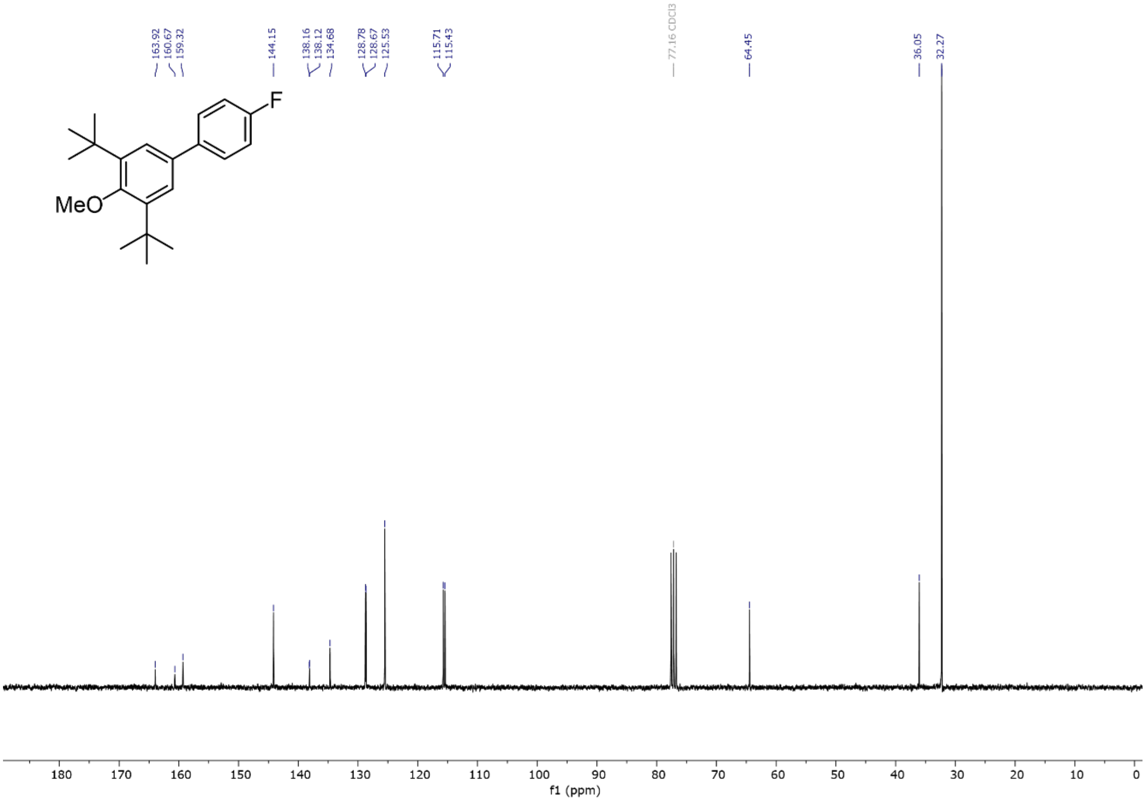


*Figure S61[13]C NMR of **5c** in $CDCl_3$.*

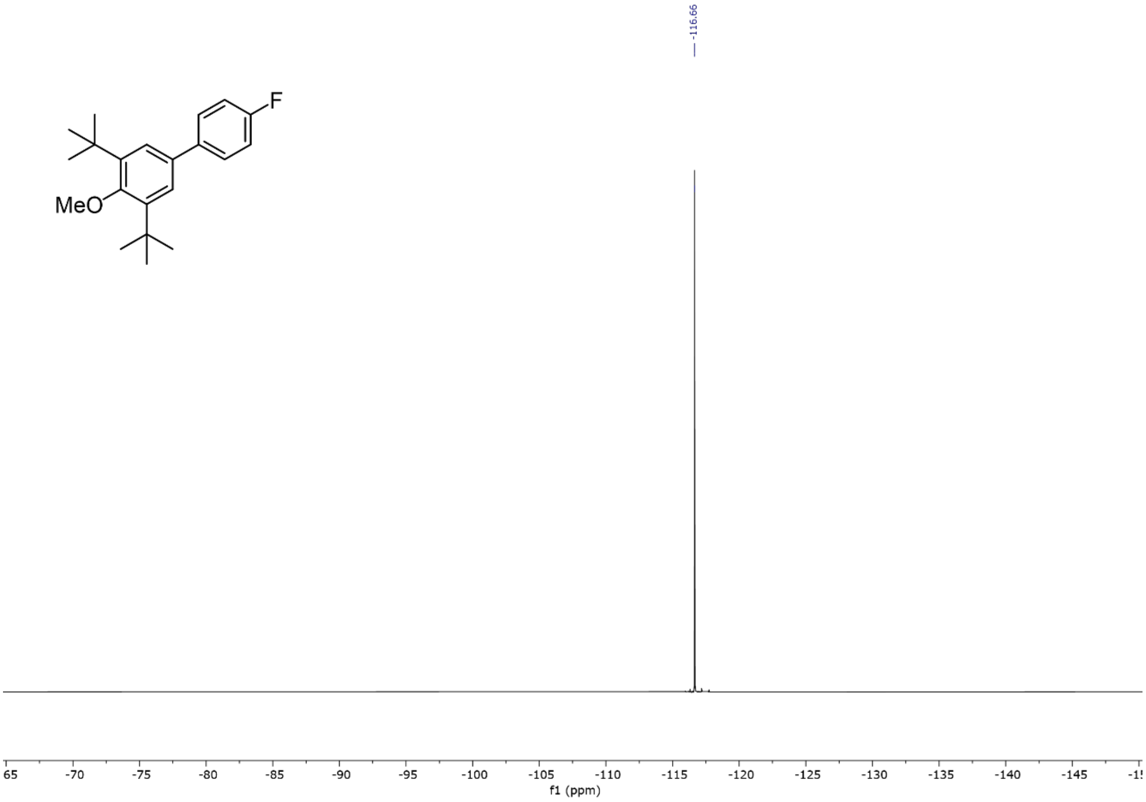


*Figure S62 [13]C NMR of **5c** in $CDCl_3$.*

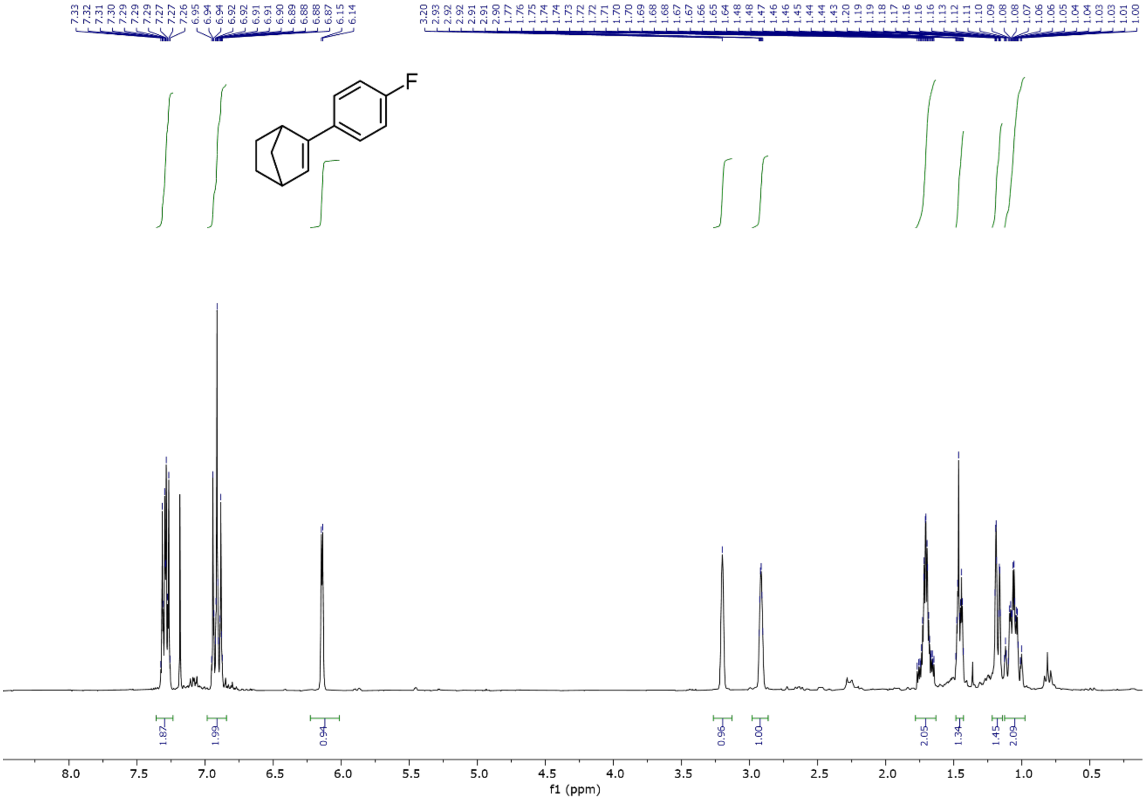

Figure S63 $^{1}H$ NMR of **5d** in $CDCl_3$.

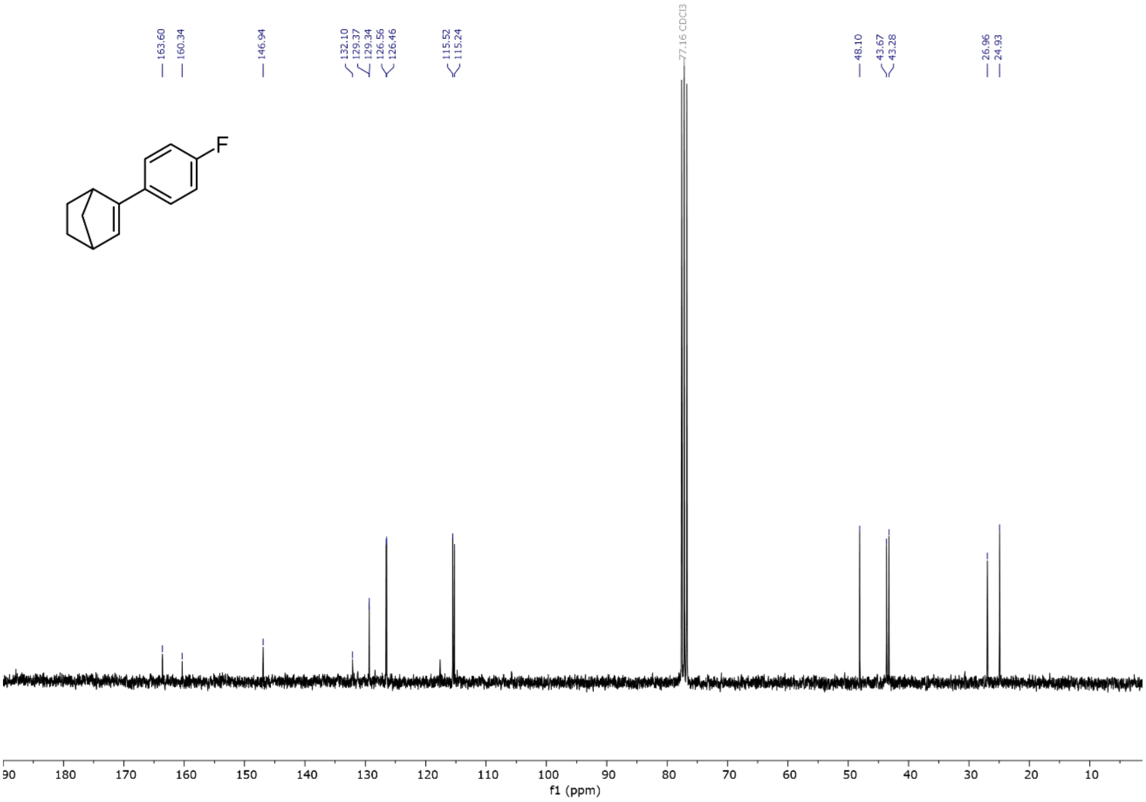

Figure S64 $^{13}C$ NMR of **5d** in $CDCl_3$.

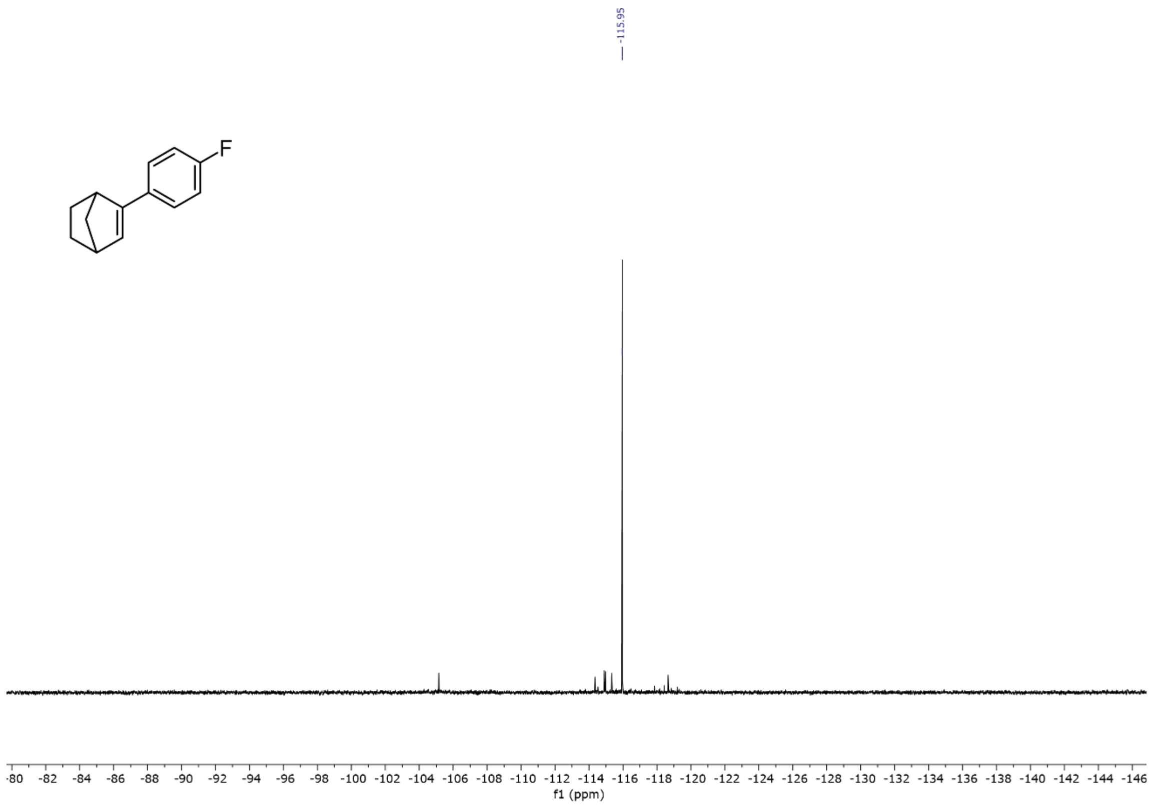


*Figure S65* $^{19}$*F NMR of* ***5d*** *in* $CDCl_3$.

# Sodium neo-pentenyl, theoretical analysis

### Computational part

Isolated-molecule geometry optimizations of the *(dimer)* and the *(tetramer)* were carried out with the software Gaussian09,[18] starting from the respective geometries of the crystal structures. As level of theory, B3LYP/def2-TZVP was chosen, once without and once with empirical dispersion energy correction according to the GD3BJ scheme.[19] Subsequently, frequency analyses were done to ensure that the resulting geometries are minima on the potential-energy surface. For the *(dimer)* with GD3BJ correction, two imaginary frequencies with small absolute values (14.4 and 2.4 $cm^{-1}$) were unavoidable. Their main trajectories of vibration correspond to the experimentally observed dynamic disorder of the TMEDA groups. Relative energies between calculations with and without empirical dispersion correction include zero-point vibrational energy corrections.

A selection of different bonding descriptors was derived from the optimized-geometry wavefunctions.[20] Results according to the Quantum Theory of Atoms in Molecules (QTAIM)[21] were calculated and displayed with the software AimAll.[22] The non-covalent interaction (NCI) index[23] was calculated with Multiwfn[24] and plotted with the software VMD.[25] The electron localizability indicator (ELI-D)[26] was derived with the program Dgrid [27] and visualized with Vesta.[28]

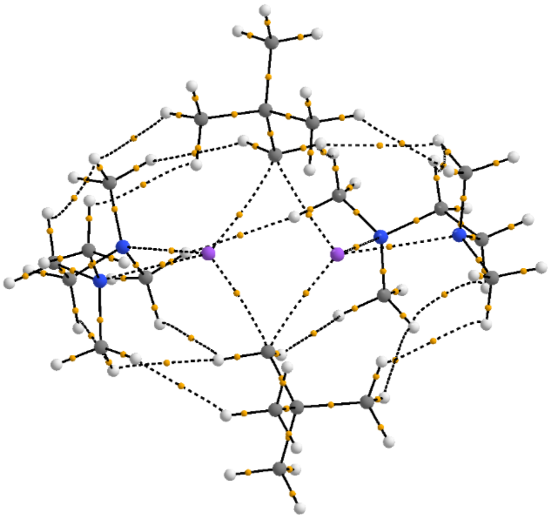

(a)

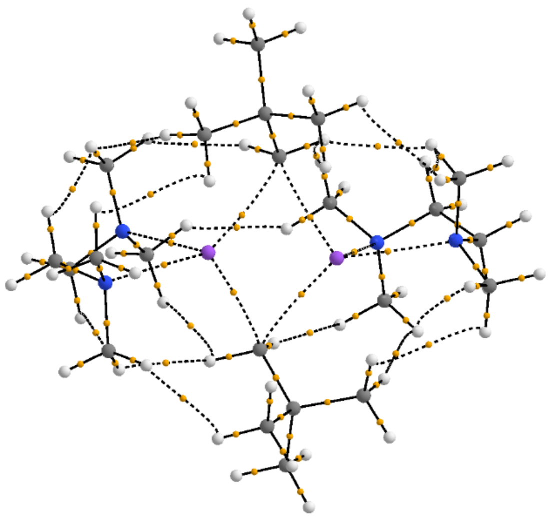

(b)

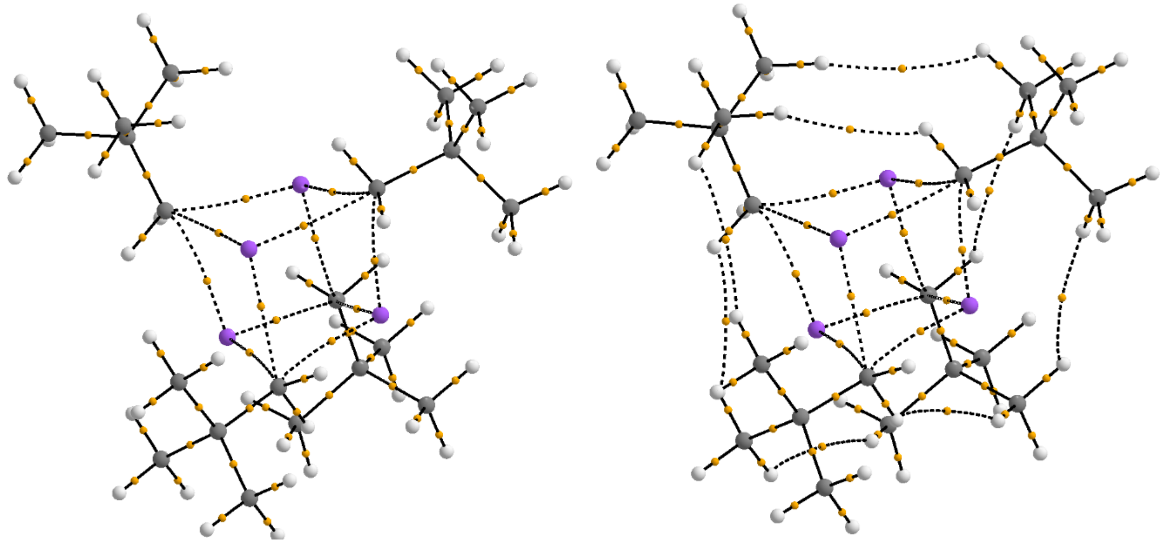

(c) (d)

Figure S66. (a) and (b) **1b**; (c) and (d) **1a**. QTAIM molecular graphs with bond paths and bond critical points (orange balls) without (a) and (c), and with (b) and (d) GD3BJ dispersion correction activated during the isolated-molecule geometry optimisations at B3LYP/def2-TZVP level of theory.

In the QTAIM molecular graphs, the bifurcated or three-fold C…Na bonding appears to be atom…atom directed. Therefore, we study the ELI-D distributions in addition, which show where electron pairs are localized and how they are directed. Figure S65 (a) shows that there is an electron pair per deprotonated carbon atom, which is populated with 2.0 e and a high localization value of 2.1 (unitless ELI-D value). This means that in agreement with the DI values, there is little charge sharing between C and Na atoms. Nevertheless, the basin of this electron pair is trisynaptic, which means that it shares a boundary (zero-flux surface in the topology) with three core basins: the carbon atom and the two Na atoms. This is a known signature of 3-center-2-electron bonding.[17] The shape of the localization domain of this electron pair, pointing towards the centre of the Na…Na axis, with flattened regions towards the Na atoms, also hints towards 3-center-2-electron bonding.

For the *(tetramer)*, the situation is similar, here pointing towards 4-center-2-electron bonding (Figure S65 (b)) as each electron pair of the deprotonated carbon atoms shares boundaries in the topological analysis with three Na atoms and its parent C atom. The cut-plane chosen allows to see two of such electron pairs, pointing to the centre of a Na-Na-Na triangle each. The center of this triangle and one of the Na atoms are in the same cut-plane; the other two Na atoms above and below. However, as for the *(dimer)*, there is only little charge transfer from the carbon atom to the Na atoms, with the electron pair being highly localized (ELI-D value = 2.1) and fully populated (2.1 e). This means that in total the carbon atoms in both *(dimer)* and *(tetramer)* are not hypervalent despite the hypercoordination as they are synaptically linked to four valence electron pairs with *ca.* 2.0 e each in a tetrahedral geometry, preserving $sp^3$ hybridization.

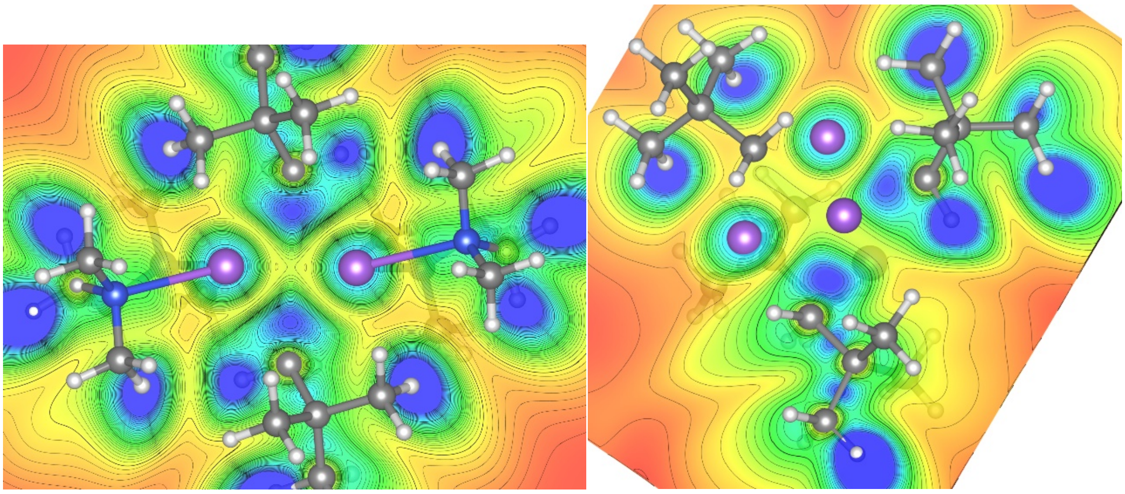

(a) (b)

*Figure S67* Contour map of ELI-D on a cut-plane through the central Na cluster for (a) the *(dimer)* and (b) the *(tetramer)*. Colour range from ELI-D = 0 (red, no localization) to 2 (blue, high localization), contour interval 0.1. Atom colors: purple = Na, grey = C, blue = N, white = H.

**Table S1**. Cartesian atom coordinates in Å for **1a** after geometry optimization at the B3LYP/def2-TZVP level of theory with GD3BJ dispersion correction.

| Tag | Symbol | X | Y | Z |
|---|---|---|---|---|
| 1 | Na | 2.6811560 | 0.6455200 | 15.4517020 |
| 2 | Na | 3.2150110 | 2.7059930 | 13.4009350 |
| 3 | C | 2.9523850 | 0.1825310 | 12.9626690 |
| 4 | C | 5.7040880 | 3.1688200 | 13.6725600 |
| 5 | C | 1.5138390 | 0.0298170 | 12.4229530 |
| 6 | C | 6.2434790 | 3.3225380 | 12.2340280 |
| 7 | C | 0.5642320 | -0.3698570 | 13.5623270 |
| 8 | C | 7.3461120 | 4.3937770 | 12.1386480 |
| 9 | C | 5.1040300 | 3.7233470 | 11.2849930 |
| 10 | C | 1.0291420 | 1.3642210 | 11.8343590 |
| 11 | C | 1.4178960 | -1.0417060 | 11.3206390 |
| 12 | C | 6.8315020 | 1.9882930 | 11.7481800 |
| 13 | H | 7.7351320 | 4.5036430 | 11.1202520 |
| 14 | H | 1.7250590 | 1.7274260 | 11.0719970 |
| 15 | H | 6.5904510 | 2.9849590 | 14.3113720 |
| 16 | H | 5.4663010 | 3.9224580 | 10.2731650 |
| 17 | H | 2.0735820 | -0.7863130 | 10.4852650 |
| 18 | H | 3.2677810 | -0.8305220 | 13.2841000 |
| 19 | H | 4.6090610 | 4.6334280 | 11.6376560 |
| 20 | H | 0.4767570 | 0.4282550 | 14.3075680 |

| | | | | |
|---|---|---|---|---|
| 21 | H | 0.9159750 | -1.2801700 | 14.0575640 |
| 22 | H | 5.3827780 | 4.1816320 | 13.9888750 |
| 23 | H | 3.5915010 | 0.3656870 | 12.0763840 |
| 24 | H | 0.3994920 | -1.1509420 | 10.9314540 |
| 25 | H | 7.3056160 | 2.0839280 | 10.7679310 |
| 26 | H | 4.3585570 | 2.9255000 | 11.1968400 |
| 27 | H | 0.9232540 | 2.1285280 | 12.6124450 |
| 28 | H | 8.1815540 | 4.1376090 | 12.7939630 |
| 29 | H | 1.7384800 | -2.0120350 | 11.7060530 |
| 30 | H | 6.9611060 | 5.3639990 | 12.4600490 |
| 31 | H | 7.5940570 | 1.6244400 | 12.4435730 |
| 32 | H | -0.4477220 | -0.5681680 | 13.1999260 |
| 33 | H | 6.0531520 | 1.2243010 | 11.6422010 |
| 34 | H | 0.0490010 | 1.2691110 | 11.3599090 |
| 35 | Na | 5.2655110 | 0.6455200 | 13.9350590 |
| 36 | Na | 4.7316570 | 2.7059930 | 15.9858260 |
| 37 | C | 4.9942820 | 0.1825310 | 16.4240930 |
| 38 | C | 2.2425800 | 3.1688200 | 15.7142020 |
| 39 | C | 6.4328290 | 0.0298180 | 16.9638090 |
| 40 | C | 1.7031900 | 3.3225380 | 17.1527330 |
| 41 | C | 7.3824360 | -0.3698560 | 15.8244340 |
| 42 | C | 0.6005570 | 4.3937770 | 17.2481140 |
| 43 | C | 2.8426380 | 3.7233460 | 18.1017680 |
| 44 | C | 6.9175260 | 1.3642210 | 17.5524020 |
| 45 | C | 6.5287720 | -1.0417060 | 18.0661220 |
| 46 | C | 1.1151660 | 1.9882930 | 17.6385820 |
| 47 | H | 0.2115370 | 4.5036430 | 18.2665110 |
| 48 | H | 6.2216080 | 1.7274270 | 18.3147650 |
| 49 | H | 1.3562170 | 2.9849590 | 15.0753900 |
| 50 | H | 2.4803680 | 3.9224570 | 19.1135960 |
| 51 | H | 5.8730860 | -0.7863130 | 18.9014960 |
| 52 | H | 4.6788870 | -0.8305220 | 16.1026620 |
| 53 | H | 3.3376080 | 4.6334270 | 17.7491050 |
| 54 | H | 7.4699100 | 0.4282560 | 15.0791930 |
| 55 | H | 7.0306930 | -1.2801700 | 15.3291970 |
| 56 | H | 2.5638900 | 4.1816320 | 15.3978870 |

| | | | | |
|---|---|---|---|---|
| 57 | H | 4.3551670 | 0.3656870 | 17.3103770 |
| 58 | H | 7.5471760 | -1.1509410 | 18.4553070 |
| 59 | H | 0.6410520 | 2.0839280 | 18.6188310 |
| 60 | H | 3.5881120 | 2.9254990 | 18.1899210 |
| 61 | H | 7.0234130 | 2.1285280 | 16.7743170 |
| 62 | H | -0.2348850 | 4.1376100 | 16.5927990 |
| 63 | H | 6.2081880 | -2.0120350 | 17.6807080 |
| 64 | H | 0.9855630 | 5.3639990 | 16.9267140 |
| 65 | H | 0.3526100 | 1.6244400 | 16.9431890 |
| 66 | H | 8.3943900 | -0.5681670 | 16.1868350 |
| 67 | H | 1.8935160 | 1.2243000 | 17.7445600 |
| 68 | H | 7.8976670 | 1.2691120 | 18.0268520 |

**Table S2**. Cartesian atom coordinates in Å for **1b** after geometry optimization at the B3LYP/def2-TZVP level of theory with GD3BJ dispersion correction.

| Tag | Symbol | X | Y | Z |
|---|---|---|---|---|
| 1 | Na | 4.8518330 | 9.3334020 | 5.7377540 |
| 2 | N | 6.2041240 | 9.1421010 | 3.6465400 |
| 3 | N | 7.3116710 | 9.6349820 | 6.4508550 |
| 4 | C | 3.6098000 | 6.2876990 | 6.3705350 |
| 5 | C | 3.5414600 | 4.9167480 | 5.6664400 |
| 6 | H | 2.5204040 | 4.7126100 | 5.3362080 |
| 7 | H | 3.8593580 | 4.0922810 | 6.3166930 |
| 8 | H | 4.1803650 | 4.9114640 | 4.7795900 |
| 9 | C | 3.1371440 | 7.4419440 | 5.4683910 |
| 10 | C | 7.4619330 | 9.8179500 | 3.9584710 |
| 11 | H | 8.1932810 | 9.6915880 | 3.1423050 |
| 12 | H | 7.2568510 | 10.8870880 | 4.0250240 |
| 13 | C | 8.1022330 | 9.3366380 | 5.2546590 |
| 14 | H | 8.2447400 | 8.2564880 | 5.2117340 |
| 15 | H | 9.1091920 | 9.7798050 | 5.3311180 |
| 16 | C | 6.4075280 | 7.7692100 | 3.2009600 |
| 17 | H | 5.4409920 | 7.2949190 | 3.0376120 |
| 18 | H | 6.9889230 | 7.7213680 | 2.2656380 |

| | | | | |
|---|---|---|---|---|
| 19 | H | 6.9311690 | 7.1892980 | 3.9587920 |
| 20 | C | 5.0638740 | 6.5200830 | 6.8060520 |
| 21 | H | 5.4590040 | 5.6804690 | 7.3864730 |
| 22 | H | 5.1412820 | 7.4123730 | 7.4332490 |
| 23 | H | 5.7140660 | 6.6515390 | 5.9362760 |
| 24 | C | 7.3757310 | 11.0519500 | 6.7994530 |
| 25 | H | 8.4081560 | 11.3732810 | 7.0122110 |
| 26 | H | 6.7649730 | 11.2381320 | 7.6807290 |
| 27 | H | 6.9773210 | 11.6665470 | 5.9953540 |
| 28 | C | 2.7431960 | 6.2216220 | 7.6335890 |
| 29 | H | 1.7074150 | 5.9898050 | 7.3767240 |
| 30 | H | 2.7551840 | 7.1832410 | 8.1532320 |
| 31 | H | 3.0927870 | 5.4502530 | 8.3270880 |
| 32 | C | 5.4345820 | 9.8888950 | 2.6575210 |
| 33 | H | 5.1963220 | 10.8797790 | 3.0429100 |
| 34 | H | 5.9725390 | 9.9941430 | 1.7015080 |
| 35 | H | 4.4994710 | 9.3647030 | 2.4647480 |
| 36 | C | 7.7594000 | 8.8239500 | 7.5780800 |
| 37 | H | 7.6688320 | 7.7663270 | 7.3350810 |
| 38 | H | 7.1347310 | 9.0280820 | 8.4480190 |
| 39 | H | 8.8059630 | 9.0335150 | 7.8516890 |
| 40 | H | 2.0839480 | 7.2315450 | 5.2059510 |
| 41 | H | 3.6572960 | 7.3386690 | 4.4974030 |
| 42 | Na | 2.0446220 | 9.7126670 | 5.7408390 |
| 43 | N | 0.6494420 | 9.9019090 | 3.6725070 |
| 44 | N | -0.3999470 | 9.4251780 | 6.5059250 |
| 45 | C | 3.2948850 | 12.7582070 | 6.3856380 |
| 46 | C | 3.3598260 | 14.1323020 | 5.6874950 |
| 47 | H | 4.3783800 | 14.3360540 | 5.3493470 |
| 48 | H | 3.0493210 | 14.9542100 | 6.3445120 |
| 49 | H | 2.7131910 | 14.1431090 | 4.8063320 |
| 50 | C | 3.7564270 | 11.6070420 | 5.4737530 |
| 51 | C | -0.6174820 | 9.2583860 | 4.0166770 |
| 52 | H | -1.3663830 | 9.4078950 | 3.2206990 |
| 53 | H | -0.4398910 | 8.1838670 | 4.0733740 |
| 54 | C | -1.2110490 | 9.7498790 | 5.3307270 |

| | | | | |
|---|---|---|---|---|
| 55 | H | -1.3261100 | 10.8336760 | 5.2964830 |
| 56 | H | -2.2271480 | 9.3324350 | 5.4299730 |
| 57 | C | 0.4677980 | 11.2783810 | 3.2280100 |
| 58 | H | 1.4409210 | 11.7305840 | 3.0424840 |
| 59 | H | -0.1329990 | 11.3375840 | 2.3057230 |
| 60 | H | -0.0257250 | 11.8713550 | 3.9959480 |
| 61 | C | 1.8446240 | 12.5259430 | 6.8329830 |
| 62 | H | 1.4558050 | 13.3630960 | 7.4212050 |
| 63 | H | 1.7720990 | 11.6303040 | 7.4556500 |
| 64 | H | 1.1864990 | 12.3996900 | 5.9683460 |
| 65 | C | -0.4698700 | 8.0036620 | 6.8347890 |
| 66 | H | -1.5018220 | 7.6862560 | 7.0556560 |
| 67 | H | 0.1500930 | 7.8011720 | 7.7059320 |
| 68 | H | -0.0851440 | 7.3975740 | 6.0177210 |
| 69 | C | 4.1726080 | 12.8167840 | 7.6414010 |
| 70 | H | 5.2062090 | 13.0496770 | 7.3770820 |
| 71 | H | 4.1645640 | 11.8523870 | 8.1559510 |
| 72 | H | 3.8293340 | 13.5844850 | 8.3420840 |
| 73 | C | 1.3736860 | 9.1334400 | 2.6660620 |
| 74 | H | 1.6030080 | 8.1400520 | 3.0504960 |
| 75 | H | 0.8052350 | 9.0338200 | 1.7272710 |
| 76 | H | 2.3132400 | 9.6363980 | 2.4411710 |
| 77 | C | -0.8204730 | 10.2223200 | 7.6533820 |
| 78 | H | -0.7295980 | 11.2827990 | 7.4235420 |
| 79 | H | -0.1796790 | 10.0026810 | 8.5076540 |
| 80 | H | -1.8625000 | 10.0137990 | 7.9445640 |
| 81 | H | 4.8071060 | 11.8166360 | 5.2009190 |
| 82 | H | 3.2261560 | 11.7161170 | 4.5087920 |

# X-ray Crystallographic Details

### Table S3. Selected crystallographic parameters

| Compound | 1a | 1b | 3a |
|---|---|---|---|
| CCDC number | 2447797 | 2447132 | 2442917 |
| Empirical formula | $C_{20}H_{44}Na_4$ | $C_{11}H_{27}N_2Na$ | $C_{38}H_{72}N_6Na_2$ |
| Formula weight | 376.51 | 210.33 | 658.99 |
| Temperature/K | 173.00(10) | 173.00(10) | 173.00(10) |
| Crystal system | monoclinic | orthorhombic | triclinic |
| Space group | P2/n | Pmmn | P-1 |
| a/Å | 10.15035(16) | 13.77748(12) | 9.7495(4) |
| b/Å | 6.15814(8) | 12.70033(16) | 9.8940(4) |
| c/Å | 20.1832(3) | 8.51923(8) | 11.3857(5) |
| α/° | 90 | 90 | 82.329(3) |
| β/° | 103.9117(15) | 90 | 87.210(3) |
| γ/° | 90 | 90 | 85.299(3) |
| Volume/Å$^3$ | 1224.59(3) | 1490.68(3) | 1084.00(7) |
| Z | 2 | 4 | 1 |
| $\rho_{calc}$g/cm$^3$ | 1.021 | 0.937 | 1.009 |
| μ/mm$^{-1}$ | 1.043 | 0.669 | 0.077 |
| F(000) | 416.0 | 472.0 | 364.0 |
| Crystal size/mm$^3$ | 1.596 × 0.165 × 0.141 | 0.235 × 0.161 × 0.128 | 0.332 × 0.249 × 0.181 |
| Radiation | Cu Kα (λ = 1.54184) | Cu Kα (λ = 1.54184) | Mo Kα (λ = 0.71073) |
| 2Θ range for data collection/° | 9.028 to 149.136 | 9.472 to 148.854 | 4.166 to 61.012 |
| Index ranges | -12 ≤ h ≤ 12, -7 ≤ k ≤ 7, -25 ≤ l ≤ 24 | -16 ≤ h ≤ 17, -15 ≤ k ≤ 15, -10 ≤ l ≤ 10 | -13 ≤ h ≤ 13, -14 ≤ k ≤ 14, -16 ≤ l ≤ 16 |
| Reflections collected | 25550 | 31669 | 32817 |
| Independent reflections | 2505 [$R_{int}$ = 0.0660, $R_{sigma}$ = 0.0445] | 1665 [$R_{int}$ = 0.0288, $R_{sigma}$ = 0.0113] | 6605 [$R_{int}$ = 0.0463, $R_{sigma}$ = 0.0369] |
| Data/restraints/parameters | 2505/0/197 | 1665/33/153 | 6605/203/310 |
| Goodness-of-fit on F$^2$ | 1.045 | 1.091 | 1.044 |
| Final R indexes [I>=2σ (I)] | $R_1$ = 0.0571, $wR_2$ = 0.1475 | $R_1$ = 0.0398, $wR_2$ = 0.1348 | $R_1$ = 0.0731, $wR_2$ = 0.2173 |
| Final R indexes [all data] | $R_1$ = 0.0576, $wR_2$ = 0.1478 | $R_1$ = 0.0425, $wR_2$ = 0.1384 | $R_1$ = 0.1175, $wR_2$ = 0.2494 |
| Largest diff. peak/hole / e Å$^{-3}$ | 0.41/-0.28 | 0.21/-0.14 | 0.53/-0.22 |
| Flack parameter | - | - | - |

**Table S4. Selected crystallographic parameters**

| Compound | 3b | 4a | 4b |
|---|---|---|---|
| CCDC number | 2442906 | 2442915 | 2442916 |
| Empirical formula | $C_{48}H_{92}N_6Na_2O_2$ | $C_{32}H_{64}N_6Na_2$ | $C_{30}H_{64}N_6Na_2$ |
| Formula weight | 831.25 | 578.87 | 554.85 |
| Temperature/K | 173.00(10) | 173.00(10) | 173.00(10) |
| Crystal system | monoclinic | triclinic | triclinic |
| Space group | $P2_1/n$ | P-1 | P-1 |
| a/Å | 12.24787(11) | 9.4466(2) | 11.0628(2) |
| b/Å | 12.65883(13) | 10.0325(3) | 11.4227(2) |
| c/Å | 16.92535(14) | 10.8310(3) | 15.0760(3) |
| α/° | 90 | 65.888(3) | 82.2455(17) |
| β/° | 93.4268(8) | 82.916(2) | 74.1737(18) |
| γ/° | 90 | 75.224(2) | 76.9069(18) |
| Volume/$Å^3$ | 2619.47(4) | 905.73(4) | 1779.80(7) |
| Z | 2 | 1 | 2 |
| $\rho_{calc}g/cm^3$ | 1.054 | 1.061 | 1.035 |
| $\mu/mm^{-1}$ | 0.634 | 0.084 | 0.681 |
| F(000) | 920.0 | 320.0 | 616.0 |
| Crystal size/$mm^3$ | 0.177 × 0.147 × 0.12 | 0.424 × 0.264 × 0.183 | 0.409 × 0.255 × 0.222 |
| Radiation | Cu Kα (λ = 1.54184) | Mo Kα (λ = 0.71073) | Cu Kα (λ = 1.54184) |
| 2Θ range for data collection/° | 8.67 to 149 | 4.122 to 61.01 | 7.97 to 148.97 |
| Index ranges | -15 ≤ h ≤ 15, -15 ≤ k ≤ 13, -21 ≤ l ≤ 21 | -13 ≤ h ≤ 13, -14 ≤ k ≤ 14, -15 ≤ l ≤ 15 | -13 ≤ h ≤ 13, -12 ≤ k ≤ 14, -18 ≤ l ≤ 18 |
| Reflections collected | 41470 | 55967 | 59508 |
| Independent reflections | 5312 [$R_{int}$ = 0.0358, $R_{sigma}$ = 0.0206] | 5527 [$R_{int}$ = 0.0377, $R_{sigma}$ = 0.0213] | 7185 [$R_{int}$ = 0.0319, $R_{sigma}$ = 0.0142] |
| Data/restraints/parameters | 5312/0/274 | 5527/247/354 | 7185/114/476 |
| Goodness-of-fit on $F^2$ | 1.040 | 1.042 | 1.042 |
| Final R indexes [I>=2σ (I)] | $R_1$ = 0.0403, $wR_2$ = 0.1145 | $R_1$ = 0.0504, $wR_2$ = 0.1387 | $R_1$ = 0.0415, $wR_2$ = 0.1112 |
| Final R indexes [all data] | $R_1$ = 0.0451, $wR_2$ = 0.1181 | $R_1$ = 0.0727, $wR_2$ = 0.1511 | $R_1$ = 0.0448, $wR_2$ = 0.1137 |
| Largest diff. peak/hole / e $Å^{-3}$ | 0.22/-0.16 | 0.44/-0.17 | 0.32/-0.36 |
| Flack parameter | - | - | - |

**Crystal-Structure Determination of 1a.** A crystal of $C_{20}H_{44}Na_4$ immersed in parabar oil was mounted at ambient conditions and transferred into the stream of nitrogen (173 K). All measurements were made on a *RIGAKU Synergy S* area-detector diffractometer[29] using mirror optics monochromated Cu *K*α radiation (λ = 1.54184 Å). The unit cell constants and an orientation matrix for data collection were obtained from a least-squares refinement of the setting angles of reflections in the range 4.507° < θ < 78.550°. A total of 6514 frames were collected using ω scans, with 0.2 seconds exposure time (1.0 s for high-angle reflections), a rotation angle of 0.5° per frame, a crystal-detector distance of 31.0 mm, at T = 173.00(10) K.

Data reduction was performed using the *CrysAlisPro*[29] program. The intensities were corrected for Lorentz and polarization effects, and an analytical numeric absorption correction using a multifaceted crystal model based on expressions derived by R.C. Clark & J.S. Reid[33] with additional empirical absorption correction using spherical harmonics, as implemented in SCALE3 ABSPACK scaling algorithm in *CrysAlisPro*[29] was applied. Data collection and refinement parameters are given in *Table S1.*

The structure was solved by intrinsic phasing using *SHELXT*[30], which revealed the positions of all non-hydrogen atoms of the title compound. All non-hydrogen atoms were refined anisotropically. H-atoms were located from the difference density map and had their positions and isotropic displacement parameters refined freely.

Refinement of the structure was carried out on $F^2$ using full-matrix least-squares procedures, which minimized the function $\Sigma w(F_o^2 - F_c^2)^2$. The weighting scheme was based on counting statistics and included a factor to downweight the intense reflections. All calculations were performed using the *SHELXL-2014/7*[31] program in OLEX2. [32]

Twinning could be detected where the second component corresponds to a rotation of 77.5643 degrees around [0.00 -1.00 0.00] (reciprocal space), or [0.00 -1.00 -0.00] (direct space), with a volume fractional contribution of 0.2193. The refinement was performed against the reflection file containing detwinned data of the major component only.

**Crystal-Structure Determination of 1b.** A crystal of $C_{11}H_{27}N_2Na$ immersed in parabar oil was mounted at 173K using the X-TEMP2[34][35] cold temperature device under the microscope, carried to the diffractometer inside a dewar containing liquid nitrogen and then transferred into a stream of gaseous nitrogen (173 K). All measurements were made on a *RIGAKU Synergy S* area-detector diffractometer[29] using mirror optics monochromated Cu *K*α radiation (λ = 1.54184 Å). The unit cell constants and an orientation matrix for data collection were obtained from a least-squares refinement of the setting angles of reflections in the range

4.741° < θ < 79.552°. A total of 6152 frames were collected using ω scans, with 0.8 second exposure time (3.0 s for high-angle reflections), a rotation angle of 0.5° per frame, a crystal-detector distance of 31.0 mm, at T = 173.00(10) K.

Data reduction was performed using the *CrysAlisPro*[29] program. The intensities were corrected for Lorentz and polarization effects, and an analytical numeric absorption correction using a multifaceted crystal model based on expressions derived by R.C. Clark & J.S. Reid[33] with additional empirical absorption correction using spherical harmonics, as implemented in SCALE3 ABSPACK scaling algorithm in *CrysAlisPro*[29] was applied. Data collection and refinement parameters are given in *Table S1.*

The structure was solved by intrinsic phasing using *SHELXT*[30], which revealed the positions of all non-hydrogen atoms of the title compound. All non-hydrogen atoms were refined anisotropically. H-atoms were assigned in geometrically calculated positions and refined using a riding model where each H-atom was assigned a fixed isotropic displacement parameter with a value equal to 1.2Ueq of its parent atom (1.5Ueq for methyl groups).

Refinement of the structure was carried out on $F^2$ using full-matrix least-squares procedures, which minimized the function $\Sigma w(F_o^2 - F_c^2)^2$. The weighting scheme was based on counting statistics and included a factor to downweight the intense reflections. All calculations were performed using the *SHELXL-2014/7*[31] program in OLEX2.[32]

The entire molecule is disordered about a symmetry element with multiplicity 2, therefore all atoms were refined as negative parts with occupancy set to 50% (kept fixed during the refinement. Additional dynamic disorder was used for the structure where the occupancies of each disorder component was refined through the use of a free variable. The sum of equivalent components was constrained to 0.5, i.e. 50% (due to the symmetry element). Commensurate modulation was detected where the refined 1d 1$^{st}$ order modulation vector corresponded to 0.0011(5) -2.9e-03(7) 0.4993(5). The refinement was performed for the average structure, leaving therefore all modulation effects unmodeled and reflected on the disorder model. Due to the setting of negative parts, checkcif fails to see the connectivity correct leading to alerts for crystal density, therefore, a solvent mask was used to search for possible voids and include their electron density contribution into the calculated structure factors. As expected, no voids were found, but the mask was kept to suppress checkcif alerts.

**Crystal-Structure Determination of 3a.** A crystal of $C_{38}H_{72}N_6Na_2$ immersed in parabar oil was mounted at 173K using the X-TEMP2[34][35] cold temperature device under the microscope, carried to the diffractometer inside a dewar containing liquid nitrogen and then

transferred into a stream of gaseous nitrogen (173 K). All measurements were made on a *RIGAKU XtaLAB Synergy R*, HyPix-Arc 100 area-detector diffractometer[29] using mirror optics monochromated Mo *K*α radiation (λ = 0.71073 Å). The unit cell constants and an orientation matrix for data collection were obtained from a least-squares refinement of the setting angles of reflections in the range 2.692° < θ < 29.763°. A total of 1722 frames were collected using ω scans, with 5 seconds exposure time, a rotation angle of 0.5° per frame, a crystal-detector distance of 43.0 mm, at T = 173.00(10) K.

Data reduction was performed using the *CrysAlisPro*[29] program. The intensities were corrected for Lorentz and polarization effects, and a numerical absorption correction based on gaussian integration over a multifaceted crystal model with additional empirical absorption correction using spherical harmonics using SCALE3 ABSPACK in *CrysAlisPro*[29] was applied. Data collection and refinement parameters are given in *Table S1.*

The structure was solved by intrinsic phasing using *SHELXT*[30], which revealed the positions of all non-hydrogen atoms of the title compound. All non-hydrogen atoms were refined anisotropically. H-atoms were assigned in geometrically calculated positions and refined using a riding model where each H-atom was assigned a fixed isotropic displacement parameter with a value equal to 1.2Ueq of its parent atom (1.5Ueq for methyl groups).

Refinement of the structure was carried out on $F^2$ using full-matrix least-squares procedures, which minimized the function $\Sigma w(F_o^2 - F_c^2)^2$. The weighting scheme was based on counting statistics and included a factor to downweight the intense reflections. All calculations were performed using the *SHELXL-2014/7*[32] program in OLEX2. [33]

Disorder model was used for parts of the structure where the occupancies of each disorder component was refined through the use of a free variable. The sum of equivalent components was constrained to 1, i.e. 100%.

**Crystal-Structure Determination of 3b.** A crystal of $C_{48}H_{92}N_6Na_2O_2$ immersed in parabar oil was mounted at 173K using the X-TEMP2[34][35] cold temperature device under the microscope, carried to the diffractometer inside a dewar containing liquid nitrogen and then transferred into a stream of gaseous nitrogen (173 K). All measurements were made on a *RIGAKU Synergy S* area-detector diffractometer[29] using mirror optics monochromated Cu *K*α radiation (λ = 1.54184 Å). The unit cell constants and an orientation matrix for data collection were obtained from a least-squares refinement of the setting angles of reflections in the range 4.345° < θ < 79.992°. A total of 6360 frames were collected using ω scans, with 0.5 seconds exposure time

(1.5 s for high-angle reflections), a rotation angle of 0.5° per frame, a crystal-detector distance of 31.0 mm, at T = 173.00(10) K.

Data reduction was performed using the *CrysAlisPro*[29] program. The intensities were corrected for Lorentz and polarization effects, and a numerical absorption correction based on gaussian integration over a multifaceted crystal model with additional empirical absorption correction using spherical harmonics using SCALE3 ABSPACK in *CrysAlisPro*[29] was applied. Data collection and refinement parameters are given in *Table S2.*

The structure was solved by intrinsic phasing using *SHELXT*[30], which revealed the positions of all non-hydrogen atoms of the title compound. All non-hydrogen atoms were refined anisotropically. H-atoms were assigned in geometrically calculated positions and refined using a riding model where each H-atom was assigned a fixed isotropic displacement parameter with a value equal to 1.2Ueq of its parent atom (1.5Ueq for methyl groups).

Refinement of the structure was carried out on $F^2$ using full-matrix least-squares procedures, which minimized the function $\Sigma w(F_o^2 - F_c^2)^2$. The weighting scheme was based on counting statistics and included a factor to downweight the intense reflections. All calculations were performed using the *SHELXL-2014/7*[34] program in OLEX2. [35]

**Crystal-Structure Determination of 4a.** A crystal of $C_{32}H_{64}N_6Na_2$ immersed in parabar oil was mounted at 173K using the X-TEMP2[34][35] cold temperature device under the microscope, carried to the diffractometer inside a dewar containing liquid nitrogen and then transferred into a stream of gaseous nitrogen (173 K). All measurements were made on a *RIGAKU XtaLAB Synergy R*, HyPix-Arc 100 area-detector diffractometer[29] using mirror optics monochromated Mo *K*α radiation (λ = 0.71073 Å). The unit cell constants and an orientation matrix for data collection were obtained from a least-squares refinement of the setting angles of reflections in the range 2.055° < θ < 32.447°. A total of 3466 frames were collected using ω scans, with 0.4 seconds exposure time, a rotation angle of 0.5° per frame, a crystal-detector distance of 43.0 mm, at T = 173.00(10) K.

Data reduction was performed using the *CrysAlisPro*[29] program. The intensities were corrected for Lorentz and polarization effects, and a numerical absorption correction based on gaussian integration over a multifaceted crystal model with additional empirical absorption correction using spherical harmonics using SCALE3 ABSPACK in *CrysAlisPro*[29] was applied. Data collection and refinement parameters are given in *Table S2.*

The structure was solved by intrinsic phasing using *SHELXT*[30], which revealed the positions of all non-hydrogen atoms of the title compound. All non-hydrogen atoms were refined

anisotropically. H-atoms were assigned in geometrically calculated positions and refined using a riding model where each H-atom was assigned a fixed isotropic displacement parameter with a value equal to 1.2Ueq of its parent atom (1.5Ueq for methyl groups).

Refinement of the structure was carried out on $F^2$ using full-matrix least-squares procedures, which minimized the function $\Sigma w(F_o^2 - F_c^2)^2$. The weighting scheme was based on counting statistics and included a factor to downweight the intense reflections. All calculations were performed using the *SHELXL-2014/7*[31] program in OLEX2. [32]

Disorder model was used for parts of the structure where the occupancies of each disorder component was refined through the use of a free variable. The sum of equivalent components was constrained to 1, i.e. 100%.

**Crystal-Structure Determination of 4b.** A crystal of $C_{30}H_{64}N_6Na_2$ immersed in parabar oil was mounted at 173K using the X-TEMP2[34][35] cold temperature device under the microscope, carried to the diffractometer inside a dewar containing liquid nitrogen and then transferred into a stream of gaseous nitrogen (173 K). All measurements were made on a *RIGAKU Synergy S* area-detector diffractometer[29] using mirror optics monochromated Cu *K*α radiation (λ = 1.54184 Å). The unit cell constants and an orientation matrix for data collection were obtained from a least-squares refinement of the setting angles of reflections in the range 7.389° < θ < 80.483°. A total of 11384 frames were collected using ω scans, with 0.5 second exposure time (2.0 s for high-angle reflections), a rotation angle of 0.5° per frame, a crystal-detector distance of 31.0 mm, at T = 173.00(10) K.

Data reduction was performed using the *CrysAlisPro*[29] program. The intensities were corrected for Lorentz and polarization effects, and a numerical absorption correction based on gaussian integration over a multifaceted crystal model with additional empirical absorption correction using spherical harmonics using SCALE3 ABSPACK in *CrysAlisPro*[29] was applied. Data collection and refinement parameters are given in *Table S2.*

The structure was solved by intrinsic phasing using *SHELXT*[30], which revealed the positions of all non-hydrogen atoms of the title compound. All non-hydrogen atoms were refined anisotropically. H-atoms were assigned in geometrically calculated positions and refined using a riding model where each H-atom was assigned a fixed isotropic displacement parameter with a value equal to 1.2Ueq of its parent atom (1.5Ueq for methyl groups).

Refinement of the structure was carried out on $F^2$ using full-matrix least-squares procedures, which minimized the function $\Sigma w(F_o^2 - F_c^2)^2$. The weighting scheme was based on counting

statistics and included a factor to downweight the intense reflections. All calculations were performed using the *SHELXL-2014/7*[31] program in OLEX2.[32]

Disorder model was used for parts of the structure where the occupancies of each disorder component was refined through the use of a free variable. The sum of equivalent components was constrained to 1, i.e. 100%. Twinning could be detected where the second component corresponds to a rotation of 3.2750 degrees around -0.63 -0.71 -0.30 (reciprocal space), or -0.67 -0.74 -0.01 (direct space), with a volume fractional contribution of 0.3892. The refinement was performed against the reflection file containing detwinned data of the major component only.

## Space filling model of 4b

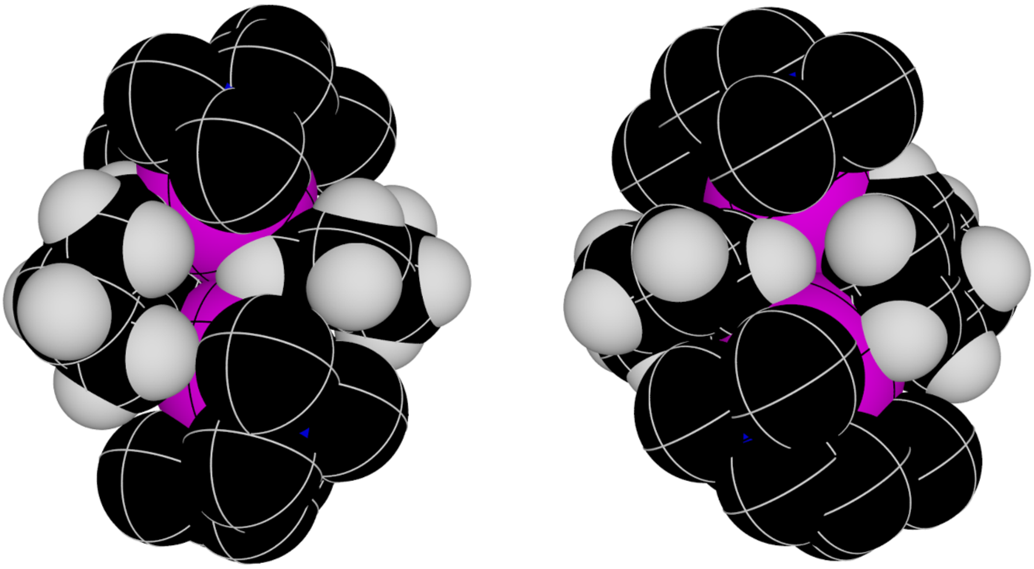

*Figure S68 Space filing model showing steric congestion around the two sodium centres.*

# References


[1] P. Benrath, M. Kaiser, T. Limbach, M. Mondeshki, J. Klett, *Angew. Chemie - Int. Ed.* **2016**, *55*, 10886–10889.

[2] A. Tortajada, D. E. Anderson, E. Hevia, *Helv. Chim. Acta* **2022**, *105*, DOI 10.1002/hlca.202200060.

[3] N. Trongsiriwat, M. Li, A. Pascual-Escudero, B. Yucel, P. J. Walsh, *Adv. Synth. Catal.* **2019**, *361*, 502–509.

[4] R. Neufeld, D. Stalke, *Chem. Sci.* **2015**, *6*, 3354–3364.

[5] C. Santilli, I. S. Makarov, P. Fristrup, R. Madsen, *J. Org. Chem.* **2016**, *81*, 9931–9938.

[6] M. Shi, Y. Feng, *J. Org. Chem.* **2001**, *102*, 3235–3237.

[7] M. Schlosser, H. C. Jung, S. Takagishi, *Tetrahedron* **1990**, *46*, 5633–5648.

[8] T. J. Pearson, R. Shimazumi, J. L. Driscoll, B. D. Dherange, D. Il Park, M. D. Levin, *Science (80-. ).* **2023**, *381*, 1474–1479.

[9] X. Wang, R. X. Chen, Z. F. Wei, C. Y. Zhang, H. Y. Tu, A. D. Zhang, *J. Org. Chem.* **2016**, *81*, 238–249.

[10] L. Gremaud, A. Alexakis, *Angew. Chemie - Int. Ed.* **2012**, *51*, 794–797.

[11] J. Gu, H. Yang, J. Deng, D. Jiang, K. Lv, T. Wang, Q. Yao, *Org. Chem. Front.* **2024**, *11*, DOI 10.1039/d3qo02099a.

[12] R. Knorr, E. C. Rossmann, M. Knittl, *Synthesis (Stuttg).* **2010**, *12*, 2124–2128.

[13] V. D. Vitnik, M. D. Ivanovic, Z. J. Vitnik, J. B. Orevic, Z. S. Zizak, Z. D. Juranic, I. O. Juranic, *Synth. Commun.* **2009**, *39*, 1457–1471.

[14] J. Palaty, F. S. Abbott, *J. Med. Chem.* **1995**, *38*, 3398–3406.

[15] J. D. Goodreid, J. Janetzko, J. P. Santa Maria, K. S. Wong, E. Leung, B. T. Eger, S. Bryson, E. F. Pai, S. D. Gray-Owen, S. Walker, W. A. Houry, R. A. Batey, *J. Med. Chem.* **2016**, *59*, 624–646.

[16] D. N. Zalatan, J. Du Bois, *J. Am. Chem. Soc.* **2009**, *131*, 7558–7559.

[17] G. Dilauro, A. F. Quivelli, P. Vitale, V. Capriati, F. M. Perna, *Angew. Chemie - Int. Ed.* **2019**, *58*, 1799–1802.

[18] Gaussian 09, Revision D.01, M. J. Frisch, G. W. Trucks, H. B. Schlegel, G. E. Scuseria, M. A. Robb, J. R. Cheeseman, G. Scalmani, V. Barone, G. A. Petersson, H. Nakatsuji, X. Li, M. Caricato, A. Marenich, J. Bloino, B. G. Janesko, R. Gomperts, B. Mennucci, H. P. Hratchian, J. V. Ortiz, A. F. Izmaylov, J. L. Sonnenberg, D. Williams-Young, F. Ding, F. Lipparini, F. Egidi, J. Goings, B. Peng, A. Petrone, T. Henderson, D. Ranasinghe, V. G. Zakrzewski, J. Gao, N. Rega, G. Zheng, W. Liang, M. Hada, M. Ehara, K. Toyota, R. Fukuda, J. Hasegawa, M. Ishida, T. Nakajima, Y. Honda, O. Kitao, H. Nakai, T. Vreven, K. Throssell, J. A. Montgomery, Jr., J. E. Peralta, F. Ogliaro, M. Bearpark, J. J. Heyd, E. Brothers, K. N. Kudin, V. N. Staroverov, T. Keith, R. Kobayashi, J. Normand, K. Raghavachari, A. Rendell, J. C. Burant, S. S. Iyengar, J. Tomasi, M. Cossi, J. M. Millam, M. Klene, C. Adamo, R. Cammi, J. W. Ochterski, R. L. Martin, K. Morokuma, O. Farkas, J. B. Foresman, and D. J. Fox, Gaussian, Inc., Wallingford CT, **2016**.

[19] S. Grimme, S. Ehrlich, L. Georigk, *J. Comput. Chem.* **2011**, *32*, 1456–1465.

[20] S. Grabowsky (editor), Complementary bonding analysis, de Gruyter, Berlin, Boston, **2021**.

[21] R. F. W. Bader, *Acc. Chem. Res.* **1985**, *18*, 9–15.

[22] A. Martín Pendas, C. Gatti, Quantum theory of atoms in molecules and the AIMAll software, in Complementary bonding analysis, editor: S. Grabowsky, de Gruyter, Berlin, Boston, **2021** pp. 43-74.

[23] E. R. Johnson, S. Keinan, P. Mori-Sánchez, J. Contreras-García, A. J. Cohen, W. Yang, *J. Am. Chem. Soc.* **2010**, *132*, 6498–6506.

[24] T. Lu, F. Chen, *J. Comput. Chem.* **2012**, *33*, 580–592.

[25] W. Humphrey, A. Dalke, K. Schulten, *J. Mol. Graph.* **1996**, *14*, 33–38.

[26] M. Kohout, *Int. J. Quantum Chem.* **2004**, *97*, 651–658.

[27] M. Kohout, Electron localizability indicator and bonding analysis with DGrid, in Complementary bonding analysis, editor: S. Grabowsky, de Gruyter, Berlin, Boston, **2021** pp. 75-112.

[28] K. Momma, F. Izumi, *J. Appl. Crystallogr.* **2008**, *41*, 653–658.

[29] Oxford Diffraction (2018). *CrysAlisPro* (Version 1.171.40.37a). Oxford Diffraction Ltd., Yarnton, Oxfordshire, UK.

[30] Sheldrick, G. M. (2015). *Acta Cryst.* **A71**, 3-8.

[31] Sheldrick, G. M. (2015). *Acta Cryst*. **C71**, 3-8.

[32] Dolomanov, O.V., Bourhis, L.J., Gildea, R.J, Howard, J.A.K. & Puschmann, H. (2009), J. Appl. Cryst. 42, 339-341.

[33] Clark, R. C. & Reid, J. S. (1995). Acta Cryst. A51, 887-897.

[34] T. Kottke and D. Stalke, *J. Appl. Cryst.* 1993, **26**, 615–619.

[35] D. Stalke, *Chem. Soc. Rev.* 1998, **27**, 171–178.
